# Identifying neurophenotypes of major depressive disorder through normative model of regional homogeneity

**DOI:** 10.1038/s41398-026-04003-8

**Published:** 2026-04-09

**Authors:** Zhanjie Luo, Weicheng Li, Yubing Xu, Junhao Shen, Chengyu Wang, Xiaofeng Lan, Guanxi Liu, Zhanhui Luo, Zhaoyi Hou, Siming Mai, Muqin Zhang, Xiangdong Sun, Hanna Lu, Yanling Zhou, Yuping Ning

**Affiliations:** 1 The Affiliated Brain Hospital, Guangzhou Medical University, Guangzhou, China; 2 Guangdong Engineering Technology Research Center for Translational Medicine of Mental Disorders, Guangzhou, China; 3 Guangzhou Key Clinical Specialty (Clinical Medical Research Institute), Guangzhou, China; 4 School of Mental Health, Guangzhou Medical University, Guangzhou, China; 5 Sihui People’s Hospital, Sihui, China; 6 Guangdong-Hong Kong-Macao Greater Bay Area Center for Brain Science and Brain-Inspired Intelligence, Southern Medical University, Guangzhou, China; 7 Department of Psychiatry, The Chinese University of Hong Kong, Hong Kong SAR, China

**Keywords:** Depression, Pathogenesis

## Abstract

Major depressive disorder (MDD) is a highly heterogeneous mental illness, marked by clinical variability and distinct neuropathological mechanisms. This study sought to enhance diagnostic precision for MDD by identifying neurophenotypes using a normative model of regional homogeneity (ReHo), offering new avenues for precision medicine. Using resting-state functional magnetic resonance imaging data from 1101 patients with MDD and 1011 healthy controls (HCs) sourced from the REST-meta-MDD project, we developed the first normative model of ReHo. Gaussian process regression was applied to predict a normative lifespan trajectory based on age and sex in HCs, enabling quantification of individual deviations from the model for patients with MDD. Unsupervised clustering algorithms were then employed to classify MDD subtypes, followed by validation analyses to assess clustering stability. Significant deviations from the normative ReHo model were observed in patients with MDD. Two distinct MDD subtypes emerged: Emotional dysregulation subtype, characterized by negative deviations in the frontoparietal control network, ventral attention network, default mode network, and limbic network (Cohen’s *d* = 0.40−1.75, FDR-corrected *p* < 0.05). This subtype correlated with more severe overall depressive symptom (*d* = 0.17, *p* = 0.010), better insight (*d* = −0.25, *p* = 0.009), younger age (*d* = −0.19, *p* = 0.003), lower medication usage (Cramer’s *V* = 0.09, *p* = 0.017), a negative correlation between symptom severity and illness duration (*r* = −0.21, *p* < 0.001), severe brain dysfunction (Partial *η*^*2*^ = 0.00–0.01, FDR-corrected *p* < 0.05), and higher neural vulnerability (positive: 0.25%–4.03%, *d* = 0.12, FDR-corrected *p* = 0.177; negative: 0.25%–2.01%, *d* = 0.31, FDR-corrected *p* < 0.05). Perceptual dysregulation subtype, defined by negative deviations in the sensorimotor network, visual network, and dorsal attention network (*d* = 0.52–1.81, FDR-corrected *p* < 0.05). This subtype was associated with more severe anxiety/somatization symptoms (*d* = −0.15, *p* = 0.031), older age, higher medication usage, poorer insight, and stable neural vulnerability (positive: 0.14%–2.98%, *d* = 0.08, FDR-corrected *p* = 0.333; negative: 0.14%–1.42%, *d* = −0.24, FDR-corrected *p* < 0.05). These neuroimaging distinctions corresponded to clinical differences between subtypes, illuminating the heterogeneity of MDD. The findings emphasize the necessity of personalized interventions tailored to the unique neuropathological mechanisms of each subtype, advancing precision medicine in MDD.

## Introduction

Major depressive disorder (MDD) is a serious mental disorder that affects about 332 million people globally [1]. Its onset can occur across the lifespan, from adolescence [2] to old age [3], indicating its broad impact on psychiatric disorder that impacts individuals mental health across different age groups [4]. In addition, MDD shows marked sex differences in prevalence, suggesting a potential role for sex-specific factors in its etiology and susceptibility [5]. Despite its widespread prevalence, the considerable heterogeneity of MDD presents major challenges for diagnosis and treatment [6]. This heterogeneity manifests in diverse clinical symptoms, such as depressed mood, loss of interest and sleep disturbances, as well as varying neuropathological mechanisms among individuals with the same diagnosis [7], complicating effective treatment strategies. Identifying precise subtypes is essential for improving clinical classification and enabling personalized treatment by elucidating the underlying mechanisms of MDD. Notably, the human cortex is organized along a hierarchical unimodal-to-transmodal gradient, extending from primary sensorimotor areas to transmodal association regions. This principal axis supports a spectrum of functions from basic perception to abstract, cross-modal cognition [8]. The disruptions along this transmodal hierarchy provide a unifying framework for understanding the diverse pathophysiology of MDD.

In recent years, resting-state functional magnetic resonance imaging (rs-fMRI) has advanced the study of neuropathological mechanisms and cognitive behavioural processes in MDD [2]. Regional homogeneity (ReHo), a robust metric in rs-fMRI research, has demonstrated high test-retest reliability [9]. ReHo measures the synchrony of neural activity within local brain regions [10], effectively reflecting local connectivity [11]. Abnormal ReHo patterns have been linked to MDD development, particularly in key regions such as the dorsolateral prefrontal cortex (DLPFC), middle temporal gyrus, anterior insula, and hippocampus [12–15]. Dysfunctions in these regions may drive the neuropathological processes underlying MDD, contributing to global brain network disruptions and clinical symptoms [16].

Despite its potential, research on ReHo in MDD is limited by small sample sizes, leading to inconsistent findings. Additionally, most studies rely on case-control designs, which, while effective in highlighting differences between patients with MDD and healthy controls (HCs), often fail to capture the disorder’s heterogeneity [17], thereby limiting our understanding of the different subtypes of the disorder.

Modelling individual deviations from normative brain function offers a novel approach to exploring MDD’s heterogeneity [18]. Gaussian process regression (GPR), a Bayesian nonparametric method, is particularly suited for constructing normative models, as it provides coherent confidence measures alongside point estimates [19]. Normative modelling has significant advantages over traditional case-control studies. For example, ReHo varies across the lifespan, influenced by factors like age [20] and sex [21]. Similar to growth charts used to assess a child’s development relative to population norms, a normative ReHo model incorporating these factors can better capture the lifespan trajectory in HCs [22]. Deviations from this normative trajectory in patients with MDD reflect the extent of neuropathological abnormalities, offering a foundation for precise diagnosis and personalized treatment.

To date, no study has developed a normative ReHo model for MDD. This study aims to address this gap by using a large HC dataset to construct a ReHo normative model and identify individual deviations in patients with MDD. By leveraging age- and sex-based predictions, we capture the normative ReHo trajectory across the lifespan. Quantifying deviations in patients with MDD allows us to classify subtypes, offering deeper insights into the heterogeneity of MDD and paving the way for improved clinical classification, precise diagnosis, and personalized treatment strategies.

## Methods and materials

Our data statistics and visualization strategies were guided by the approach presented in SUN et al. [23], which provides a comprehensive framework for analyzing this type of data.

### Dataset details and image preprocessing

Utilizing data from the REST-meta-MDD project, the study was approved by the Ethics Committee of 25 hospitals in the DIRECT Consortium, with all participants providing written informed consent. Table S1 provides detailed information on sample sizes, participating sites, cohort characteristics, and data collection parameters for the 25 study groups from 17 Chinese hospitals. Comprehensive project details are available at https://rfmri.org/REST-meta-MDD [24]. The data supporting the findings of this study are openly available in the Psychological Science Data Bank at 10.57760/sciencedb.o00115.00013. From the 1300 patients with MDD and 1128 HCs originally recruited, 1101 patients with MDD and 1011 HCs from 22 sites were included in this analysis. Selection criteria, adapted from Yan et al. [16], involved exclusion based on factors such as duplicate data, missing demographic information, poor image quality, excessive head motion, abnormal regional spatial correlation, and sites with fewer than 10 subjects (Figure S1). Demographic and clinical details of the participants are presented in Table S2. All rs-fMRI data were preprocessed by trained neuroimaging analysts using a standardized pipeline (Supplement).

### Normative modeling for ReHo in HCs

**Fig. 1:**
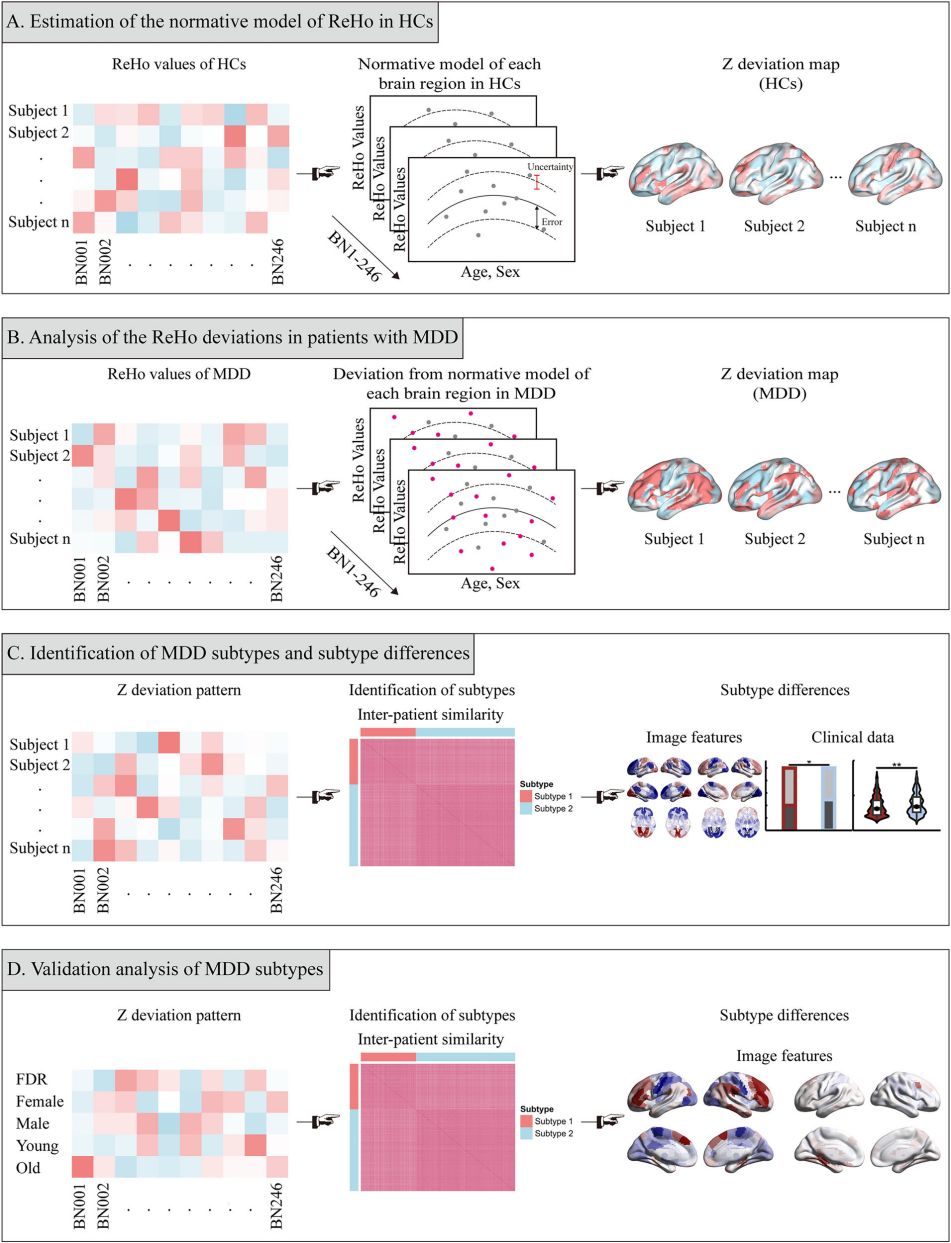
Process pipeline. **A** A normative model of Regional Homogeneity (ReHo) was constructed using Gaussian process regression on ReHo values from healthy controls (gray dots). The solid line represents predicted ReHo values, and the dashed line shows the normative range, generating a Z deviation map for HCs. **B** The normative model was applied to patients with MDD (pink dots) to generate individual whole-brain deviation maps. Error represents the confidence interval, and uncertainty reflects model estimation uncertainty. **C** Subtypes were identified based on whole-brain ReHo deviation patterns in patients with MDD, with subtype differences being characterized based on ReHo deviation patterns. **D** Validation of clustering results across different thresholds and populations. HCs healthy controls, MDD major depressive disorder, BN human brainnetome atlas.

To create the normative model, we first extracted regional ReHo values for 246 brain areas using the Human Brainnetome Atlas [25] (Supplement). Site-specific effects were harmonized using ComBat harmonization [26] to ensure comparability across data from multiple sites. GPR was then applied to model the normative trajectory of ReHo as a function of age and sex (Fig. 1A). Specifically, the Gaussian process model (Supplement) is a method that enables Bayesian nonlinear regression on a set of input data (i.e., clinical covariates) to predict trends in neurobiological variables (i.e., neuroimaging data). This approach provides not only point estimates but also confidence intervals, which are interpretable as the normative range for predicted values [22]. Individual deviation maps for HCs were constructed by estimating deviations from the predicted norms (Supplement). The normative model’s performance was evaluated using 10-fold cross-validation (Supplement). Metrics such as standardized mean squared error (SMSE) and mean standardized log loss (MSLL) were used to assess model accuracy (Supplement).

### Estimating ReHo deviations from the normative model in patients with MDD

Using the normative model derived from the HC group, we calculated deviations for each brain region in the MDD group, generating individual deviation maps (Fig. 1B) (Supplement). Deviations were quantified as Z-scores, with extreme deviations defined as values exceeding ±2.6 (*p* < 0.005), following established methodologies [23, 27]. We assessed inter-patient heterogeneity and group-level differences using metrics such as deviation indices, extreme deviation counts, spatial overlap maps, and mean deviation maps (Supplement). Between-group differences were evaluated using independent-sample t-tests, with significance thresholds corrected for multiple comparisons via the Benjamini–Hochberg false discovery rate (FDR) procedure (FDR-corrected *p* < 0.05).

### Identifying MDD subtypes based on ReHo deviations

To classify MDD subtypes, a k-means clustering algorithm was applied to deviation patterns (Fig. 1C). The optimal number of clusters was determined using the NbClust package, employing a majority-vote mechanism (Supplement). Inter-patient similarity within clusters was assessed using Euclidean distance. To ensure the robustness of the clustering results, we compared subtype distributions across individual sites and the overall population. We also conducted clustering analyses in each site and compared the subtype distributions between individual sites and the whole population. Leave-one-site-out validation was conducted to confirm the stability of the findings (Supplement). Importantly, sites with fewer than 30 patients were excluded, and then the clustering analysis and leave-one-site-out validation were repeated. Differences between subtypes were characterized using imaging features and clinical data.

### Validation analysis of MDD subtypes

Validation analyses were performed to further confirm the robustness of the subtypes. These included a stricter definition of Z-deviation values and subgroup-specific normative modeling based on age and sex, and validation within key clinical subgroups (i.e., first-episode drug-naïve (FEDN), recurrent, and medicated patients) (Fig. 1D). Overlap rates between clustering labels derived from subgroup analyses and primary results as well as the adjusted Rand indices (ARI) were calculated to evaluate the stability of the clustering results (Supplement).

## Results

### Normative modeling for ReHo in HCs

**Fig. 2:**
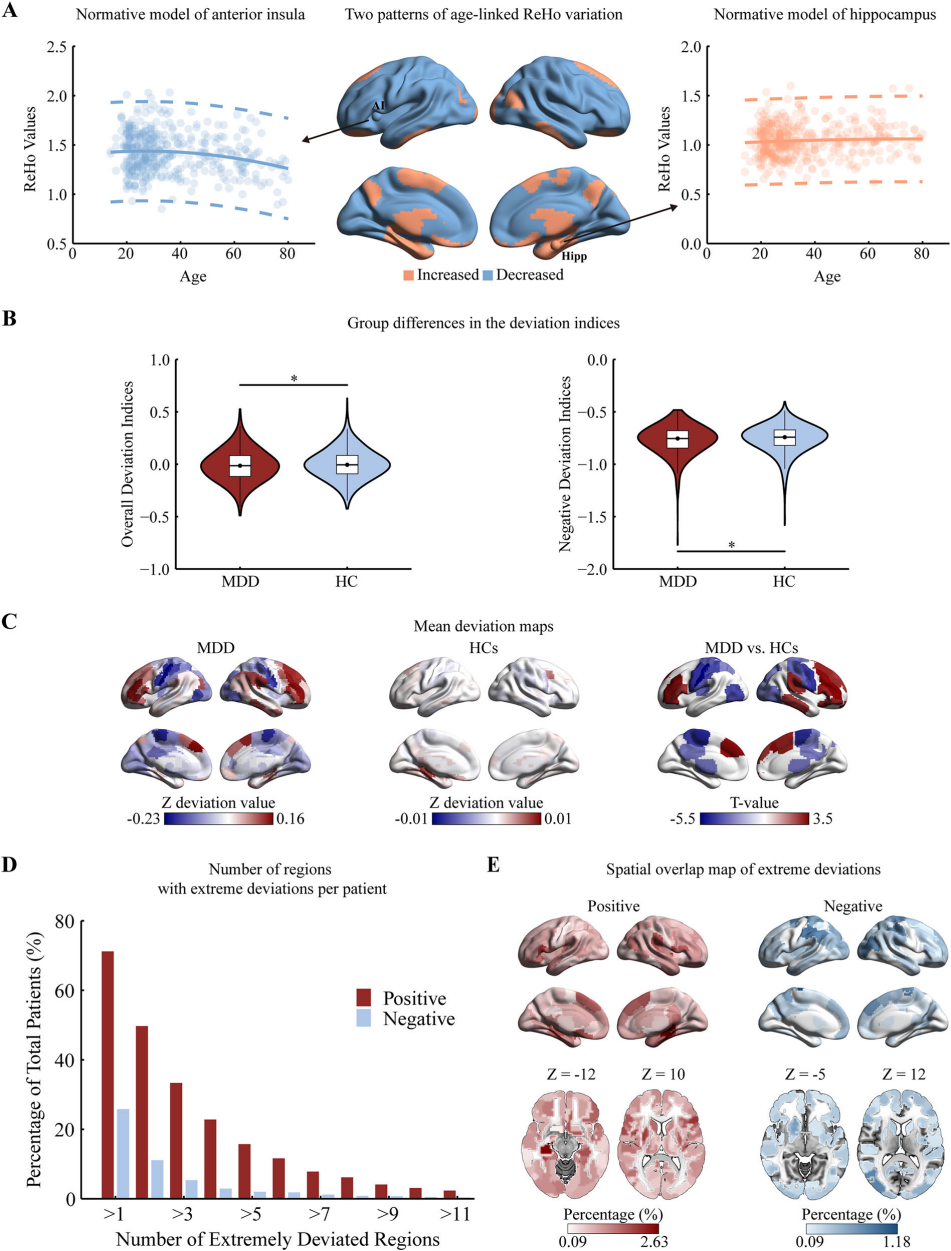
Normative model and ReHo deviation in patients with MDD. **A** Age-related ReHo variation in HCs (male) is shown, categorized into decreasing (blue) and increasing (orange) patterns using k-means clustering. Scatter plots display true ReHo values for the anterior insula (left) and hippocampus (right) in HCs, with the solid line representing the predicted mean and dashed lines showing the normative ranges. **B** Group differences in deviation indices between patients with MDD and HCs. **C** Mean deviation maps for patients with MDD and HCs, with between-group differences (FDR-corrected *p* < 0.05). **D** Number of brain regions with extreme deviations per patient. **E** Spatial overlap map showing extreme positive (red) and negative (blue) deviations across all patients with MDD.

The normative model of ReHo in HCs was predicted based on age and sex. The SMSE of the model was 0.968 ± 0.039, indicating high prediction accuracy, while the MSLL was −0.016 ± 0.021, reflecting reasonable uncertainty in the predictions (Figure S3). We visualized the variation trajectories and normative ranges of ReHo in the anterior insula and hippocampus in terms of the predicted mean, predicted variance, and Z = 2.6 according to a medical reference value formula [28] (Fig. 2A). In the normative model, we found that both male (Fig. 2A) and female (Figure S4) could be clustered (Supplement) into two distinct patterns of age-related ReHo variation. Regions showing increased age-related ReHo were predominantly located in the temporal cortex, lateral occipital cortex, hippocampal gyrus, amygdala, precuneus, and fusiform gyrus. In contrast, regions with decreased ReHo were observed in the prefrontal cortex, sensorimotor cortex, parietal cortex, insula, basal ganglia, and cingulate gyrus.

### Significant deviations from the normative model in patients with MDD

Compared with HCs, patients with MDD exhibited significantly more negative deviation indices overall (Cohen’s *d* = −0.09) and for negative deviations specifically (*d* = −0.09) (Fig. 2B). No significant differences were found in positive deviation indices or in the extreme deviation counts across the three categories (FDR-corrected *p* < 0.05).

For whole-brain ReHo deviations, the MDD group displayed pronounced deviations with a hemispheric lateralization pattern. Positive deviations were concentrated in the left DLPFC and right temporal cortex, primarily within the frontoparietal control network (FPCN) and limbic network (LN). Negative deviations were observed in the bilateral sensorimotor areas, cingulate gyrus, parahippocampal gyrus, occipital lobes, parietal lobes, and right prefrontal lobe, which are part of the sensorimotor network (SMN), visual network (VN), default mode network (DMN), and subcortical subregions (SUB) (Fig. 2C; Table S3) (absolute *d* = 0.15–0.24, FDR-corrected *p* < 0.05).

Among patients with MDD, 76.02% (*n* = 837) exhibited at least one brain region with an extreme deviation from the normative model. Specifically, 71.21% (*n* = 784) showed extreme positive deviations, while 25.79% (*n* = 284) had extreme negative deviations. Across all brain regions (*n* = 246), at least one extreme deviation was present in every patient (extreme positive: 100%; extreme negative: 79.67%, *n* = 196). Positive deviations were most prevalent in the prefrontal cortex and hippocampal gyrus, while negative deviations were concentrated in sensorimotor areas (Fig. 2E). At the regional level, no more than 2.63% (*n* = 29) of patients with MDD exceeded extreme positive deviation, and no more than 1.18% (*n* = 13) exceeded extreme negative deviation per brain region (Fig. 2E).

### Identification of MDD subtypes based on ReHo deviation patterns

**Fig. 3:**
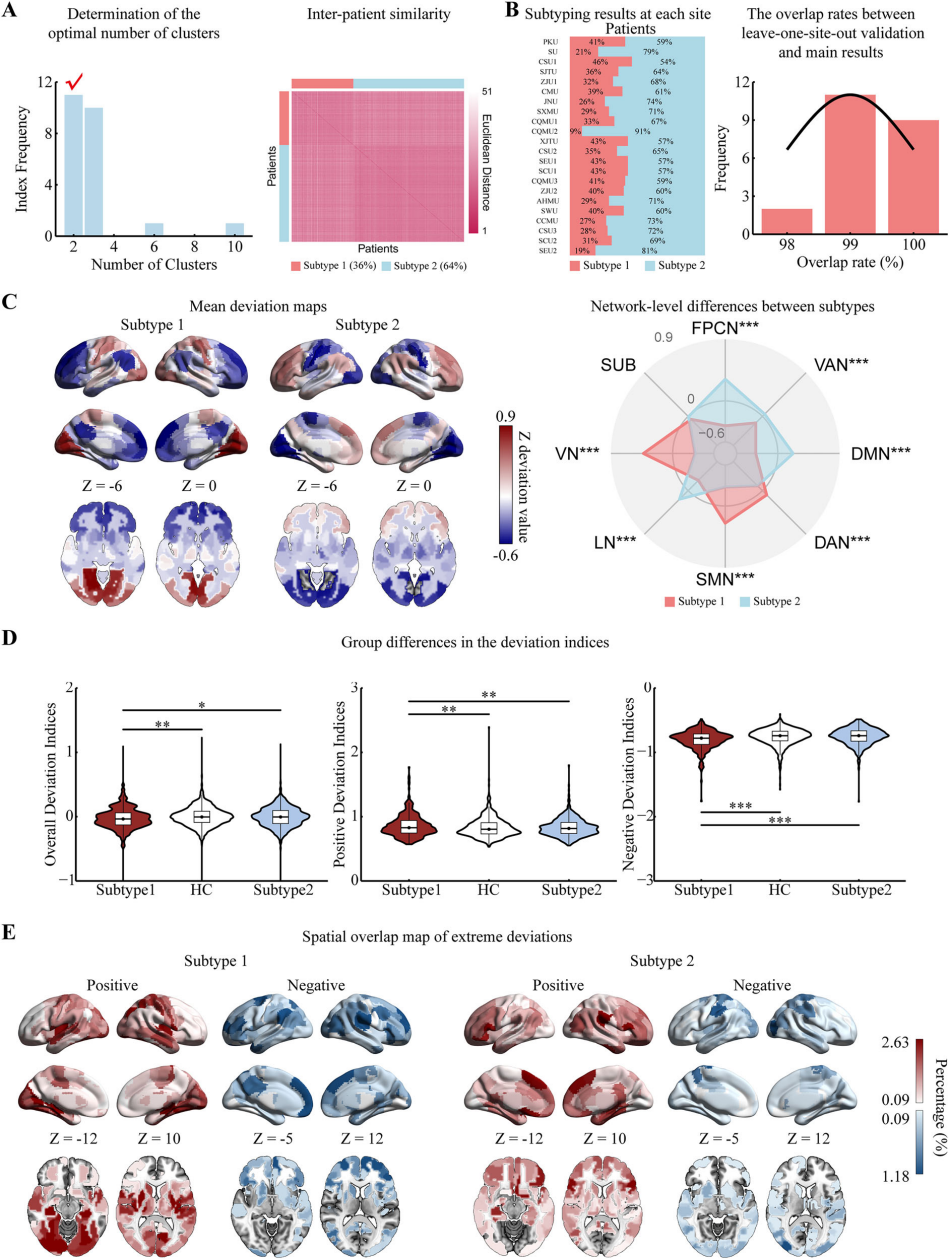
Identification of MDD subtypes based on Z deviation patterns of ReHo. **A** The optimal number of MDD clusters was determined using the NbClust package, with inter-patient similarity assessed by Euclidean distance. **B** Subtyping results from k-means clustering analysis at each site, showing the overlap rates (rounded) between site-specific clustering labels and primary results from leave-one-site-out validation. **C** Mean deviation maps for each subtype and their network-level differences. **D** Group differences in deviation indices among MDD subtypes and HCs. **E** Spatial overlap map of extreme positive (red) and negative (blue) deviations for each subtype. *** FDR-corrected *p* < 0.001, ** *p* < 0.01, * *p* < 0.05. Sites: PKU Peking University, SU Soochow University, CSU Central South University, SJTU Shanghai Jiao Tong University, ZJU Zhejiang University, CMU China Medical University, JNU Jinan University, SXMU Shanxi Medical University, CQMU Chongqing Medical University, XJTU Xi’an Jiaotong University, SEU Southeast University, SCU Sichuan University, AHMU Anhui Medical University, SWU Southwest University, CCMU Capital Medical University. Networks: FPCN frontoparietal control network, VAN ventral attention network, DMN default mode network, DAN dorsal attention network, SMN sensorimotor network, LN limbic network, VN visual network, SUB subcortical regions.

Using k-means clustering (NbClust package) [29], patients with MDD were classified into two subtypes, as indicated by 11 of 23 indices (Fig. 3A). Subtype 1 comprised 36% (*n* = 397) of patients, while subtype 2 represented 64% (*n* = 704). Euclidean distance metrics showed considerable similarity within subtypes and high heterogeneity between them (Fig. 3A). We calculated the percentage of two subtypes at each site (Table S4) and after k-means clustering at each site (Table S5). Similar analyses were performed after excluding sites with <30 patients with MDD (Figure S5; Table S6, Table S7; Supplement). In the overall population, the overlap rate between clustering labels obtained from the leave-one-site-out validation and the primary results was > 98% in all cases (Fig. 3B). After excluding sites with fewer than 30 patients, this overlap rate was > 94% in all cases.

At brain- and network levels, the two subtypes showed opposing deviation patterns. For brain regions, subtype 1 showed positive deviation in regions such as the parietal cortex, occipital cortex, and sensorimotor areas and negative deviations in the lateral and medial prefrontal cortex, anterior insula, and hippocampus, whereas subtype 2 displayed the opposite pattern (Fig. 3C). At the network level, subtype 1 exhibited positive deviation in the dorsal attention network (DAN), SMN, and VN and negative deviation in the ventral attention network (VAN), FPCN, DMN, and LN, and vice versa for subtype 2 (Fig. 3C; Table S8) (absolute *d* = 0.40–1.81, *p* < 0.05, FDR-corrected for comparisons among the networks).

One-way analysis of variance revealed that subtype 1 had lower overall and negative deviation indices but higher positive deviation indices than HCs (Partial *η*^*2*^ = 0.00–0.02, FDR-corrected *p* < 0.05), whereas subtype 2 showed no significant differences from HCs across the three indices (Fig. 3D; Table S9). In addition, subtype 1 had more extreme overall and negative deviation counts than HCs (Partial *η*^*2*^ = 0.00–0.01, FDR-corrected *p* < 0.05), whereas the extreme positive deviation counts of subtype 1 and all three extreme deviation counts of subtype 2 were not statistically different from HCs (Figure S6; Table S10) (Supplement).

Spatial overlap maps highlighted that subtype 1 exhibited greater disease impact, with higher rates of extreme deviations than all patients with MDD (positive: 0.25%–4.03%, *d* = 0.12, FDR-corrected *p* = 0.177; negative: 0.25%–2.01%, *d* = 0.31, FDR-corrected *p* < 0.05). In contrast, subtype 2 demonstrated lower sensitivity (positive: 0.14%–2.98%, *d* = 0.08, FDR-corrected *p* = 0.333; negative: 0.14%–1.42%, *d* = −0.24, FDR *p* < 0.05) (Fig. 3E).

**Fig. 4:**
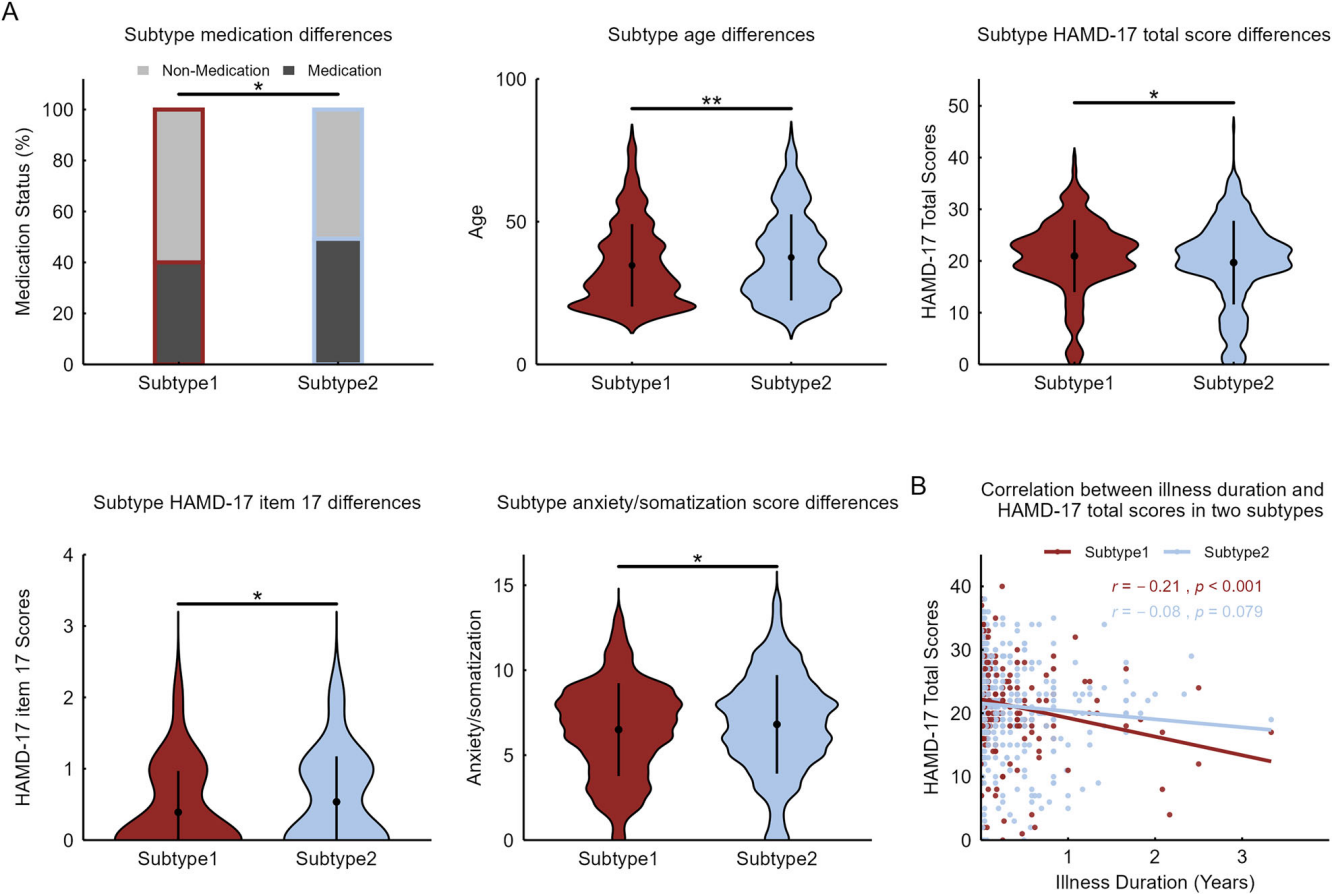
Subtype differences in demographic and clinical data. **A** Differences in age, medication status, the Hamilton Depression Rating Scale-17 item (HAMD-17) total score, the anxiety/somatization factor score, and the HAMD-17 item 17 score across subtype. **B** Correlation between illness duration (years) and HAMD-17 total score in the two subtypes. ** *p* < 0.01. * *p* < 0.05.

Subtype 1 patients were younger (*d* = −0.19, *p* = 0.003) and had lower medication rates (Cramer’s *V* = 0.09, *p* = 0.017) than patients with subtype 2 (Fig. 4A; Table S11). Subtype 1 patients had more severe overall depressive symptoms, as measured by the Hamilton Depression Rating Scale-17 item (HAMD-17) total score (*d* = 0.17, *p* = 0.010), than subtype 2 patients but showed better insight according to the HAMD-17 item 17 (*d* = −0.25, *p* = 0.009) (Fig. 4A; Table S11). Analysis of HAMD-17 symptom factors (anxiety/somatization, weight, cognitive impairment, retardation, sleep disturbance [13]) revealed that Subtype 2 scored significantly higher on the anxiety/somatization factor (*d* = −0.15, *p* = 0.031) (Fig. 4A; Table S11), with no significant differences on other factors (all *p* > 0.05). Furthermore, analysis of covariance revealed a significant main effect of illness duration on the HAMD-17 total score (*η*_*p*_^*2*^ = 0.02, *p* < 0.001) but no significant interaction effect with subtype (*η*_*p*_^*2*^ = 0.00, *p* = 0.132) (Table S12). In subtype 1, the HAMD-17 total score was negatively associated with illness duration (*r* = -0.21, *p* < 0.001), whereas no such correlation was observed in subtype 2 (*r* = −0.08, *p* = 0.079) (Fig. 4B). Finally, we performed a leave-one-site-out validation in all patients and sites with <30 patients, which showed that these differences between two subtypes were largely maintained (Figure S7, Figure S8; Table S13, Table S14).

### High consistency of primary results in validation analysis

We performed a validation analysis using a stricter definition of extreme deviation (FDR-corrected *p* < 0.05) and performed subgroup analyses for male, female, young, and old participants, as well as for key clinical subgroups (FEDN, recurrent, and medicated patients). The overlap rates between clustering labels derived from each subgroup and those from the primary results exceeded 89% (range: 89.61%–98.87%). In addition, no significant site-related effects were observed on Z-deviation values across any of the 246 brain regions in the whole brain, with FDR-corrected *p*-values ranging from 0.108 to 1.000. Detailed results of the validation analysis are presented in the Supplement.

## Discussion

In this study, we identified MDD subtypes using a multi-site, large-sample normative model of ReHo, addressing the significant heterogeneity in MDD. Our findings demonstrated the ability to characterize ReHo variation trajectories and define normative ranges in HCs. Furthermore, we quantified deviations in patients with MDD and identified two distinct MDD subtypes, each associated with divergent neuroimaging features and clinical symptoms. These results offer valuable insights for advancing precision medicine approaches in MDD and provide critical clues.

### Normative modeling for ReHo in HCs

The human brain comprises a complex network of interconnected regions [30]. While recent research in functional connectomics has emphasized large-scale network connectivity [30, 31], the role of local functional connectivity has been relatively overlooked. Large-scale approaches have inherent limitations, whereas ReHo provides a valuable framework for identifying boundaries in functionally heterogeneous regions, enabling more precise brain parcellation [32].

To capture the intricate trajectories of ReHo variation with age, we employed GPR, a method well-suited for modelling nonlinear changes. GPR allows for the estimation of predicted means and uncertainty at any age [22, 23, 33], facilitating the identification of hidden patterns and anomalies. Using this approach, we constructed a normative model of ReHo based on age and sex in HCs, finding that both males and females could be grouped into two distinct patterns of age-related ReHo variation. A study showed that ReHo tends to decline linearly with age in adolescents, particularly in the prefrontal cortex, likely due to synaptic pruning and the strengthening of long-range functional connectivity [20]. Additionally, the parieto-frontal integration theory (P-FIT) suggests that intelligence and cognitive abilities are primarily supported by functional integration between the parietal and frontal lobes, improving neural network efficiency [34]. In line with these findings, our study observed age-related decreases in ReHo in the prefrontal cortex and parietal lobes, potentially reflecting a shift from local connectivity to broader network integration to support increasingly complex cognitive processes [20]. This transition enhances processing efficiency by coordinating the functions of diverse brain regions to meet cognitive demands at different life stages.

Conversely, studies of cognitively normal older adults have reported increases in hippocampal ReHo with age, potentially linked to tau protein deposition. This deposition may weaken long-range connectivity, enhance local connectivity, and impair episodic memory [35]. Similarly, our findings showed increased hippocampal ReHo with age, suggesting that these changes may reflect compensatory increases in local connectivity due to disrupted long-range functional networks and associated cognitive decline.

Thus, the relationship between ReHo and age demonstrates a complex, nonlinear trajectory, with variations that differ across brain regions. GPR effectively captured these dynamics, providing a robust framework for investigating local brain connectivity throughout the lifespan.

### Significant deviations from the normative model in patients with MDD

This study employed GPR to construct a normative model of ReHo based on HCs and quantify deviations from the model in patients with MDD. By incorporating additive noise into uncertainty estimates through predictive variance calculations, GPR offers robustness in identifying significant ReHo deviations, particularly under high-noise conditions like rs-fMRI [19, 22, 36].

The findings revealed significantly negative deviation indices in patients with MDD compared with HCs, reflecting systematic reductions in brain activity. However, there were no significant differences in extreme deviation counts between the groups, suggesting potential heterogeneity in MDD subtypes that might obscure functional differences.

Group-level analysis of individual deviation maps highlighted significant positive ReHo deviations in the right temporal cortex, including the middle temporal gyrus, parahippocampal gyrus, and fusiform gyrus. These results align with prior research suggesting that hypersynchronization in the middle temporal gyrus is a key mechanism underlying MDD, particularly its psychomotor retardation [13]. Increased ReHo in the parahippocampal gyrus has also been linked to MDD diagnosis, while reductions in this region may predict medication response [37]. Additionally, a study on late-life depression have implicated fusiform gyrus hypersynchronization in mediating dementia risk through its interplay with olfactory dysfunction [15]. These findings collectively suggest that ReHo alterations in the right temporal cortex may contribute to the neuropathological characteristics of MDD.

Negative ReHo deviations were observed in the sensorimotor and visual cortices, indicating reduced synchronization during complex sensory and motor integration processes, which are closely associated with MDD [38]. The posterior cingulate cortex (PCC), a critical hub within the DMN, also showed negative deviations [39], consistent with prior findings of reduced functional connectivity within the DMN in MDD [16].

A prominent feature of MDD is hypoconnectivity within the FPCN, particularly between the DLPFC and posterior parietal cortex (PPC), which is closely linked to impaired cognitive control and emotional regulation [40]. Our study supports this view as ReHo in the anterior FPCN (i.e. DLPFC) showed positive deviations, whereas that in the posterior FPCN (i.e. PCC) showed negative deviations. These opposing deviations suggest that hypersynchronization in the DLPFC and reduced local connectivity in the PPC disrupt overall network coordination, impairing cognitive control and emotional regulation

Overall, 76.02% of patients exhibited extreme ReHo deviations in at least one brain region, and every brain region (100%) had at least one patient showing such deviations. This widespread occurrence underscores the diffuse nature of neural dysfunction in MDD, reinforcing its characterization as a disorder of global brain disruptions [41].

### Identification of MDD subtypes based on ReHo deviation patterns

The integrity and connectivity of brain network nodes are critical for large-scale interactions underlying emotional processing, cognitive regulation, and perceptual responses. Local brain dysfunction often coexists with network-level abnormalities [11, 42]. Using k-means clustering, we identified two MDD subtypes based on ReHo deviation patterns, each characterized by distinct deviations at both node and network levels.

Key nodes within the FPCN, including the DLPFC and PPC, are critical for cognitive control over attention and emotion regulation [40]. The VAN combines the salience network (SN) and cingulo-opercular network [43, 44], with key nodes including the anterior insula and dorsal anterior cingulate cortex, which play pivotal roles in processing emotional changes and environmental stimuli [45]. The DMN, with nodes in the medial prefrontal cortex and PCC, is engaged during emotional regulation and self-referential processes [46, 47]. Lastly, the LN, with the hippocampus as a key node, is integral to emotional processing [48]. In subtype 1, these networks and their key nodes exhibited negative ReHo deviations, suggesting reduced local connectivity and deficits in emotional regulation and cognitive abilities. Termed the “emotional dysregulation subtype,” this pattern aligns with the triple network model [30], where disrupted reward responses in the LN impair motivation and anhedonia. This dysfunction exacerbates SN impairments, hindering effective activation of the FPCN and DMN, which are essential for cognitive and emotional regulation, thereby aggravating cognitive impairment and mood dysregulation in patients with MDD. Notably, consistent with the group-level analysis, the DMN exhibited reduced synchronization in the emotional dysregulation subtype.

A core hub of the SMN is the sensorimotor cortex, which is involved in bodily sensation and motor control [49]. The VN includes the occipital cortex, which is closely linked to visual perceptual abilities [50]. The DAN includes the PPC, which is involved in visuospatial perception and goal-directed tasks [51]. These three networks belong to the extrinsic system and work in coordination in perception of external information, motor execution, and spatial attention tasks [52, 53]. In subtype 2, these networks showed negative ReHo deviations, indicating reduced synchronization and impairments in sensory and perceptual processing. This pattern was named the “perceptual dysregulation subtype.”

In further analyses, we characterized the MDD subtypes using neuroimaging features and clinical symptoms. The results showed that the emotional dysregulation subtype was characterized by clinical features often seen in acute or early-stage illness (younger age, lower medication rates, more severe depressive symptoms) [54, 55]. These patients exhibited higher neural vulnerability and more extreme functional deviations, indicative of severe disruptions in emotional and cognitive regulation. Illness duration correlated negatively with depressive severity in this subtype, consistent with a pattern where longer illness duration was associated with lower symptom severity.

In contrast, the perceptual dysregulation subtype was linked to older age, higher medication rates, more severe anxiety/somatization symptoms, stable neural vulnerability, and fewer extreme functional deviations, aligning with a more chronic clinical presentation. The prominent anxiety/somatization observed in this subtype may be mechanistically rooted in its core neural deficit: reduced synchrony within the SMN. This is consistent with prior reports of decreased SMN functional connectivity in patients with somatization disorder [56] and with theoretical models that attribute altered interoception and somatosensory amplification to impaired integration within this network [57]. This subtype exhibit poorer insight, possibly in relation to DAN dysfunction, as observed in other disorders like schizophrenia [58–60], which is consistent with the observed lack of insight in this subtype.

The analysis of covariance revealed a significant main effect of illness duration on the HAMD-17 total scores, suggesting a general influence on depressive symptoms [61, 62]. In the emotional dysregulation subtype, a significant negative correlation was found between illness duration and HAMD-17 total scores, indicating that longer illness duration was associated with lower symptom severity. This is consistent with the profile of patients who exhibit lower medication rates and more severe baseline symptoms. In contrast, the correlation in the perceptual dysregulation subtype only approached significance, suggesting a more stable symptom trajectory, which fits the profile of a more chronic subtype.

To summarize, the emotional dysregulation subtype was associated with a clinical profile suggestive of a more acute phase of MDD, while the perceptual dysregulation subtype was associated with a more chronic, stable form, characterized by persistent neural abnormalities, more severe anxiety/somatization symptoms, and poorer insight. These findings emphasize the need to consider such distinct profiles for tailored interventions, advancing the field of personalized medicine in MDD.

### Neurophenotypes along a cortical hierarchical gradient

The opposing deviation patterns observed between higher-order (DMN, FPCN, VAN, LN) and lower-order (SMN, VN, DAN) networks align with established models of cortical hierarchical organization. The principal functional gradient of the cortex extends from unimodal regions, supporting sensory perception and attention, to transmodal regions underpinning abstract cognition and emotional regulation [8].

Within this framework, the emotional dysregulation subtype, characterized by negative deviations in transmodal networks, likely reflects a primary dysfunction in top-down integrative and regulatory processes [63]. Conversely, the perceptual dysregulation subtype, marked by negative deviations in unimodal and intermediate networks, suggests a disturbance in bottom-up sensory processing and attentional gating [64]. Thus, our findings position the two neurophenotypes at distinct loci along a cortical processing hierarchy, offering a parsimonious model for understanding heterogeneous pathophysiology in MDD as dysregulation of this fundamental functional axis.

### High consistency of primary results in validation analysis

Validation analysis confirmed the robustness of our primary findings, demonstrating overall label overlap > 73% and high ARI (≥0.79 in six of seven comparisons). The agreement was moderate for the recurrent group (ARI = 0.2). In addition, no significant differences were observed between sites in the Z-deviation value across brain regions, confirming the broad applicability and stability of the constructed model.

### Limitations and future directions

While this study utilized a large, multi-site dataset to construct a ReHo-based normative model for HCs and to uncover heterogeneity in MDD subtypes, several limitations must be acknowledged. First, the normative model and neurophenotypes were derived exclusively from a Chinese cohort. Given that normative modeling is inherently dependent on its reference population, this limits the direct extrapolation of our findings. A critical future direction is to test the robustness and generalizability of the identified ReHo normative model and the two neurophenotypes in multi-ethnic, international cohorts. Second, although GPR proved effective in this study, employing more advanced machine learning algorithms or higher-resolution neuroimaging data could enhance the precision and diagnostic utility of the model. Thirdly, the study identified emotional dysregulation and perceptual dysregulation subtypes, their practical application in clinical treatment remains unexplored. Fourth, while the individualized deviation maps and subtypes offer a promising path toward precision psychiatry, the modest effect sizes observed at the group level remind us that the immediate clinical translatability of these neuroimaging markers alone is currently limited. Future work should focus on integrating these neurophenotypes with multimodal data (e.g., genetics phenotyping) to build stronger predictive models for clinical outcomes. Finally, the study’s cross-sectional nature limits its ability to capture temporal dynamics of MDD progression. Longitudinal studies are needed to evaluate the stability of the normative model over time and to assess its applicability to other mental disorders, further exploring its universality and clinical utility.

## Conclusion

This study utilized a large, multi-site dataset to construct a normative model of ReHo, enabling the identification of deviations in patients with MDD and the classification of two distinct subtypes: emotional dysregulation and perceptual dysregulation. These subtypes exhibited unique neural abnormalities and clinical features, providing insight into disease severity and progression. Importantly, these subtypes map onto distinct levels of cortical hierarchical processing, offering a novel theoretical framework for understanding heterogeneity in MDD as dysregulation along the brain’s primary-transmodal functional gradient. The findings underscore the importance of personalized therapeutic approaches tailored to specific neurophenotypes, paving the way for precision medicine in MDD.

## Supplementary information

SUPPLEMENTARY APPENDIX

## Data Availability

The data that support the findings of this study are available from the corresponding author upon reasonable request.
