# Supplementary material for: Identifying neurophenotypes of major depressive disorder through normative model of regional homogeneity
Source: Transl Psychiatry. 2026 Apr 9;16:261. doi: 10.1038/s41398-026-04003-8 (PMC13184139; doi:10.1038/s41398-026-04003-8)
Supplement: Supplementary file 1 — SUPPLEMENTARY APPENDIX [file 41398_2026_4003_MOESM1_ESM.docx]

**SUPPLEMENTARY APPENDIX**

**Identifying Neurophenotypes of Major Depressive Disorder through Normative Models of Regional Homogeneity**

**SUPPLEMENTARY METHODS AND MATERIALS**

Our data statistics and visualization strategies were guided by the approach presented in SUN et al. [1], which provides a comprehensive framework for analyzing this type of data.

**Dataset Details**

In the REST-meta-MDD Project from the DIRECT consortium, 25 research groups from 17 hospitals in China agreed to share final R-fMRI indices from patients with major depressive disorder (MDD) and matched healthy controls (HCs) ([Table S1](https://kdocs.cn/l/ctRYanaaKUVL); henceforth “site” refers to each cohort for convenience) from studies approved by local Institutional Review Boards. The consortium contributed 2428 previously collected datasets (1,300 MDDs and 1,128 HCs). On average, each site contributed 52.0 ± 52.4 patients with MDD (range 13-282) and 45.1 ± 46.9 HCs (range 6-251). Age, sex, education years, episode status, medication status, and illness duration were collected for patients. The clinical symptoms of patients were assessed using the Hamilton Depression Rating Scale (HAMD) and Hamilton Anxiety Rating Scale (HAMA). Most patients with MDD were female (826 vs. 474 males), as expected. The 562 patients with first episode MDD included 318 first episode drug-naïve MDD and 160 scanned while receiving antidepressants (medication status unavailable for 84). Of 282 with recurrent MDD, 121 were scanned while receiving antidepressants and 76 were not being treated with medication (medication status unavailable for 85). Episodicity (first or recurrent) and medication status were unavailable for 456 patients.

**Sample Selection**

From the 1,300 patients with MDD and 1,128 HCs, we included subjects for the primary analyses using the following criteria, adapted from Yan et al. [2] ([Figure S1](F:\\1Normative_Model\\Extracted_Values\\ReHo_values\\All_Figure_Ai\\all_figure\\all_supplementary_figure.docx)): 1) Site 4 was excluded because its data was duplicated with Site 14, resulting in 1276 patients with MDD and 1104 HCs; 2) subjects without data on age and sex were excluded, resulting in 1276 patients with MDD and 1101 HCs; 3) subjects with poor image quality and spatial normalization (assessed by visual inspection) were excluded, resulting in 1162 patients with MDD and 1043 HCs; 4) subjects with excessive head motion (mean FD >0.2 mm) were excluded, resulting in 1137 patients with MDD and 1024 HCs; 5) subjects with abnormal regional spatial correlation (i.e., each subject’s regional homogeneity (ReHo) map was negatively correlated with the group mean ReHo map) were excluded, resulting in 1113 patients with MDD and 1023 HCs; 6) finally, we removed sites with fewer than 10 subjects in either group, resulting in 1101 patients with MDD and 1011 HCs. The age distribution of the participants is presented in [Figure S2](../All_Figure_Ai/all_figure/all_supplementary_figure.docx), while their demographic characteristics and clinical data are provided in [Table S2](../Table/TableS2_Demographic_characteristics_participants_included_primary_analysis.xlsx).

**Image Preprocessing**

First, the initial 10 volumes were excluded, followed by slice-timing correction. Next, each subject’s time series images underwent realignment through a six-parameter rigid-body linear transformation. After realignment, individual T1-weighted images were co-registered to the mean functional image using a six-degree-of-freedom linear transformation without resampling. Subsequently, these images were segmented into gray matter (GM), white matter (WM), and cerebrospinal fluid (CSF) [3]. Finally, transformations from the native space of each subject to MNI space were computed using the Diffeomorphic Anatomical Registration Through Exponentiated Lie Algebra (DARTEL) tool [4]. To mitigate head motion artifacts, we applied the Friston 24-parameter model [5] to regress out motion effects. Additionally, mean framewise displacement (FD), calculated via Jenkinson’s relative root mean square method [6], was included as a covariate in group analyses to further account for residual motion effects. For validation, scrubbing (removing time points where FD >0.2 mm) was employed to verify the results using a stringent head motion control approach. Since global signal regression (GSR) remains debated within the R-fMRI field [7], we did not perform GSR in primary analyses, but included GSR for validation analyses. Other sources of non-neuronal variance, such as signals from white matter (WM) and cerebrospinal fluid (CSF), were removed via linear regression to minimize respiratory and cardiac influences. A linear trend regressor was also included to address BOLD signal drifts. Temporal bandpass filtering (0.01–0.1 Hz) was applied to all time series.

After preprocessing, we calculated the ReHo map for each subject, which is a rank-based Kendall’s coefficient of concordance (KCC) that evaluates the synchronization of time courses among neighboring voxels, specifically considering the 27 nearest voxels in this analysis [8].

Prior to further analysis, all ReHo maps were Z-standardized by subtracting each voxel’s mean brain-wide value and dividing by its standard deviation, followed by smoothing with a 4 mm FWHM kernel.

**Harmonization of Site Effects**

After obtaining the ReHo maps for each HC, we extracted regional ReHo values for 246 brain areas using the Human Brainnetome Atlas, with 210 cortical and 36 subcortical subregions [9]. Following data extraction, regional ReHo values were harmonized across scanning sites using the ComBat empirical Bayes method [10, 11] to remove site-specific technical variance while preserving biological signals of interest. The ComBat model (neuroCombat python package) was specified with scanner site as the batch variable. To ensure the removal of pure technical noise, the following biological and clinical covariates were explicitly preserved (i.e., included in the model as adjustment variables): diagnosis (MDD vs. HC) and sex as categorical covariates, and age as well as mean FD as continuous covariates. This approach ensures that variance associated with diagnosis, demographic factors, and data quality is retained for subsequent analysis, while non-biological variance attributable to scanner differences is minimized.

**Normative Modeling for ReHo in HCs**

With the harmonized data, we used the PCNtoolkit package [12] to perform Gaussian process regression (GPR), modeling the normative trajectory of ReHo as a function of age and sex. GPR is a Bayesian nonlinear regression method that, by defining appropriate covariance (kernel) functions, generates probabilistic predictions based on observed data. A key advantage is its provision of both predicted mean values and confidence intervals, which quantify predictive uncertainty and can be interpreted as the normative range [13, 14].

The model was implemented using the toolkit’s default configuration, which employs a Radial Basis Function kernel. Hyperparameters (including length-scale and noise variance) were optimized separately for each of the 246 brain regions by maximizing the marginal likelihood during training, capturing region-specific trajectories. Age was included as a continuous linear covariate and sex as a binary categorical covariate, with no interaction term in the base model. To ensure numerical stability and comparability, regional ReHo values were Z-standardized prior to model fitting, while the age covariate was not standardized, following the toolkit’s standard configuration. This specific implementation follows the established normative modeling framework detailed in the toolkit’s documentation and related literature [1, 12–14].

To assess potential effects of age and sex on model construction, we re-estimated the normative model separately for male/female and young/old groups and conducted the subsequent analyses as the primary analyses (see Validation Analysis).

To rigorously evaluate the performance of the normative model, we employed 10-fold cross-validation, which is well-suited for assessing predictive accuracy while maximizing data utilization. The dataset was randomly partitioned into 10 equally sized subsets. In each fold, nine subsets were used to train the model, while the remaining one served as the test set, enabling independent evaluation. This process was repeated across all 10 folds, with each subset serving as the test set once, ensuring all data points contributed to both training and testing.

To assess model accuracy and reliability across all test folds, we computed the standardized mean squared error (SMSE) and mean standardized log loss (MSLL) metrics, which offer robust measures of prediction accuracy and uncertainty handling. SMSE evaluates the model’s prediction error relative to the natural variability in the data by comparing predicted values to true values in a standardized form, making it suitable for assessing performance across datasets with different scales. On the other hand, MSLL quantifies how well the model's probabilistic predictions align with actual outcomes, penalizing discrepancies based on the predicted uncertainty, thus providing insight into the model’s ability to capture variability in the data.

To identify patterns of age-related ReHo variation, a k-means clustering algorithm was performed within male and female groups. To assess the similarity between trajectories of different brain regions, a Pearson correlation matrix was calculated for each sex group and defined the distance between any two regions as one minus the Pearson correlation. This correlation-based distance metric effectively reflects trajectory similarity across brain regions. To determine the optimal number of clusters, we set the cluster number from 2 to 10 and ran the k-means clustering algorithm 10 times. We calculated the mean silhouette coefficient across runs to assess clustering performance, selecting the highest mean silhouette coefficient as the optimal cluster number for each sex group [15]. Using this optimal cluster number, we applied k-means clustering separately to male and female groups, assigning a cluster label to each brain region, thereby identifying ReHo variation patterns unique to each sex group.

**Estimating the ReHo Deviation from the Normative Model in Patients with MDD**

Using the normative model derived from the HC group, we input the age and sex of patients with MDD into this model to predict their expected ReHo values and corresponding confidence intervals. Next, we calculated the deviation by comparing the true ReHo values to the predicted means and normalize this difference as a deviation score (i.e., Z-deviation value) to quantify the extent of deviation from the normative model [1, 14]. For a specific patient with MDD i, the Z-deviation value of a brain region j was calculated as follows:

Where y_ij_ is the true ReHo value, ŷ is the predictive mean ReHo value, σ_ij_ is the predictive uncertainty, σ_nj_ is the variance derived from the normative distribution n, and the denominator is the total predictive standard deviation. The Z-deviation value quantifies how patients with MDD differ from the normative model. Specifically, the Z-deviation value represents the standardized extent to which the true values of patients with MDD diverge from the model predictive healthy norm, reflecting the degree of abnormality in ReHo for each brain region in patients with MDD. As a result, an individual deviation map was generated for each patient. To validate ReHo deviation calculations, the impact of patient sites was examined in the Validation Analysis. Likewise, for each HC participant, the individual deviation map was produced by calculating the Z-deviation value for each brain region during 10-fold cross-validation.

We defined the Z-deviation value exceeding ± 2.6 (equivalent to *p* < 0.005) as extreme deviation following established methodologies [1, 16]. We further measured inter-patient heterogeneity as well as between-group difference using the deviation indices, extreme deviation counts, spatial overlap maps and mean deviation maps.

These metrics are defined as follows: The overall deviation indices represent the average Z-deviation values across all brain regions for each subject and are used to measure the overall brain region deviation within the population. The positive deviation indices are the average Z-deviation values >0 across all brain regions for each subject and are used to assess the extent of positive deviation in brain regions within the population. Conversely, the negative deviation indices capture the average Z-deviation values <0. The overall extreme deviation counts indicate the number of brain regions where the Z-deviation value exceeds the extreme deviation threshold for each subject and are used to measure the extent of extreme deviations across brain regions. The positive extreme deviation counts reflect the number of brain regions with Z-deviation values >+2.6 and are used to assess the coverage of brain regions exhibiting positive extreme deviations. Conversely, the negative extreme deviation counts represent the number of brain regions with Z-deviation values <-2.6. The spatial overlap map is a brain map that shows the proportion of subjects whose deviation values in each brain region exceed the positive or negative extreme deviation thresholds. The mean deviation map is a brain map that displays the mean deviation level of all subjects in each brain region. This map is used to illustrate the overall deviation in each brain region and is employed in the analysis of group differences.

Group differences in deviation indices, extreme deviation counts, and mean deviation maps were tested using independent-sample t-tests between patients with MDD and HCs, with significance thresholds corrected for multiple comparisons via the Benjamini–Hochberg false discovery rate (FDR) procedure (FDR-corrected *p* < 0.05). Additionally, the impact of different thresholds for defining extreme deviation was assessed (see Validation Analysis).

**Identifying MDD Subtypes Based on ReHo Deviations**

To classify MDD subtypes, we applied a k-means clustering algorithm to deviation patterns, which is widely recognized in clustering analysis for its simplicity, computational efficiency, and applicability to large-scale datasets [17–19]. The optimal number of clusters was determined using the NbClust package, which applies over 30 clustering validity indices and a majority-vote mechanism to reduce subjective judgment and enhance the robustness of the clustering results [20]. Specifically, we used the deviation map of each patient as a clustering feature, defining the distance between patients as the Euclidean distance between their deviation maps. To reduce the impact of initial conditions, the clustering algorithm was repeated 10 times for each cluster count with different random initial centroids. We set cluster numbers from 2 to 10 and all available validity indices for a comprehensive quality assessment. Ultimately, the NbClust package recommended an optimal cluster number. For detailed information and documentation about the NbClust package, please visit [CRAN: Package NbClust](https://cran.r-project.org/web/packages/NbClust/).

To investigate the differences in imaging features between MDD subtypes, we calculated mean deviation maps for each subtype and compared them at the network level. Firstly, the Human Brainnetome Atlas has a total of 210 cortical subregions, which are assigned to the seven networks defined by Yeo et al. [21]: the fronto-parietal control network (FPCN), the ventral attention network (VAN), the default mode network (DMN), the dorsal attention network (DAN), the sensorimotor network (SMN), the limbic network (LN), and the visual network (VN). In addition, the atlas includes 36 subcortical subregions (SUB). The brain regions and the networks they belong to are available at <https://atlas.brainnetome.org/>. We then classified the brain regions using each network label and calculated the mean Z-deviation values at the network level. Finally, independent-sample t-tests were performed on the mean values calculated for each network to assess significant differences between subtypes at the network level.

We used deviation indices and extreme deviation counts to measure the degree of deviation in MDD subtypes. One-way analysis of variance was conducted to compare the deviation indices and extreme deviation counts among subtypes and healthy controls (HC), with post hoc independent-sample t-tests for group comparisons. To assess differences in brain region vulnerability and sensitivity across MDD subtypes and the entire patient population, spatial overlap maps of extreme positive and negative deviations were computed for each subtype. Independent-sample t-tests were then used to compare these maps between subtypes and all patients.

We compared demographic and clinical variables between subtypes, including age, sex, education years, episode status, medication status, illness duration, and scores on the Hamilton Anxiety Rating Scale-14 item (HAMA-14) and Hamilton Depression Rating Scale-17 item (HAMD-17) using independent-sample t-tests or chi-square tests. To further characterize the clinical features of the identified subtypes, we compared their scores on specific symptom dimensions of the HAMD-17. The total HAMD score was decomposed into five symptom factor: anxiety/somatization, weight, cognitive impairment, retardation, and sleep disturbance [22]. Each participant’s factor score was calculated by summing the scores of the constituent items: anxiety/somatization (items 10, 11, 12, 13, 15, 17), weight (item 16), cognitive impairment (items 2, 3, 9), retardation (items 1, 7, 8, 14), sleep disturbance (items 4, 5, 6). To examine the correlation between the HAMD-17 total score and illness duration for each subtype, we conducted a one-way analysis of covariance with illness duration as the independent variable, HAMD-17 total score as the dependent variable, and subtype as the categorical factor. Post hoc analyses included Pearson’s correlation coefficients between HAMD-17 total score and illness duration for each subtype.

**Validation Analysis**

**Site Effects on Results**

To investigate the effect of patient sites on ReHo deviation calculations, we used one-way ANOVA to compare Z-deviation values among sites for each brain region, with FDR-corrected *p* < 0.05 for multiple comparisons. To assess the robustness of the subtyping results, we calculated the mean Euclidean distance between the subtype percentages of each site and the overall population. We also performed k-means clustering at each site and calculated the mean Euclidean distance between the site-specific subtype percentages and those of the overall population. A leave-one-site-out validation was conducted by excluding one site at a time and calculating the overlap rate between clustering labels each time and those from the primary results. We repeated this process 22 times to ensure all sites were excluded once.

Following Dalmaijer and colleagues’ recommendation of a minimum sample size of 20-30 per subtype to enhance statistical power, especially when dealing with a limited number of clusters [23], we excluded sites with fewer than 30 patients and proceeded with additional clustering and validation analyses. K-means clustering was performed using the NbClust package. After subtyping, we recalculated the mean Euclidean distance between the subtype percentages at each site and the percentages of overall population. Additionally, we performed k-means clustering for each site and reassessed the mean Euclidean distance between the site-specific subtype percentages and the overall population. Finally, we conducted the leave-one-site-out validation to assess the influence of specific sites.

**Different Threshold in the Extreme Deviation Definition**

In the primary analysis, we defined Z = ± 2.6 as the threshold for extreme deviation values. For improved reliability, we FDR-corrected Z-deviation values in the Validation Analysis, setting the threshold for extreme deviations at *p* < 0.05. Two-tailed *p*-values were calculated from Z-deviation values for each subject based on the standard normal distribution, FDR-corrected for each brain region, and a separate threshold was determined for each subject. We then re-evaluated deviation indices, extreme deviation counts (overall, positive, and negative), and spatial overlap maps, comparing them with HCs. After clustering patients into subtypes based on deviation maps, we used one-way ANOVA to compare extreme deviation counts among subtypes and HCs. Due to limited number of surviving brain regions after multiple comparisons, deviation indices were not compared further. Spatial overlap maps of each subtype were compared with the entire patient group using independent-sample t-tests.

**Age and Sex Effects on Results**

To assess the effects of age and sex on model construction, we estimated normative models separately for young (<21 years) and old (≥21 years) groups, as well as for females and males. We reanalyzed the heterogeneity of MDD subtypes within each group. The young and old groups were defined using the commonly applied age threshold from previous studies that differentiates between early-onset and late-onset patients [24, 25].

Normative models were reconstructed for each brain region in HCs, and ReHo variation trajectories were identified using k-means clustering. The overlap rate between these clustering labels and the primary results was calculated. Individual deviation patterns for patients with MDD were derived using the normative model. To assess intersubject heterogeneity, we analyzed the number of regions with extreme deviations for each patient and generated spatial overlap maps to identify shared extreme deviations and vulnerable brain regions. The mean deviation map for patients was compared to the primary results using Pearson’s correlation.

Subsequently, MDD subtypes were re-clustered based on individual deviation patterns, and the overlap rate with the primary results was assessed. Mean deviation maps for each subtype were compared to the primary findings using Pearson’s correlation, and spatial overlap maps for each subtype were contrasted with the primary results.

**Clinical Effects on Results**

To evaluate the stability of the identified neurophenotypes across key clinical presentations of MDD, we conducted validation analyses within key clinical subgroups: first‑episode drug‑naïve (FEDN) patients, recurrent patients, and medicated patients. Given that these subgroups are defined solely within the patient cohort, no separate normative models were reconstructed for healthy controls. Instead, we extracted the pre‑computed individual ReHo deviation maps (Z‑scores) for patients belonging to each subgroup. Within each subgroup, we reapplied the same k‑means clustering procedure to the deviation patterns and calculated the overlap rate between these clustering labels and the primary results. We further assessed the spatial similarity of mean deviation maps for each subtype across subgroups via Pearson correlation, and generated subgroup-specific spatial overlap maps to examine the consistency of vulnerable brain regions.

**Algorithm Effects on Results**

To verify the robustness of the primary k-means clustering, we additionally applied Gaussian mixture models (GMM), hierarchical clustering and spectral clustering to the same individual deviation maps. The consistency between different algorithmic solutions was quantified using the label overlap and adjusted rand index (ARI).

**Head Motion Effects on Results**

A significant difference in FD was observed between the two identified MDD subtypes (albeit with a small mean difference of 0.01, *p* < 0.001). Therefore, we performed a validation analysis to explicitly control for head motion effects within the normative modeling framework. A new normative model was constructed using the HCs (n = 1,011), with age, sex, and mean FD included as covariates in the GPR. This model was then applied to derive individual deviation maps for the patients with MDD (n = 1,101). The k-means clustering algorithm was reapplied to these FD-adjusted deviation maps, with the optimal number of clusters determined using the NbClust package. The stability of the subtype solution was quantified by calculating the overlap rate and the ARI between the clustering labels from this model and those from the primary results.

**GSR Effects on Results**

To assess the effect of global signal removal, we reprocessed the data with an additional GSR step. All subsequent preprocessing steps and analyses were identical to the primary pipeline: ReHo was calculated within the Human Brainnetome Atlas, a normative model was built on HCs (n = 1,011), and individual deviation maps were generated for patients with MDD (n = 1,101). Subtypes were identified via k-means clustering on these GSR-processed deviation maps, with the optimal number of clusters determined using the NbClust package. Subtype stability was again assessed via overlap rate and ARI against the primary results.

**Brain Parcellation Effects on Results**

To evaluate the reproducibility of our findings across different brain parcellation schemes, we repeated the core analysis using the functionally-defined Dosenbach-160 atlas [26]. This atlas, specifically designed for rs-fMRI analysis, maintains consistency with our primary atlas by also omitting cerebellar regions. Following identical preprocessing, ReHo values were extracted for its 142 regions. A normative model was built from HCs (n = 1,011), individual deviation maps were computed for patients with MDD (n = 1,101), and k-means clustering was performed, with the optimal number of clusters determined using the NbClust package. The agreement between the subtypes derived from this atlas and those from the primary Brainnetome-246 analysis was quantified using both the overlap rate and the ARI.

**SUPPLEMENTARY RESULTS**

**Validation Results**

**Site Effects on Results**

We assessed site effects on ReHo deviation using one-way ANOVA to compare Z-deviation values across sites for each brain region. No significant site effects were observed across any of the 246 brain regions (FDR-corrected *p* = 0.108-1.000. To evaluate subtyping robustness, we calculated the mean Euclidean distance between site-specific subtype percentages and the overall population percentages (Euclidean distance = 0.12 ± 0.11) ([Table S4](F:\\1Normative_Model\\Extracted_Values\\ReHo_values\\Table\\TableS4_subtype_site_proportion.xlsx)). K-means clustering at each site showed a similar result (Euclidean distance = 0.11 ± 0.08) ([Table S5](../Table/TableS5_k_means_clustering_proportion.xlsx)), and leave-one-site-out validation revealed >98% overlap between new clustering labels and the primary results ([Figure S7](../All_Figure_Ai/all_figure/all_supplementary_figure.docx)).

After excluding sites with fewer than 30 subjects, we repeated the one-way ANOVA and found significant differences only in four thalamus subregions (FDR-corrected *p* = 0.021-0.998), suggesting minimal site effects. K-means clustering using the NbClust package identified two MDD subtypes: subtype 1 (37%, n = 330) and subtype 2 (63%, n = 570) ([Figure S5A](../All_Figure_Ai/all_figure/all_supplementary_figure.docx)), showing 98.89% overlap with the primary results. The mean Euclidean distance between site-specific subtype percentages and the overall population was 0.08 ± 0.08 ([Table S6](../Table/TableS6_subtype_site_proportion(above_30_patients)_percentages.xlsx)), and k-means clustering at each site produced a mean Euclidean distance of 0.09 ± 0.06 ([Table S7](../Table/TableS7_k_means_table_every_site(above_30_patients)_percentages.xlsx)). Leave-one-site-out validation showed >94% overlap with the primary results ([Figure S8](../All_Figure_Ai/all_figure/all_supplementary_figure.docx)).

**Different Thresholds in the Extreme Deviation Definition**

Compared with HCs, neither the three deviation indices nor the three extreme deviation counts showed significant differences in patients with MDD. At the individual level, 9.63% (n = 106) of patients with MDD had at least one brain region with extreme deviation from the normative model. Among these, 8.63% (n = 95) showed extreme positive deviations, while 1.36% (n = 15) had extreme negative deviations ([Figure S9A](../All_Figure_Ai/all_figure/all_supplementary_figure.docx)). Across all brain regions, 40.65% (n = 100) of the nodes showed extreme deviations in every patient (extreme positive: 36.59%, n = 90; extreme negative: 7.32%, n = 18). Positive deviations were most prevalent in the prefrontal cortex and hippocampal gyrus, while negative deviations were concentrated in sensorimotor areas ([Figure S9B](../All_Figure_Ai/all_figure/all_supplementary_figure.docx)). The percentage of patients with extreme deviations from the normative model was very low for both positive (≤ 0.81%) and negative (≤ 0.45%) deviations.

Statistical comparisons also showed no significant differences in extreme deviation counts among MDD subtypes and HCs. Spatial overlap maps of extreme deviations revealed that the vulnerability of brain regions was lower in each subtype than all in patients with MDD, likely due to the limited number of regions surviving correction ([Figure S9C](../All_Figure_Ai/all_figure/all_supplementary_figure.docx)). Even with a stricter definition of extreme deviation, regional vulnerability differed between subtypes, suggesting subtype-specific patterns. For example, the emotional dysregulation subtype showed extreme deviations in the prefrontal cortex and hippocampal gyrus, which regulate emotion and cognition [27, 28], while the perceptual dysregulation subtype had extreme deviations in sensorimotor areas related to bodily sensation and motor control [29]. These regions may be key targets for damage in these MDD subtypes and warrant further investigation.

**Age and Sex effects on Results**

**Male Group**

We constructed separate normative models for the male group and age-related ReHo variation patterns closely mirrored the primary results. Two distinct patterns of age-related ReHo variation were identified, with a 95.93% overlap rate between the new cluster labels and the primary results ([Figure S10A](../All_Figure_Ai/all_figure/all_supplementary_figure.docx)). The mean deviation map for patients showed strong similarity to the primary results (*r* = 0.83, *p* < 0.001, [Figure S10B](../All_Figure_Ai/all_figure/all_supplementary_figure.docx)). At the individual level, 79.34% (n = 311) of patients with MDD had at least one brain region with extreme deviation from the normative model. Among these, 73.72% (n = 289) showed extreme positive deviations, while 29.08% (n = 114) had extreme negative deviations ([Figure S10C](../All_Figure_Ai/all_figure/all_supplementary_figure.docx)). Across all brain regions (n = 246), 97.15% (n = 239) of nodes showed extreme deviations in every patient (extreme positive: 95.93%, n = 236; extreme negative: 52.44%, n = 129). Positive and negative deviations were consistent with the primary results (positive: *p* = 0.065; negative: *p* = 0.079). The percentage of patients with extreme deviations from the normative model remained very low for both positive (≤ 3.31%, n = 13) and negative (≤ 1.53%, n = 6) deviations ([Figure S10C](../All_Figure_Ai/all_figure/all_supplementary_figure.docx)).

Using the k-means clustering algorithm, we identified two distinct MDD subtypes based on individual ReHo deviations. Subtype 1 comprised 36% (n = 141) of patients, while subtype 2 represented 64% (n = 251) ([Figure S10D](../All_Figure_Ai/all_figure/all_supplementary_figure.docx)). The overlap between the clustered labels from this approach and those from the primary results was 97.45%, corresponding to an ARI of 0.90. At the network level, subtype 1 showed positive deviation in the DAN, SMN, and VN, and negative deviation in the FPCN, VAN, DMN, LN, and SUB, and vice versa for subtype 2 (absolute *d* = 0.22-2.06, FDR-corrected *p* < 0.05, [Figure S10E](../All_Figure_Ai/all_figure/all_supplementary_figure.docx)). The mean deviation maps for both subtypes were highly similar to those in the primary results (subtype 1: *r* = 0.98, *p* < 0.001; subtype 2: *r* = 0.98, *p* < 0.001, Figure S10 E).

Spatial overlap maps highlighted that both subtype 1 (positive: 0.71%-5.67%, *d* = 0.04, FDR-corrected *p* = 0.668; negative: 0.71%-2.83%, *d* = 0.37, FDR-corrected *p* < 0.05) and subtype 2 (positive: 0.40%-5.18%, *d* = 0.20, FDR-corrected *p* < 0.05; negative: 0.40%-1.99%, *d* = 0.08, FDR-corrected *p* = 0.380) exhibited higher vulnerability than all patients with MDD ([Figure S10F](../All_Figure_Ai/all_figure/all_supplementary_figure.docx)).

**Female Group**

We constructed separate normative models for the female group, and age-related ReHo variation patterns closely mirrored the primary results. Two distinct patterns of age-related ReHo variation were identified, with a 96.75% overlap rate between the new cluster labels and the primary results ([Figure S11A](../All_Figure_Ai/all_figure/all_supplementary_figure.docx)). The mean deviation map for patients showed strong similarity to the primary results (*r* = 0.95, *p* < 0.001, [Figure S11B](../All_Figure_Ai/all_figure/all_supplementary_figure.docx)). At the individual level, 77.15% (n = 547) of patients with MDD had at least one brain region with extreme deviation from the normative model. Among these, 72.64% (n = 515) showed extreme positive deviations, while 24.40% (n = 173) had extreme negative deviations ([Figure S11C](../All_Figure_Ai/all_figure/all_supplementary_figure.docx)). Across all brain regions (n = 246), at least one extreme deviation was present in every patient (extreme positive: 100%; extreme negative: 63.82%, n = 157). Positive and negative deviations were consistent with the primary results (positive: *p* = 0.943; negative: *p* = 0.432). The percentage of patients with extreme deviations from the normative model remained very low for both positive (≤ 3.80%, n = 27) and negative (≤ 1.13%, n = 8) deviations ([Figure S11C](../All_Figure_Ai/all_figure/all_supplementary_figure.docx)).

Using the k-means clustering algorithm, we identified two distinct MDD subtypes based on individual ReHo deviations. Subtype 1 comprised 37% (n = 260) of patients, while subtype 2 represented 63% (n = 449) ([Figure S11D](../All_Figure_Ai/all_figure/all_supplementary_figure.docx)). The overlap between the clustered labels from this approach and those from the primary results was 98.87%, corresponding to an ARI of 0.96. At the network level, subtype 1 showed positive deviation in the DAN, SMN, and VN, and negative deviation in the FPCN, VAN, DMN, and LN, and vice versa for subtype 2 (absolute *d* = 0.41-1.91, FDR-corrected *p* < 0.05, [Figure S11E](../All_Figure_Ai/all_figure/all_supplementary_figure.docx)). The mean deviation maps for both subtypes were highly similar to those in the primary results (subtype 1: *r* = 0.99, *p* < 0.001; subtype 2: *r* = 0.99, *p* < 0.001, [Figure S11E](../All_Figure_Ai/all_figure/all_supplementary_figure.docx)).

Spatial overlap maps highlighted that subtype 1 exhibited greater disease impact, with higher rates of extreme deviations than in all patients with MDD (positive: 0.38%-4.62%, *d* = 0.18, FDR-corrected *p* < 0.05; negative: 0.38%-3.08%, *d* = 0.19, FDR-corrected *p* < 0.05). In contrast, subtype 2 demonstrated lower sensitivity (positive: 0.22%-3.79%, *d* = 0.14, FDR-corrected *p* = 0.130; negative: 0.22%-1.34%, *d* = -0.27, FDR-corrected *p* < 0.05) ([Figure S11F](../All_Figure_Ai/all_figure/all_supplementary_figure.docx)).

**Young Group**

We categorized subjects into young (HCs: n = 102, MDD: n = 154) and old (HCs: n = 909, MDD: n = 947) groups based on age. For the normative models constructed on these groups, we combined the age-related ReHo variation trajectories of brain regions. We found that these combined trajectories closely resembled those observed in the primary results. Two distinct patterns of age-related ReHo variation were identified, with a 99% (99.59% for males, 99.19% for females, [Figure S12A](../All_Figure_Ai/all_figure/all_supplementary_figure.docx)) overlap rate between the new cluster labels and the primary results. In the young group, the mean deviation map for patients showed some similarity to the primary results (*r* = 0.23, *p* < 0.001, [Figure S12B](../All_Figure_Ai/all_figure/all_supplementary_figure.docx)). At the individual level, 75.97% (n = 117) of patients with MDD had at least one brain region with extreme deviation from the normative model. Among these, 68.83% (n = 106) showed extreme positive deviations, while 30.52% (n = 47) had extreme negative deviations ([Figure S12C](../All_Figure_Ai/all_figure/all_supplementary_figure.docx)). Across all brain regions, 80.49% (n = 198) of the nodes showed extreme deviations in every patient (extreme positive: 71.14%, n = 175; extreme negative: 30.49%, n = 75). Positive and negative deviations were partially consistent with the primary results (positive: *p* = 0.478; negative: *p* = 0.025). The percentage of patients with extreme deviations from the normative model remained very low for both positive (≤ 4.55%, n = 7) and negative (≤ 2.60%, n = 4) deviations ([Figure S12C](../All_Figure_Ai/all_figure/all_supplementary_figure.docx)).

Using the k-means clustering algorithm, we identified two distinct MDD subtypes based on individual ReHo deviations. Subtype 1 comprised 52% (n = 80) of patients, while subtype 2 represented 48% (n = 74) ([Figure S12D](../All_Figure_Ai/all_figure/all_supplementary_figure.docx)). The overlap between the clustered labels from this approach and those from the primary results was 89.61%, corresponding to an ARI of 0.63. At the network level, subtype 1 showed positive deviation in the DAN, SMN, VN, and SUB and negative deviation in the FPCN, DMN, and LN and vice versa for subtype 2 (absolute *d* = 0.37-1.91, FDR-corrected *p* < 0.05, [Figure S12E](../All_Figure_Ai/all_figure/all_supplementary_figure.docx)). The mean deviation maps for both subtypes were highly similar to those in the primary results (subtype 1: *r* = 0.84, *p* < 0.001; subtype 2: *r* = 0.82, *p* < 0.001, [Figure S12E](../All_Figure_Ai/all_figure/all_supplementary_figure.docx)).

Spatial overlap maps highlighted that subtype 1 exhibited similar sensitivity levels to those seen in all patients with MDD (positive: 1.25%-6.25%, *d* = 0.04, FDR-corrected *p* = 0.629; negative: 1.25%-3.75%, *d* = 0.10, FDR-corrected *p* = 0.253). In contrast, subtype 2 demonstrated greater disease impact, with higher rates of extreme deviations (positive: 1.35%-5.41%, *d* = 0.05, FDR-corrected *p* = 0.591; negative: 1.35%-4.05%, *d* = 0.22, FDR-corrected *p* < 0.05) ([Figure S12F](../All_Figure_Ai/all_figure/all_supplementary_figure.docx)).

**Old Group**

In the old group, the mean deviation map for the patients showed strong similarity to the primary results (*r* = 0.98, *p* < 0.001, [Figure S13A](../All_Figure_Ai/all_figure/all_supplementary_figure.docx)). At the individual level, 76.87% (n = 728) of patients with MDD had at least one brain region with extreme deviation from the normative model. Among these, 72.86% (n = 690) showed extreme positive deviations, while 25.45% (n = 241) had extreme negative deviations ([Figure S13B](../All_Figure_Ai/all_figure/all_supplementary_figure.docx)). Across all brain regions (n = 246), at least one extreme deviation was present in every patient (extreme positive: 100%; extreme negative: 73.98%, n = 182). Positive and negative deviations were consistent with the primary results (positive: *p* = 0.473; negative: *p* = 0.809). The percentage of patients with extreme deviations from the normative model remained very low for both positive (≤ 2.75%, n = 26) and negative (≤ 1.16%, n = 11) deviations ([Figure S13B](../All_Figure_Ai/all_figure/all_supplementary_figure.docx)).

Using the k-means clustering algorithm, we identified two distinct MDD subtypes based on individual ReHo deviations. Subtype 1 comprised 34% (n = 320) of patients, while subtype 2 represented 66% (n = 627) ([Figure S13C](../All_Figure_Ai/all_figure/all_supplementary_figure.docx)). The overlap between the clustered labels from this approach and those from the primary results was 97.99%, corresponding to an ARI of 0.92. At the network level, subtype 1 showed positive deviation in the DAN, SMN, and VN and negative deviation in the FPCN, VAN, DMN, LN, and SUB and vice versa for subtype 2 (absolute *d* = 0.23-1.93, FDR-corrected *p* < 0.05, [Figure S13D](../All_Figure_Ai/all_figure/all_supplementary_figure.docx)). The mean deviation maps for both subtypes were highly similar to those in the primary results (subtype 1: *r* = 0.99, *p* < 0.001; subtype 2: *r* = 0.99, *p* < 0.001, [Figure S13D](../All_Figure_Ai/all_figure/all_supplementary_figure.docx)).

Spatial overlap maps highlighted that subtype 1 exhibited greater disease impact, with higher rates of extreme deviations than all patients with MDD (positive: 0.31%-4.06%, *d* = 0.13, FDR-corrected *p* = 0.160; negative: 0.31%-2.81%, *d* = 0.36, FDR-corrected *p* < 0.05). In contrast, subtype 2 demonstrated lower sensitivity (positive: 0.16%-3.83%, *d* = 0.01, FDR-corrected *p* = 0.936; negative: 0.16%-1.28%, *d* = -0.33, FDR-corrected *p* < 0.001) ([Figure S12E](../All_Figure_Ai/all_figure/all_supplementary_figure.docx)).

**Clinical effects on Results**

**First-episode drug-naïve Group**

In the FEDN group (n = 286), the mean deviation map for the patients showed strong similarity to the primary results (*r* = 0.83, *p* < 0.001, [Figure S14A](../All_Figure_Ai/all_figure/all_supplementary_figure.docx)). At the individual level, 73.43% (n = 210) of patients with MDD had at least one brain region with extreme deviation from the normative model. Among these, 68.18% (n = 195) showed extreme positive deviations, while 24.83% (n = 71) had extreme negative deviations ([Figure S14B](../All_Figure_Ai/all_figure/all_supplementary_figure.docx)). Across all brain regions, 96.34% (n = 237) of the nodes showed extreme deviations in every patient (extreme positive: 93.90%, n = 231; extreme negative: 48.78%, n = 120). Positive and negative deviations were consistent with the primary results (positive: *p* = 0.225; negative: *p* = 0.243). The percentage of patients with extreme deviations from the normative model remained very low for both positive (≤ 3.15%, n = 9) and negative (≤ 1.40%, n = 4) deviations ([Figure S14B](../All_Figure_Ai/all_figure/all_supplementary_figure.docx)).

Using the k-means clustering algorithm, we identified two distinct MDD subtypes based on individual ReHo deviations. Subtype 1 comprised 34% (n = 97) of patients, while subtype 2 represented 66% (n = 189) ([Figure S14C](../All_Figure_Ai/all_figure/all_supplementary_figure.docx)). The overlap between the clustered labels from this approach and those from the primary results was 95.45%, corresponding to an ARI of 0.82. At the network level, subtype 1 showed positive deviation in the DAN, SMN, and VN and negative deviation in the FPCN, DMN, and LN and vice versa for subtype 2 (absolute *d* = 0.53-2.29, FDR-corrected *p* < 0.05, [Figure S14D](../All_Figure_Ai/all_figure/all_supplementary_figure.docx)). The mean deviation maps for both subtypes were highly similar to those in the primary results (subtype 1: *r* = 0.96, *p* < 0.001; subtype 2: *r* = 0.96, *p* < 0.001, [Figure S14D](../All_Figure_Ai/all_figure/all_supplementary_figure.docx)).

Spatial overlap maps highlighted that subtype 1 exhibited greater disease impact, with higher rates of extreme deviations than all patients with MDD (positive: 0.01%-6.19%, *d* = -0.18, FDR-corrected *p* < 0.05; negative: 0.01%-4.23%, *d* = -0.27, FDR-corrected *p* < 0.05). In contrast, subtype 2 demonstrated lower sensitivity (positive: 0.01%-4.12%, *d* = 0.28, FDR-corrected *p* < 0.05; negative: 0.01%-1.59%, *d* = 0.24, FDR-corrected *p* < 0.05) ([Figure S14E](../All_Figure_Ai/all_figure/all_supplementary_figure.docx)).

**Recurrent Group**

In the recurrent group (n = 233), the mean deviation map for the patients showed strong similarity to the primary results (*r* = 0.85, *p* < 0.001, [Figure S15A](../All_Figure_Ai/all_figure/all_supplementary_figure.docx)). At the individual level, 78.54% (n = 183) of patients with MDD had at least one brain region with extreme deviation from the normative model. Among these, 74.68% (n = 174) showed extreme positive deviations, while 22.32% (n = 52) had extreme negative deviations ([Figure S15B](../All_Figure_Ai/all_figure/all_supplementary_figure.docx)). Across all brain regions, 90.24% (n = 222) of the nodes showed extreme deviations in every patient (extreme positive: 87.40%, n = 215; extreme negative: 48.78%, n = 120). Positive and negative deviations were partially consistent with the primary results (positive: *p* = 0.287; negative: *p* = 0.003). The percentage of patients with extreme deviations from the normative model remained very low for both positive (≤ 3.43%, n = 8) and negative (≤ 1.72%, n = 4) deviations ([Figure S15B](../All_Figure_Ai/all_figure/all_supplementary_figure.docx)).

Using the k-means clustering algorithm, we identified two distinct MDD subtypes based on individual ReHo deviations. Subtype 1 comprised 48% (n = 111) of patients, while subtype 2 represented 52% (n = 122) ([Figure S15C](../All_Figure_Ai/all_figure/all_supplementary_figure.docx)). The overlap between the clustered labels from this approach and those from the primary results was 72.53%, corresponding to an ARI of 0.20. At the network level, subtype 1 showed positive deviation in the VAN, SMN, LN, VN and SUB and negative deviation in the FPCN and DMN and vice versa for subtype 2 (absolute *d* = 0.28-1.60, FDR-corrected *p* < 0.05, [Figure S15D](../All_Figure_Ai/all_figure/all_supplementary_figure.docx)). The mean deviation maps for both subtypes were highly similar to those in the primary results (subtype 1: *r* = 0.44, *p* < 0.001; subtype 2: *r* = 0.70, *p* < 0.001, [Figure S15D](../All_Figure_Ai/all_figure/all_supplementary_figure.docx)).

Spatial overlap maps highlighted that subtype 1 exhibited greater disease impact, with higher rates of extreme deviations than all patients with MDD (positive: 0.01%-5.41%, *d* = -0.22, FDR-corrected *p* < 0.05; negative: 0.01%-1.80%, *d* = 0.23, FDR-corrected *p* < 0.05). In contrast, subtype 2 demonstrated lower sensitivity (positive: 0.01%-5.74%, *d* = 0.11, FDR-corrected *p* = 0.230; negative: 0.01%-1.64%, *d* = 0.21, FDR-corrected *p* < 0.05) ([Figure S15E](../All_Figure_Ai/all_figure/all_supplementary_figure.docx)).

**Medicated Group**

In the medicated group (n = 341), the mean deviation map for the patients showed strong similarity to the primary results (*r* = 0.91, *p* < 0.001, [Figure S16A](../All_Figure_Ai/all_figure/all_supplementary_figure.docx)). At the individual level, 78.89% (n = 269) of patients with MDD had at least one brain region with extreme deviation from the normative model. Among these, 73.61% (n = 251) showed extreme positive deviations, while 26.10% (n = 89) had extreme negative deviations ([Figure S16B](../All_Figure_Ai/all_figure/all_supplementary_figure.docx)). Across all brain regions, 97.15% (n = 239) of the nodes showed extreme deviations in every patient (extreme positive: 95.53%, n = 235; extreme negative: 45.53%, n = 112). Positive and negative deviations were consistent with the primary results (positive: *p* = 0.927; negative: *p* = 0.530). The percentage of patients with extreme deviations from the normative model remained very low for both positive (≤ 3.52%, n = 12) and negative (≤ 1.47%, n = 5) deviations ([Figure S16B](../All_Figure_Ai/all_figure/all_supplementary_figure.docx)).

Using the k-means clustering algorithm, we identified two distinct MDD subtypes based on individual ReHo deviations. Subtype 1 comprised 31% (n = 106) of patients, while subtype 2 represented 69% (n = 235) ([Figure S16C](../All_Figure_Ai/all_figure/all_supplementary_figure.docx)). The overlap between the clustered labels from this approach and those from the primary results was 94.72%, corresponding to an ARI of 0.79. At the network level, subtype 1 showed positive deviation in the DAN, SMN, and VN and negative deviation in the FPCN, VAN, DMN, LN, and SUB and vice versa for subtype 2 (absolute *d* = 0.52-2.00, FDR-corrected *p* < 0.05, [Figure S16D](../All_Figure_Ai/all_figure/all_supplementary_figure.docx)). The mean deviation maps for both subtypes were highly similar to those in the primary results (subtype 1: *r* = 0.90, *p* < 0.001; subtype 2: *r* = 0.98, *p* < 0.001, [Figure S16D](../All_Figure_Ai/all_figure/all_supplementary_figure.docx)).

Spatial overlap maps highlighted that subtype 1 exhibited greater disease impact, with higher rates of extreme deviations than all patients with MDD (positive: 0.01%-6.60%, *d* = 0.06, FDR-corrected *p* = 0.481; negative: 0.01%-2.83%, *d* = -0.27, FDR-corrected *p* < 0.05). In contrast, subtype 2 demonstrated lower sensitivity (positive: 0.004%-3.83%, *d* = -0.03, FDR-corrected *p* = 0.740; negative: 0.004%-1.28%, *d* = 0.23, FDR-corrected *p* < 0.05) ([Figure S16E](../All_Figure_Ai/all_figure/all_supplementary_figure.docx)).

**Algorithm Effects on Results**

To assess the stability of the clustering results, we applied GMM, hierarchical clustering and spectral clustering to the same deviation maps. The two-subtype solution showed high stability between K-means and GMM (label overlap = 0.98, ARI = 0.93), and hierarchical clustering (label overlap = 0.88, ARI = 0.58). In contrast, spectral clustering yielded a lower concordance (label overlap = 0.65, ARI = 0.13) (Table S15).

**Head Motion Effects on Results**

When mean FD was included as a covariate in the normative model, the optimal cluster number for the MDD group remained two, as determined by the majority vote of the NbClust indices (Figure S17A). Subtype 1 comprised 36% (n = 401) of patients, and Subtype 2 comprised 64% (n = 700). Assessment of inter-patient similarity using Euclidean distance confirmed considerable homogeneity within each subtype and high heterogeneity between them (Figure S17A). The clustering labels derived from this FD-adjusted model showed a 93.19% overlap rate with the labels from the primary results, corresponding to an ARI of 0.74.

**GSR Effects on Results**

Reprocessing the data with GSR did not alter the optimal two-cluster solution (Figure S17B). The subtype distribution was highly similar to the primary result, with Subtype 1 including 35% (n = 387) and Subtype 2 including 65% (n = 714) of patients. Euclidean distance metrics again indicated strong within-subtype similarity and clear separation between subtypes (Figure S17B). The agreement between the clustering solution obtained with GSR and the primary solution was substantial, with an overlap rate of 90.64% and an ARI of 0.66.

**Brain Parcellation Effects on Results**

Replicating the entire normative modeling and clustering pipeline using the Dosenbach atlas yielded a consistent two-subtype structure (Figure S17C). The resulting subtype proportions were nearly identical to the primary results (Subtype 1: 36%, n = 399; Subtype 2: 64%, n = 702), with clear separation evidenced by Euclidean distance (Figure S17C). The overlap rate between the subtypes defined by this alternative atlas and our primary Brainnetome-246 based was 87.74%, corresponding to an ARI of 0.57.

**SUPPLEMENTARY REFERENCES**

1. Sun X, Sun J, Lu X, Dong Q, Zhang L, Wang W, et al. Mapping Neurophysiological Subtypes of Major Depressive Disorder Using Normative Models of the Functional Connectome. Biol Psychiatry. 2023;94:936–947.

2. Yan C-G, Chen X, Li L, Castellanos FX, Bai T-J, Bo Q-J, et al. Reduced default mode network functional connectivity in patients with recurrent major depressive disorder. Proc Natl Acad Sci. 2019;116:9078–9083.

3. Ashburner J, Friston KJ. Unified segmentation. NeuroImage. 2005;26:839–851.

4. Ashburner J. A fast diffeomorphic image registration algorithm. NeuroImage. 2007;38:95–113.

5. Friston KJ, Williams S, Howard R, Frackowiak RSJ, Turner R. Movement‐Related effects in fMRI time‐series. Magn Reson Med. 1996;35:346–355.

6. Jenkinson M, Bannister P, Brady M, Smith S. Improved optimization for the robust and accurate linear registration and motion correction of brain images. NeuroImage. 2002;17:825–841.

7. Murphy K, Fox MD. Towards a consensus regarding global signal regression for resting state functional connectivity MRI. NeuroImage. 2017;154:169–173.

8. Zang Y, Jiang T, Lu Y, He Y, Tian L. Regional homogeneity approach to fMRI data analysis. NeuroImage. 2004;22:394–400.

9. Fan L, Li H, Zhuo J, Zhang Y, Wang J, Chen L, et al. The Human Brainnetome Atlas: A New Brain Atlas Based on Connectional Architecture. Cereb Cortex N Y N 1991. 2016;26:3508–3526.

10. Harmonization of multi-site diffusion tensor imaging data. NeuroImage. 2017;161:149–170.

11. Fortin J-P, Cullen N, Sheline YI, Taylor WD, Aselcioglu I, Cook PA, et al. Harmonization of cortical thickness measurements across scanners and sites. NeuroImage. 2018;167:104–120.

12. Rutherford S, Kia SM, Wolfers T, Fraza C, Zabihi M, Dinga R, et al. The normative modeling framework for computational psychiatry. Nat Protoc. 2022;17:1711–1734.

13. Rasmussen CE, Williams CKI. Gaussian Processes for Machine Learning. The MIT Press; 2005.

14. Marquand AF, Rezek I, Buitelaar J, Beckmann CF. Understanding Heterogeneity in Clinical Cohorts Using Normative Models: Beyond Case-Control Studies. Biol Psychiatry. 2016;80:552–561.

15. Rousseeuw PJ. Silhouettes: A graphical aid to the interpretation and validation of cluster analysis. J Comput Appl Math. 1987;20:53–65.

16. Wolfers T, Doan NT, Kaufmann T, Alnæs D, Moberget T, Agartz I, et al. Mapping the Heterogeneous Phenotype of Schizophrenia and Bipolar Disorder Using Normative Models. JAMA Psychiatry. 2018;75:1146–1155.

17. MacQueen J. Some methods for classification and analysis of multivariate observations1967.

18. Hartigan JA, Wong MA. A K-Means Clustering Algorithm. J R Stat Soc Ser C Appl Stat. 1979;28:100–108.

19. Selim SZ, Ismail MA. K-Means-Type Algorithms: A Generalized Convergence Theorem and Characterization of Local Optimality. IEEE Trans Pattern Anal Mach Intell. 1984;PAMI-6:81–87.

20. Charrad M, Ghazzali N, Boiteau V, Niknafs A. NbClust: An R Package for Determining the Relevant Number of Clusters in a Data Set. J Stat Softw. 2014;61:1–36.

21. Yeo BTT, Krienen FM, Sepulcre J, Sabuncu MR, Lashkari D, Hollinshead M, et al. The organization of the human cerebral cortex estimated by intrinsic functional connectivity. J Neurophysiol. 2011. 1 September 2011. https://doi.org/10.1152/jn.00338.2011.

22. Luo Z, Li W, Zhang F, Hu Z, You Z, Wang C, et al. Altered regional brain activity moderating the relationship between childhood trauma and depression severity. J Affect Disord. 2024;351:211–219.

23. Statistical power for cluster analysis | BMC Bioinformatics | Full Text. https://bmcbioinformatics.biomedcentral.com/articles/10.1186/s12859-022-04675-1. Accessed 21 October 2024.

24. Zhang Y, Huang C-C, Zhao J, Liu Y, Xia M, Wang X, et al. Dysfunction in sensorimotor and default mode networks in major depressive disorder with insights from global brain connectivity. Nat Ment Health. 2024;2:1371–1381.

25. Schmaal L, Hibar DP, Sämann PG, Hall GB, Baune BT, Jahanshad N, et al. Cortical abnormalities in adults and adolescents with major depression based on brain scans from 20 cohorts worldwide in the ENIGMA Major Depressive Disorder Working Group. Mol Psychiatry. 2017;22:900–909.

26. Dosenbach NUF, Nardos B, Cohen AL, Fair DA, Power JD, Church JA, et al. Prediction of Individual Brain Maturity Using fMRI. Science. 2010;329:1358–1361.

27. Kaiser RH, Andrews-Hanna JR, Wager TD, Pizzagalli DA. Large-Scale Network Dysfunction in Major Depressive Disorder: A Meta-analysis of Resting-State Functional Connectivity. JAMA Psychiatry. 2015;72:603–611.

28. Price RB, Duman R. Neuroplasticity in cognitive and psychological mechanisms of depression: an integrative model. Mol Psychiatry. 2020;25:530–543.

29. Martino M, Magioncalda P. A three-dimensional model of neural activity and phenomenal-behavioral patterns. Mol Psychiatry. 2024;29:639–652.

| **Supplementary Tables**  **Table S1.** Samples of the REST-meta-MDD project, consortium sites, sample size and data acquisition parameters on the cohorts | | | | | | | | | | | | | |
| --- | --- | --- | --- | --- | --- | --- | --- | --- | --- | --- | --- | --- | --- |
| Serial number | Sites (cohorts) | N | | Scanner | Receive (coil) | TR (ms) | TE (ms) | Flip angle (°) | Thickness/gap | Slice number | Time points | Voxel size | FOV |
|  |  | MDD | HCs |  |  |  |  |  |  |  |  |  |  |
| 1 | National Clinical Research Center for Mental Disorders (Peking University Sixth Hospital) & Key Laboratory of Mental Health, Ministry of Health (Peking University) | 74 | 74 | Siemens Tim Trio 3T | 32 channel | 2000 | 30 | 90 | 4.0mm/0.8mm | 30 | 210 | 3.28 × 3.28 × 4.80 | 210 × 210 |
| 2 | Department of Clinical Psychology, Suzhou Suzhou Psychiatric Hospital, The Affiliated Guangji Hospital of Soochow University | 30 | 30 | Philips Achieva 3T | 8-channel | 2000 | 30 | 90 | 4.0mm/0 mm | 37 | 200 | 1.67 × 1.67 × 4.00 | 240 × 240 |
| 3 | The Second Xiangya Hospital of Central South University | 27 | 37 | Siemens Magnetom Symphony scanner 1.5 T | 16 channel | 2000 | 40 | 90 | 5.0mm/1.25mm | 26 | 150 | 3.75 × 3.75 × 6.25 | 240 × 240 |
| 4 | The Second Xiangya Hospital of Central South University | 24 | 24 | Siemens Skyra 3T | 32 channel | 2500 | 25 | 90 | 3.5mm/0mm | 39 | 200 | 3.75 × 3.75 × 3.50 | 240 × 240 |
| 5 | Department of Psychiatry, Shanghai Jiao Tong University School of Medicine | 13 | 11 | GE Signa 3T | 32 channel | 3000 | 30 | 90 | 5.0mm/0mm | 22 | 100 | 3.75 × 3.75 × 5.00 | 240 × 240 |
| 6 | Department of Psychiatry, Shanghai Jiao Tong University School of Medicine | 15 | 15 | Siemens Tim Trio 3T | 32 channel | 2000 | 30 | 70 | 4mm/0mm | 33 | 180 | 3.59 × 3.59 × 4.00 | 230 × 230 |
| 7 | Sir Run Run Shaw Hospital, Zhejiang University School of Medicine | 38 | 49 | GE discovery MR750 | 8 channel | 2000 | 30 | 90 | 3.2/0 | 37 | 184 | 2.29 × 2.29 × 3.20 | 220 × 220 |
| 8 | Department of Psychiatry, First Affiliated Hospital, China Medical University | 75 | 75 | GE Signa 3T | 8 channel | 2000 | 30 | 90 | 3.0mm/0mm | 35 | 200 | 3.75 × 3.75 × 3.00 | 240 × 240 |
| 9 | The First Affiliated Hospital of Jinan University | 50 | 50 | GE Discovery MR750 3.0T | 8-channel | 2000 | 25 | 90 | 3.0/1.0 mm | 35 | 200 | 3.75 × 3.75 × 4.00 | 240 × 240 |
| 10 | First Hospital of Shanxi Medical University | 50 | 33 | Siemens Tim Trio 3T | 32 channel | 2000 | 30 | 90 | 3.0mm/1.52mm | 32 | 212 | 3.75 × 3.75 × 4.52 | 240 × 240 |
| 11 | Department of Psychiatry, The First Affiliated Hospital of Chongqing Medical University | 32 | 29 | GE Signa 3T | 8 channel | 2000 | 30 | 90 | 5 mm | 33 | 200 | 3.75 × 3.75 × 5.00 | 240 × 240 |
| 12 | Department of Psychiatry, The First Affiliated Hospital of Chongqing Medical University | 32 | 6 | GE Signa 3T | 8 channel | 2000 | 30 | 90 | 5 mm | 33 | 240 | 3.75 × 3.75 × 4.00 | 240 × 240 |
| 13 | The First Affiliated Hospital of Xi’an Jiaotong University, Xi’an Central Hospital | 25 | 17 | GE Excite 1.5T | 16 channel | 2500 | 35 | 90 | 4mm/0 | 36 | 150 | 4.00 × 4.00 × 4.00 | 256 × 256 |
| 14 | The Second Xiangya Hospital of Central South University | 64 | 32 | Siemens Tim Trio 3T | 32 channel | 2500 | 25 | 90 | 3.5/0 | 39 | 200 | 3.75 × 3.75 × 3.50 | 240 × 240 |
| 15 | Department of Psychosomatics and Psychiatry, Zhongda Hospital, School of Medicine, Southeast University | 50 | 50 | Siemens Verio 3.0T MRI | 12 channel | 2000 | 25 | 90 | 4mm/0mm | 36 | 240 | 3.75 × 3.75 × 4.00 | 240 × 240 |
| 16 | Huaxi MR Research Center, West China Hospital of Sichuan University | 31 | 31 | GE Signa 3T | 8 channel | 2000 | 30 | 90 | 5mm/0mm | 30 | 200 | 3.75 × 3.75 × 5.00 | 240 × 240 |
| 17 | Department of Psychiatry, The First Affiliated Hospital of Chongqing Medical University | 47 | 44 | GE Signa 3T | 8 channel | 2000 | 40 | 90 | 4.0mm/0mm | 33 | 240 | 3.75 × 3.75 × 4.00 | 240 × 240 |
| 18 | Department of Radiology, The First Affiliated Hospital, College of Medicine, Zhejiang University | 21 | 20 | Philips Achieva 3.0 T scanner (Philips Healthcare, Netherlands) | 8-channel SENSE head coil | 2000 | 35 | 90 | 5.0/1.0 mm | 24 | 200 | 1.67 × 1.67 × 6.00 | 240 × 240 |
| 19 | Anhui Medical University | 51 | 36 | GE Signa 3T | 8 channel | 2000 | 22.5 | 30 | 4.0/0.6 mm | 33 | 240 | 3.44 × 3.44 × 4.60 | 220 × 220 |
| 20 | Faculty of Psychology, Southwest University | 282 | 251 | Siemens Tim Trio 3T | 12 channel | 2000 | 30 | 90 | 3.0mm/1.0mm | 32 | 242 | 3.44 × 3.44 × 4.00 | 220 × 220 |
| 21 | Beijing Anding Hospital, Capital Medical University | 86 | 70 | Siemens Tim Trio 3T | 32 channel | 2000 | 30ms | 90 | 3.5mm/0.7mm | 33 | 240 | 3.12 × 3.12 × 4.20 | 200 × 200 |
| 22 | The Institute of Mental Health, Second Xiangya Hospital of Central South University | 30 | 20 | Philips Gyroscan Achieva 3.0T | 32 channel | 2000 | 30 | 90 | 4.0mm/0mm | 36 | 250 | 1.67 × 1.67 × 4.00 | 240 × 240 |
| 23 | Mental Health Center, West China Hospital, Sichuan University | 32 | 30 | Philips Achieva 3.0T TX | 8 channal | 2000 | 30 | 90 | 4.0mm/0mm | 38 | 240 | 3.75 × 3.75 × 4.00 | 240 × 240 |
| 24 | First Affiliated Hospital of Kunming Medical University | 32 | 31 | GE Signa 1.5T | 8 channel | 2000 | 40 | 90 | 5/1mm | 24 | 160 | 3.75 × 3.75 × 6.00 | 240 × 240 |
| 25 | Department of Neurology, Affiliated ZhongDa Hospital of Southeast University | 89 | 63 | Siemens Verio 3T | 12 channel head coil | 2000 | 25 | 90 | 4.0mm/0mm | 36 | 240 | 3.75 × 3.75 × 4.00 | 240 × 240 |
| Total |  | 1300 | 1128 |  |  |  |  |  |  |  |  |  |  |
| Abbreviations: MDD, major depressive disorder; HCs, healthy controls; TR, repetition time; TE, echo time; FOV, field of view; GE, general electric. | | | | | | | | | | | | | |

| **Table S2.** Demographic feature and clinical data for participants included in the primary analysis | | | |
| --- | --- | --- | --- |
|  |  |  |  |
|  | HCs (N=1011) | MDD (N=1101) | *t* or χ2/p/Cohen’s d or Cramer’s V |
| Age | 36.29 (15.78) | 36.48 (14.92) | -0.29/0.771/-0.01 |
| Sex: |  |  | **7.61/0.006/0.06** |
| Male | 420 (41.54%) | 392 (35.60%) |  |
| Female | 591 (58.46%) | 709 (64.40%) |  |
| Education years | 12.28 (4.99) | 11.22 (4.16) | **5.29/<0.001/0.23** |
| Episode: |  |  |  |
| First |  | 488 (67.68%) |  |
| Recurrent |  | 233 (32.32%) |  |
| Medication: |  |  |  |
| Yes |  | 341 (46.14%) |  |
| No |  | 398 (53.86%) |  |
| Illness duration (years) |  | 3.09 (5.07) |  |
| HAMD-17 total score |  | 20.14 (7.74) |  |
| HAMA-14 total score |  | 18.80 (9.04) |  |
| Mean FD (mm) | 0.07 (0.04) | 0.07 (0.04) | 0.79/0.429/0.03 |
| Data are presented as the mean (SD) or frequencies (proportion).  Abbreviations: HCs, healthy controls; MDD, major depressive disorder; HAMD-17, Hamilton Depression Rating Scale-17 item; HAMA-14, Hamilton Anxiety Rating Scale-14 item; FD, framewise displacement; mm, millimeter. | | | |
|  |  |  |  |
|  |  |  |  |
|  |  |  |  |

| **Table S3.** Clusters showing significant group differences in individual deviation maps between patients with MDD and HCs | | | | | | | |
| --- | --- | --- | --- | --- | --- | --- | --- |
| Clusters no. | Cluster sizes | Including regions | Network of peak | Peak T-value | Peak MNI coordinate (mm) | | |
|  |  |  |  |  | X | Y | Z |
| MDD > HCs | | | | | | | |
| 1 | 487 | Left inferior frontal gyrus, triangular part/middle frontal gyrus; BA 10/46 | FPCN | 3.27 | -39 | 45 | -3 |
|  |  |  |  |  |  |  |  |
| 2 | 374 | Right inferior temporal gyrus/middle temporal gyrus; BA 20/21 | LN | 3.10 | 57 | -18 | -36 |
| 3 | 40 | Right fusiform gyrus/parahippocampal gyrus; BA 36/37 | LN | 2.62 | 33 | -18 | -27 |
|  |  |  |  |  |  |  |  |
| MDD < HCs | | | | | | | |
| 1 | 4166 | Bilateral supplementary motor area/Bilateral paracentral lobule/Bilateral superior frontal gyrus/Bilateral median cingulate and paracingulate gyrus/Right middle frontal gyrus/Right inferior frontal gyrus, triangular part/Left postcentral gyrus/Left precentral gyrus; BA 3/4/6/8/9/46 | SMN | -5.47 | -60 | -9 | 15 |
|  |  |  |  |  |  |  |  |
| 2 | 1376 | Right postcentral gyrus/precentral gyrus/supramarginal gyrus/inferior parietal, but supramarginal and angular gyri/Rolandic operculum; BA 3/4/6/40 | SMN | -4.39 | 63 | 0 | 15 |
|  |  |  |  |  |  |  |  |
| 3 | 224 | Left middle occipital gyrus/inferior occipital gyrus; BA 19 | VN | -3.96 | -51 | -72 | -15 |
| 4 | 172 | Left thalamus | SUB | -3.25 | -6 | -21 | -6 |
| 5 | 700 | Right middle occipital gyrus/superior occipital gyrus; BA 19 | VN | -3.21 | 51 | -72 | -15 |
| 6 | 150 | Left posterior cingulate gyrus; BA31 | DMN | -3.00 | 0 | -51 | 21 |
| 7 | 122 | Left superior parietal gyrus/inferior parietal gyrus; BA7 | FPCN | -2.74 | -30 | -60 | 39 |
| Abbreviations: HCs, healthy controls; MDD, major depressive disorders; MNI, Montreal Neurological Institute; mm, millimeter; BA, Brodmann areas; FPCN, frontoparietal control network; LN, limbic network; SMN, sensorimotor network; VN, visual network; SUB, subcortical regions; DMN, default mode network. | | | | | | | |
|  |  |  |  |  |  |  |  |

| **Table S4.** Number of patients per subtype at each site | | |
| --- | --- | --- |
| Site | Subtype 1 | Subtype 2 |
| PKU | 38 (51.4%) | 36 (48.6%) |
| SU | 9 (32.1%) | 19 (67.9%) |
| CSU1 | 14 (58.3%) | 10 (41.7%) |
| SJTU | 2 (18.2%) | 9 (81.8%) |
| ZJU1 | 13 (34.2%) | 25 (65.8%) |
| CMU | 27 (36.0%) | 48 (64.0%) |
| JNU | 18 (36.0%) | 32 (64.0%) |
| SXMU | 19 (38.8%) | 30 (61.2%) |
| CQMU1 | 7 (46.7%) | 8 (53.3%) |
| CQMU2 | 9 (39.1%) | 14 (60.9%) |
| XJTU | 9 (42.9%) | 12 (57.1%) |
| CSU2 | 20 (35.1%) | 37 (64.9%) |
| SEU1 | 13 (29.5%) | 31 (70.5%) |
| SCU1 | 8 (34.8%) | 15 (65.2%) |
| CQMU3 | 21 (45.7%) | 25 (54.3%) |
| ZJU2 | 6 (60.0%) | 4 (40.0%) |
| AHMU | 4 (19.0%) | 17 (81%) |
| SWU | 102 (37.6%) | 169 (62.4%) |
| CCMU | 23 (28.4%) | 58 (71.6%) |
| CSU3 | 7 (28.0%) | 18 (72.0%) |
| SCU2 | 12 (37.5%) | 20 (62.5%) |
| SEU2 | 16 (19.3%) | 67 (80.7%) |
| Overall samples | 397 (36.1%) | 704 (63.9%) |
| Data are presented as the numbers (percentages). Abbreviations: PKU, Peking University; SU, Soochow University; CSU, Central South University; SJTU, Shanghai Jiao Tong University; ZJU, Zhejiang University; CMU, China Medical University; JNU, Jinan University; SXMU, Shanxi Medical University; CQMU, Chongqing Medical University; XJTU, Xi’an Jiaotong University; SEU, Southeast University; SCU, Sichuan University; AHMU, Anhui Medical University; SWU, Southwest University; CCMU, Capital Medical University. | | |
|  |  |  |
|  |  |  |
|  |  |  |
|  |  |  |
|  |  |  |
|  |  |  |

| **Table S5.** Number of patients per subtype after using k-means clustering at each site | | |
| --- | --- | --- |
|  |  |  |
| Site | Subtype 1 | Subtype 2 |
| PKU | 30 (40.5%) | 44 (59.5%) |
| SU | 6 (21.4%) | 22 (78.6%) |
| CSU1 | 11 (45.8%) | 13 (54.2%) |
| SJTU | 4 (36.4%) | 7 (63.6%) |
| ZJU1 | 12 (31.6%) | 26 (68.4%) |
| CMU | 29 (38.7%) | 46 (61.3%) |
| JNU | 13 (26.0%) | 37 (74.0%) |
| SXMU | 14 (28.6%) | 35 (71.4%) |
| CQMU1 | 5 (33.3%) | 10 (66.7%) |
| CQMU2 | 2 (8.7%) | 21 (91.3%) |
| XJTU | 9 (42.9%) | 12 (57.1%) |
| CSU2 | 20 (35.1%) | 37 (64.9%) |
| SEU1 | 19 (43.2%) | 25 (56.8%) |
| SCU1 | 10 (43.5%) | 13 (56.5%) |
| CQMU3 | 19 (41.3%) | 27 (58.7%) |
| ZJU2 | 4 (40.0%) | 6 (60.0%) |
| AHMU | 6 (28.6%) | 15 (71.4%) |
| SWU | 108 (39.9%) | 163 (60.1%) |
| CCMU | 22 (27.2%) | 59 (72.8%) |
| CSU3 | 7 (28.0%) | 18 (72.0%) |
| SCU2 | 10 (31.2%) | 22 (68.8%) |
| SEU2 | 16 (19.3%) | 67 (80.7%) |
| Overall samples | 376 (34.2%) | 725 (65.8%) |
| Data are presented as the numbers (percentages). Abbreviations: PKU, Peking University; SU, Soochow University; CSU, Central South University; SJTU, Shanghai Jiao Tong University; ZJU, Zhejiang University; CMU, China Medical University; JNU, Jinan University; SXMU, Shanxi Medical University; CQMU, Chongqing Medical University; XJTU, Xi’an Jiaotong University; SEU, Southeast University; SCU, Sichuan University; AHMU, Anhui Medical University; SWU, Southwest University; CCMU, Capital Medical University. | | |
|  |  |  |
|  |  |  |
|  |  |  |
|  |  |  |
|  |  |  |
|  |  |  |

| **Table S6.** Number of patients per subtype at each site (n > 30) | | |
| --- | --- | --- |
| Site | Subtype 1 | Subtype 2 |
| PKU | 38 (51.4%) | 36 (48.6%) |
| ZJU1 | 13 (34.2%) | 25 (65.8%) |
| CMU | 27 (36.0%) | 48 (64.0%) |
| JNU | 18 (36.0%) | 32 (64.0%) |
| SXMU | 19 (38.8%) | 30 (61.2%) |
| CSU2 | 20 (35.1%) | 37 (64.9%) |
| SEU1 | 13 (29.5%) | 31 (70.5%) |
| CQMU3 | 21 (45.7%) | 25 (54.3%) |
| SWU | 102 (37.6%) | 169 (62.4%) |
| CCMU | 23 (28.4%) | 58 (71.6%) |
| SCU2 | 12 (37.5%) | 20 (62.5%) |
| SEU2 | 16 (19.3%) | 67 (80.7%) |
| Overall samples | 322 (35.8%) | 578 (64.2%) |
| Data are presented as the numbers (percentages). Abbreviations: PKU, Peking University; ZJU, Zhejiang University; CMU, China Medical University; JNU, Jinan University; SXMU, Shanxi Medical University; CSU, Central South University; SEU, Southeast University; CQMU, Chongqing Medical University; SWU, Southwest University; CCMU, Capital Medical University; SCU, Sichuan University. | | |
|  |  |  |
|  |  |  |
|  |  |  |
|  |  |  |
|  |  |  |
|  |  |  |

| **Table S7.** Number of patients per subtype after using k-means clustering at each site (n > 30) | | |
| --- | --- | --- |
|  |  |  |
| Site | Subtype 1 | Subtype 2 |
| PKU | 30 (40.5%) | 44 (59.5%) |
| ZJU1 | 12 (31.6%) | 26 (68.4%) |
| CMU | 29 (38.7%) | 46 (61.3%) |
| JNU | 13 (26.0%) | 37 (74.0%) |
| SXMU | 14 (28.6%) | 35 (71.4%) |
| CSU2 | 20 (35.1%) | 37 (64.9%) |
| SEU1 | 19 (43.2%) | 25 (56.8%) |
| CQMU3 | 19 (41.3%) | 27 (58.7%) |
| SWU | 108 (39.9%) | 163 (60.1%) |
| CCMU | 22 (27.2%) | 59 (72.8%) |
| SCU2 | 10 (31.2%) | 22 (68.8%) |
| SEU2 | 16 (19.3%) | 67 (80.7%) |
| Overall samples | 312 (34.7%) | 588 (65.3%) |
| Data are presented as the numbers (percentages). Abbreviations: PKU, Peking University; ZJU, Zhejiang University; CMU, China Medical University; JNU, Jinan University; SXMU, Shanxi Medical University; CSU, Central South University; SEU, Southeast University; CQMU, Chongqing Medical University; SWU, Southwest University; CCMU, Capital Medical University; SCU, Sichuan University. | | |
|  |  |  |
|  |  |  |
|  |  |  |
|  |  |  |
|  |  |  |
|  |  |  |

| **Table S8.** Differences in mean deviation values at the network level between MDD subtypes | | | | | | | | |
| --- | --- | --- | --- | --- | --- | --- | --- | --- |
|  |  |  |  |  |  |  |  |  |
| Statistics/Network | FPCN | VAN | DMN | DAN | SMN | LN | VN | SUB |
| Subtype 1 mean (SD) | -0.36 (0.39) | -0.14 (0.49) | -0.32 (0.31) | 0.09 (0.37) | 0.25 (0.52) | -0.21 (0.51) | 0.43 (0.43) | -0.06 (0.59) |
| Subtype 2 mean (SD) | 0.32 (0.39) | 0.05 (0.44) | 0.22 (0.33) | -0.10 (0.35) | -0.28 (0.39) | 0.18 (0.51) | -0.32 (0.41) | -0.01 (0.54) |
| Subtype differences (*t*/*p*/Cohen's *d*) | **-27.86/< 0.001/-1.75** | **-6.20/< 0.001/-0.40** | **-27.55/< 0.001/-1.70** | **8.25/< 0.001/0.52** | **17.55/< 0.001/1.19** | **-12.27/< 0.001/-0.77** | **28.32/< 0.001/1.81** | -1.49/0.136/-0.10 |
| Abbreviations: FPCN, frontoparietal control network; VAN, ventral attention network; DMN, default mode network; DAN, dorsal attention network; SMN, sensorimotor network; LN, limbic network;  VN, visual network; SUB, subcortical regions. | | | | | | | | |
|  |  |  |  |  |  |  |  |  |
|  |  |  |  |  |  |  |  |  |

| **Table S9.** Group differences in deviation indices among MDD subtypes and HCs | | | |
| --- | --- | --- | --- |
|  |  |  |  |
| Comparison groups / Deviation indices | Overall deviation indices | Positive deviation indices | Negative deviation indices |
| Three Group  (*F*/*p*/Partial *η^2^*) | **4.68/0.009/0.00** | **5.75/0.003/0.01** | **20.87/< 0.001/0.02** |
| Subtype 1 vs. HCs (*t*/*p*/Cohen's *d*) | **-2.93/0.007/-0.18** | **2.90/0.004/0.18** | **-5.37/< 0.001/-0.33** |
| Subtype 2 vs. HCs (*t*/*p*/Cohen's *d*) | -0.86/0.394/-0.04 | 0.07/0.944/0.00 | 0.85/0.370/0.05 |
| Subtype 1 vs. Subtype 2 (*t*/*p*/Cohen's *d*) | **-2.11/0.041/-0.14** | **2.79/0.004/0.19** | **-5.77/< 0.001/-0.37** |

| **Table S10.** Group differences in the extreme deviation counts among MDD subtypes and HCs | | | |
| --- | --- | --- | --- |
|  |  |  |  |
| Comparison groups / Extreme deviation counts | Overall extreme deviation counts | Positive extreme deviation counts | Negative extreme deviation counts |
| Three Group  (*F*/*p*/Partial *η^2^*) | **4.71/0.009/0.00** | 2.43/0.089/- | **5.15/0.006/0.01** |
| Subtype 1 vs. HCs (*t*/*p*/Cohen's *d*) | **2.29/0.009/0.16** | - | **-2.02/0.006/-0.13** |
| Subtype 2 vs. HCs (*t*/*p*/Cohen's *d*) | -0.40/0.715/-0.02 | - | 1.83/0.775/0.09 |
| Subtype 1 vs. Subtype 2 (*t*/*p*/Cohen's *d*) | **2.46/0.009/0.17** | - | **-3.15/0.006/-0.23** |

| **Table S11.** Group differences in demographic feature and clinical data between MDD subtypes | | | |
| --- | --- | --- | --- |
|  |  |  |  |
|  | Subtype 1 (n = 397) | Subtype 2 (n = 704) | *t* or *χ2*/*p*/Cohen’s *d* or Cramer’s *V* |
| Age | 34.71 (14.44) | 37.48 (15.10) | **-3.01/0.003/-0.19** |
| Sex: |  |  | 0.05/0.829/0.01 |
| Male | 143 (36.02%) | 249 (35.37%) |  |
| Female | 254 (63.98%) | 455 (64.63%) |  |
| Education years | 11.11 (4.23) | 11.27 (4.12) | -0.62/0.536/-0.04 |
| Mean FD (mm) | 0.08 (0.04) | 0.07 (0.04) | **4.49/< 0.001/0.28** |
| Episode: |  |  | 0.02/0.899/0.01 |
| First | 172 (67.98%) | 316 (67.52%) |  |
| Recurrent | 81 (32.02%) | 152 (32.48%) |  |
| Medication: |  |  | **5.74/0.017/0.09** |
| Yes | 100 (40.00%) | 241 (49.28%) |  |
| No | 150 (60.00%) | 248 (50.72%) |  |
| Illness duration (years) | 2.99 (5.31) | 3.15 (4.94) | -0.43/0.669/-0.03 |
| HAMD-17 total score | 21.96 (6.98) | 19.69 (8.10) | **2.58/0.010/0.17** |
| HAMA-14 total score | 18.72 (9.19) | 18.85 (8.97) | -0.17/0.865/-0.01 |
| Items in HAMD-17 |  |  |  |
| Depressed Mood | 2.66 (0.96) | 2.59 (0.97) | 0.86/0.391/0.08 |
| Guilt | 1.21 (0.91) | 1.07 (0.89) | 1.51/0.132/0.15 |
| Suicide | 1.45 (1.17) | 1.45 (1.17) | -0.05/0.962/0.00 |
| Insomnia, intitial | 1.27 (0.78) | 1.33 (0.80) | -0.78/0.435/-0.07 |
| Insomnia, middle | 1.25 (0.71) | 1.23 (0.75) | 0.36/0.719/0.03 |
| Insomnia, delayed | 1.15 (0.83) | 1.13 (0.85) | 0.18/0.859/0.02 |
| Work and interests | 2.32 (1.05) | 2.31 (1.04) | 0.10/0.917/0.01 |
| Retardation | 1.29 (0.96) | 1.17 (0.94) | 1.25/0.212/0.12 |
| Agitation | 0.96 (0.91) | 0.94 (0.91) | 0.23/0.820/0.02 |
| Anxiety, psychic | 1.69 (0.98) | 1.81 (0.95) | -1.27/0.204/-0.12 |
| Anxiety, somatic | 1.45 (0.92) | 1.52 (0.99) | -0.81/0.421/-0.08 |
| Somatic, gastrointestinal | 0.81 (0.67) | 0.78 (0.64) | 0.46/0.643/0.05 |
| Somatic, general | 1.17 (0.75) | 1.16 (0.74) | 0.15/0.881/0.01 |
| Genital | 0.73 (0.74) | 0.79 (0.77) | -0.87/0.387/-0.08 |
| Hypochondriasis | 0.96 (0.96) | 0.99 (0.93) | -0.38/0.708/-0.04 |
| Loss of weight | 0.62 (0.79) | 0.57 (0.76) | 0.59/0.559/0.06 |
| Insight | 0.39 (0.58) | 0.54 (0.65) | **-2.62/0.009/-0.25** |
| anxiety/somatization | 6.50 (2.73) | 6.81 (2.90) | **-2.17/0.031/-0.12** |
| cognitive impairment | 3.61 (2.08) | 3.47 (2.05) | 0.73/0.465/0.07 |
| retardation | 6.98 (2.60) | 6.96 (2.49) | 0.49/0.624/0.05 |
| sleep disturbance | 3.67 (1.64) | 3.69 (1.87) | -0.12/0.905/-0.01 |
| Data are presented as the mean (SD) or frequencies (proportion).  Abbreviations: HAMD-17, Hamilton Depression Rating Scale-17 item; HAMA-14, Hamilton Anxiety Rating Scale-14 item. FD, framewise displacement; mm, millimeter. | | | |
|  |  |  |  |
|  |  |  |  |

| **Table 12.** Correlation between HAMD-17 total score and illness duration (years) by subtype | | | | | | |
| --- | --- | --- | --- | --- | --- | --- |
|  |  |  |  |  |  |  |
| Term | df | Sum square | Mean square | F-value | P-value | Partial *η^2^* |
| Illness duration (years) | 1 | 528.65 | 528.65 | 12.43 | **< 0.001** | 0.02 |
| Subtype | 1 | 7.14 | 7.14 | 0.17 | 0.682 | 0.00 |
| Illness duration (years) * Subtype | 1 | 96.97 | 96.97 | 2.28 | 0.132 | 0.00 |
| Residual | 771 | 3.28E+04 | 42.54 | - | - | - |
| Abbreviations: HAMD-17, Hamilton Depression Rating Scale-17 item; MDD, major depressive disorders. | | | | | | |

| **Table S13.** Group differences in demographic feature and clinical data between MDD subtypes in leave-one-site-out validation | | | | | | | | | | | |
| --- | --- | --- | --- | --- | --- | --- | --- | --- | --- | --- | --- |
|  |  |  |  |  |  |  |  |  |  |  |  |
| Leave-out Site | PKU | SU | CSU1 | SJTU | ZJU1 | CMU | JNU | SXMU | CQMU1 | CQMU2 | XJTU |
| Age | **-2.75/0.006/-0.17** | **-2.79/0.005/-0.18** | **-2.60/0.009/-0.17** | **-3.09/0.002/-0.20** | **-3.00/0.003/-0.19** | **-2.91/0.004/-0.19** | **-2.71/0.007/-0.17** | **-2.92/0.004/-0.19** | **-2.82/0.005/-0.18** | **-2.85/0.004/-0.18** | **-2.85/0.004/-0.18** |
| Sex (Male/Female) | 0.22/0.642/0.02 | 0.16/0.686/0.01 | 0.02/0.892/0.00 | 0.17/0.680/0.01 | 0.01/0.942/0.00 | 0.00/0.960/0.00 | 0.02/0.892/0.00 | 0.08/0.782/0.009 | 0.08/0.782/0.008 | 0.13/0.720/0.01 | 0.08/0.779/0.01 |
| Education years | -0.87/0.385/-0.06 | -0.72/0.475/-0.05 | 0.42/0.673/0.03 | -0.94/0.348/-0.06 | -0.38/0.706/-0.02 | -0.22/0.827/-0.01 | -0.52/0.602/-0.03 | -0.58/0.565/-0.04 | -0.50/0.616/-0.03 | -0.34/0.732/-0.02 | -0.71/0.480/-0.05 |
| Episode (First/Recurrent) | 0.00/0.989/-0.00 | 0.02/0.897/0.00 | 0.02/0.903/0.00 | 0.05/0.819/0.009 | 0.00/0.994/0.00 | 0.02/0.877/0.01 | 0.02/0.883/0.00 | 0.00/0.955/0.00 | 0.12/0.728/0.013 | 0.03/0.869/0.01 | 0.06/0.810/0.01 |
| Medication (Yes/No) | **5.26/0.022/0.08** | **6.21/0.013/0.09** | **5.26/0.022/0.08** | **6.78/0.009/0.10** | **4.60/0.032/0.08** | **6.77/0.090/0.10** | **4.76/0.029/0.08** | **4.64/0.031/0.08** | **6.12/0.013/0.092** | **5.14/0.023/0.09** | **4.82/0.028/0.08** |
| Illness duration (years) | 0.01/0.793/0.00 | -0.79/0.428/-0.06 | -0.63/0.530/-0.05 | -0.52/0.604/-0.04 | -0.54/-0.203/-0.04 | -0.85/0.397/-0.06 | -0.56/0.573/-0.04 | -0.72/0.469/-0.05 | -0.65/0.515/-0.05 | -0.33/0.742/-0.02 | -0.41/0.680/-0.03 |
| HAMD-17 total score | 1.93/0.054/0.13 | **2.56/0.011/0.17** | **2.51/0.012/0.17** | **2.49/0.013/0.17** | **2.21/0.027/0.15** | **2.54/0.011/0.18** | **2.38/0.018/0.16** | **1.98/0.048/0.14** | **2.15/0.032/0.14** | **2.28/0.023/0.16** | **2.34/0.020/0.16** |
| HAMA-14 total score | -0.28/0.783/-0.02 | -0.17/0.865/-0.01 | 0.00/1.000/0.00 | -0.42/0.676/-0.04 | -0.26/0.792/-0.02 | 0.30/0.767/0.03 | -0.41/0.681/-0.04 | -0.41/0.685/-0.04 | -0.40/0.693/-0.03 | -0.40/0.693/-0.03 | -0.17/0.865/-0.01 |
| Items in HAMD-17 |  |  |  |  |  |  |  |  |  |  |  |
| Depressed Mood | 0.31/0.755/0.03 | 0.79/0.429/0.08 | 0.80/0.427/0.08 | 0.86/0.391/0.08 | 0.51/0.614/0.05 | 0.75/0.455/0.08 | 0.68/0.495/0.07 | 0.68/0.495/0.07 | 0.74/0.460/0.07 | 0.70/0.487/0.07 | 0.68/0.495/0.07 |
| Guilt | 0.84/0.400/0.09 | 1.46/0.145/0.15 | 1.61/0.108/0.16 | 1.52/0.129/0.15 | 1.47/0.143/0.15 | 1.41/0.159/0.15 | 1.44/0.150/0.14 | 1.44/0.150/0.14 | 1.42/0.157/0.14 | 1.32/0.187/0.13 | 1.44/0.150/0.14 |
| Suicide | -0.25/0.804/-0.03 | -0.08/0.937/-0.01 | -0.08/0.933/-0.01 | -0.05/0.962/-0.00 | 0.07/0.947/0.00 | 0.62/0.538/0.07 | -0.14/0.890/-0.01 | -0.14/0.890/-0.01 | -0.25/0.800/-0.03 | 0.10/0.923/0.01 | -0.14/0.890/-0.01 |
| Insomnia, intitial | 0.22/0.823/0.02 | -0.54/0.591/-0.05 | -0.81/0.417/-0.08 | -0.78/0.438/-0.08 | -0.37/0.714/-0.04 | -0.54/0.591/-0.06 | 0.58/0.561/-0.06 | -0.58/0.561/-0.06 | 0.50/0.617/-0.05 | -0.81/0.418/-0.08 | -0.58/0.561/-0.06 |
| Insomnia, middle | -0.03/0.974/-0.00 | 0.48/0.635/0.05 | 0.32/0.747/0.03 | 0.36/0.723/0.03 | 0.33/0.739/0.03 | 0.56/0.574/0.06 | 0.29/0.775/0.03 | 0.29/0.775/0.03 | 0.12/0.901/0.01 | 0.31/0.756/0.03 | 0.29/0.775/0.03 |
| Insomnia, delayed | 0.58/0.566/0.06 | 0.61/0.540/0.06 | 0.28/0.783/0.03 | 0.18/0.859/0.02 | 0.48/0.633/0.05 | -0.03/0.977/-0.01 | 0.21/0.834/0.02 | 0.21/0.834/0.02 | 0.18/0.856/0.18 | 0.31/0.761/0.03 | 0.21/0.834/0.02 |
| Work and interests | 0.04/0.965/0.00 | 0.09/0.931/0.01 | 0.08/0.940/0.00 | 0.11/0.916/0.01 | -0.02/0.984/-0.00 | -0.25/0.802/-0.03 | 0.07/0.943/0.01 | 0.07/0.943/0.01 | -0.04/0.968/-0.00 | 0.21/0.832/0.02 | 0.07/0.943/0.01 |
| Retardation | 0.46/0.644/0.05 | 1.22/0.224/0.12 | 1.23/0.22/0.12 | 1.26/0.209/0.12 | 1.24/0.215/0.13 | 1.77/0.077/0.19 | 1.20/0.230/0.12 | 1.20/0.230/0.12 | 1.24/0.214/0.12 | 1.30/0.195/0.13 | 1.20/0.230/0.12 |
| Agitation | 0.01/0.990/0.00 | 0.34/0.734/0.03 | 0.34/0.734/0.03 | 0.23/0.819/0.02 | 0.33/0.739/0.03 | 1.53/0.128/0.16 | 0.32/0.746/0.03 | 0.32/0.746/0.03 | 0.08/0.935/0.01 | 0.13/0.900/-0.11 | 0.32/0.746/0.03 |
| Anxiety, psychic | -1.96/0.051/-0.21 | -1.21/0.228/-0.12 | -1.26/0.209/-0.12 | -1.28/0.200/-0.12 | -0.9/0.368/-0.09 | 0.15/0.885/0.02 | -1.23/0.219/-0.12 | -1.23/0.219/-0.12 | -1.28/0.202/-0.12 | -.15/0.252/-0.05 | -1.23/0.219/-0.12 |
| Anxiety, somatic | -1.23/0.217/-0.13 | -0.80/0.228/-0.08 | -0.74/0.461/-0.07 | -0.79/0.430/-0.08 | -0.62/0.533/-0.06 | 0.24/0.809/0.03 | 0.69/0.489/-0.07 | -0.69/0.489/-0.07 | -0.65/0.517/-0.06 | -0.49/0.626/0.07 | -0.69/0.489/-0.07 |
| Somatic, gastrointestinal | 0.36/0.717/0.04 | 0.47/0.639/0.05 | 0.50/0.618/0.05 | 0.47/0.639/0.05 | 0.69/0.492/0.07 | 0.23/0.820/0.02 | 0.56/0.578/0.05 | 0.56/0.578/0.05 | 0.54/0.588/0.05 | 0.66/0.509/0.03 | 0.56/0.578/0.05 |
| Somatic, general | 0.22/0.827/0.02 | 0.17/0.868/0.02 | 0.13/0.898/0.01 | 0.15/0.881/0.01 | 0.11/0.914/0.01 | 0.45/0.656/0.05 | 0.19/0.847/0.02 | 0.19/0.847/0.02 | 0.07/0.946/0.01 | 0.29/0.778/-0.08 | 0.19/0.847/0.02 |
| Genital | 0.78/0.437/-0.08 | -0.61/0.539/-0.06 | -0.95/0.342/-0.09 | -0.86/0.393/-0.08 | -1.27/0.203/-0.13 | -1.56/0.119/-0.17 | -0.92/0.360/-0.09 | -0.92/0.360/-0.09 | -0.89/0.374/0.09 | -0.84/0.403/-0.08 | -0.92/0.360/-0.09 |
| Hypochondriasis | -0.79/0.432/-0.08 | -0.49/0.624/-0.05 | -0.38/0.706/-0.04 | -0.38/0.7015/-0.04 | -0.16/0.871/-0.02 | 0.76/0.448/0.08 | -0.28/0.778/-0.03 | -0.28/0.778/-0.03 | -0.42/0.678/-0.04 | -0.48/0.630/-0.05 | -0.28/0.778/-0.03 |
| Loss of weight | 0.30/0.762/0.03 | 0.66/0.512/0.07 | 0.52/0.606/0.05 | 0.59/0.555/0.06 | 0.68/0.497/0.07 | 0.41/0.683/0.04 | 0.49/0.625/0.05 | 0.49/0.625/0.05 | 0.43/0.665/-0.04 | 0.93/0.354/0.09 | 0.49/0.625/0.05 |
| Insight | -1.95/0.052/-0.21 | **-2.58/0.010/-0.26** | **-2.61/0.009/-0.25** | **-0.51/0.011/-0.25** | **-2.65/0.008/-0.27** | **-2.52/0.012/-0.27** | **-2.39/0.017/-0.23** | **-2.39/0.017/-0.23** | **-2.38/0.018/-0.23** | **-2.45/0.015/-0.24** | **-2.39/0.017/-0.23** |
| Correlation between HAMD-17 total score and illness duration (years) |  |  |  |  |  |  |  |  |  |  |  |
|  |  |  |  |  |  |  |  |  |  |  |  |
|  |  |  |  |  |  |  |  |  |  |  |  |
|  |  |  |  |  |  |  |  |  |  |  |  |
| Illness_duration (years) | **7.26/0.007/0.00** | **11.26/< 0.001/0.00** | **12.43/< 0.001/0.00** | **12.43/< 0.001/0.00** | **12.40/< 0.001/0.00** | **13.64/< 0.001/0.00** | **12.42/< 0.001/0.00** | **13.04/<0.001/0.00** | **12.35/< 0.001/0.00** | **12.37/< 0.001/0.00** | **12.42/< 0.001/0.00** |
|  |  |  |  |  |  |  |  |  |  |  |  |
|  |  |  |  |  |  |  |  |  |  |  |  |
| Post-hoc (Subtype 1) | **-0.18/0.005** | **-0.21/< 0.001** | **-0.21/< 0.001** | **-0.21/< 0.001** | **-0.20/0.001** | **0.24/< 0.001** | **-0.20/< 0.001** | **-0.21/ < 0.001** | **-0.20/< 0.001** | **-0.20/0.001** | **-0.20/< 0.001** |
| Post-hoc (Subtype 2) | -0.06/0.212 | -0.08/0.094 | -0.08/0.080 | -0.08/0.080 | -0.09/0.056 | -0.08/0.085 | -0.08/0.068 | -0.09/0.057 | -0.08/0.066 | -0.08/0.061 | -0.08/0.068 |
| Note: Missing HAMD-17 item scores at some sites led to the use of leave-one-site-out validation, resulting in the same sample across analyses and potentially similar statistical outcomes. Data are presented as the t, χ2, F, or r/p/Cohen’s d, Cramer's V or Partial η2. Abbreviations: PKU, Peking University; SU, Soochow University; CSU, Central South University; SJTU, Shanghai Jiao Tong University; ZJU, Zhejiang University; CMU, China Medical University; JNU, Jinan University; SXMU, Shanxi Medical University; CQMU, Chongqing Medical University; XJTU, Xi’an Jiaotong University; SEU, Southeast University; SCU, Sichuan University; AHMU, Anhui Medical University; SWU, Southwest University; CCMU, Capital Medical University, HAMD-17, Hamilton Depression Rating Scale-17 item; HAMA-14, Hamilton Anxiety Rating Scale-14 item. | | | | | | | | | | |  |
|  |  |  |  |  |  |  |  |  |  |  |  |
|  |  |  |  |  |  |  |  |  |  |  |  |
|  |  |  |  |  |  |  |  |  |  |  |  |
|  |  |  |  |  |  |  |  |  |  |  |  |
|  |  |  |  |  |  |  |  |  |  |  |  |
| Leave-out Site | CSU2 | SEU1 | SCU1 | CQMU3 | ZJU2 | AHMU | SWU | CCMU | CSU3 | SCU2 | SEU2 |
| Age | **-3.07/0.002/-0.20** | **-2.82/0.005/-0.18** | **-3.26/0.001/-0.21** | **-2.52/0.012/-0.16** | **-3.01/0.003/-0.19** | **-2.91/0.004/-0.18** | **-3.49/0.001/-0.25** | **-3.41/0.001/-0.22** | **-3.23/0.001/-0.21** | **-2.93/0.003/-0.19** | -1.39/0.166/-0.09 |
| Sex (Male/Female) | 0.08/0.782/0.01 | 0.05/0.823/0.01 | 0.03/0.875/0.01 | 0.05/0.818/0.01 | 0.14/0.705/0.01 | 0.06/0.811/0.01 | 0.47/0.492/0.02 | 0.42/0.516/0.02 | 0.03/0.874/0.01 | 0.00/0.992/0.00 | 0.08/0.780/0.01 |
| Education years | -0.25/0.800/-0.02 | -0.84/0.404/-0.05 | -0.46/0.647/-0.03 | -0.87/0.386/-0.06 | -0.75/0.453/-0.05 | -0.71/0.476/-0.05 | -0.45/0.657/-0.03 | -0.14/0.889/-0.01 | -0.59/0.557/-0.04 | -0.63/0.530/-0.04 | -1.04/0.300/-0.07 |
| Episode (First/Recurrent) | 0.03/0.857/0.01 | 0.03/0.857/0.01 | 0.01/0.942/0.00 | 0.04/0.852/0.01 | 0.02/0.899/0.01 | 0.04/0.852/0.01 | 0.05/0.823/0.01 | 0.40/0.526/0.03 | 0.02/0.899/0.01 | 0.26/0.613/0.02 | 0.10/0.755/0.01 |
| Medication (Yes/No) | **4.44/0.035/0.08** | **5.02/0.025/0.08** | **5.07/0.024/0.08** | **5.49/0.019/0.09** | **5.74/0.017/0.09** | **4.96/0.026/0.08** | 1.70/0.192/0.06 | **5.66/0.017/0.09** | **5.76/0.016/0.09** | **5.45/0.020/0.09** | 3.54/0.060/0.07 |
| Illness duration (years) | -0.57/0.566/-0.04 | -0.49/0.625/-0.04 | -0.67/0.505/-0.05 | -0.38/0.701/-0.03 | -0.33/0.742/-0.02 | -0.16/0.876/-0.01 | -0.58/0.563/-0.05 | -0.45/0.652/-0.03 | -0.44/0.660/-0.03 | -0.44/0.661/-0.03 | -0.39/0.697/-0.03 |
| HAMD-17 total score | **2.21/0.027/0.15** | **2.61/0.009/0.18** | **2.21/0.027/0.15** | **2.29/0.022/0.16** | **2.41/0.016/0.16** | **2.47/0.014/0.17** | **2.29/0.022/0.18** | **2.61/0.009/0.18** | **2.41/0.016/0.16** | **2.24/0.026/0.15** | 0.69/0.492/0.05 |
| HAMA-14 total score | -0.30/0.764/-0.03 | -0.06/0.954/-0.01 | -0.46/0.649/-0.04 | 0.13/0.898/0.01 | -0.35/0.726/-0.03 | -0.17/0.865/-0.01 | -0.91/0.366/-0.10 | -0.09/0.933/-0.01 | -0.35/0.728/-0.03 | -0.17/0.865/-0.01 | -0.47/0.637/-0.04 |
| Items in HAMD-17 |  |  |  |  |  |  |  |  |  |  |  |
| Depressed Mood | 0.95/0.344/0.10 | 0.86/0.391/0.08 | 0.75/0.457/0.07 | 0.72/0.472/0.07 | 0.64/0.520/0.06 | 0.89/0.376/0.09 | 0.78/0.434/0.08 | 0.83/0.407/0.09 | 0.99/0.323/0.10 | 0.56/0.576/0.06 | 0.75/0.457/0.07 |
| Guilt | 1.22/0.223/0.13 | 1.52/0.129/0.15 | 1.46/0.146/0.14 | 1.54/0.126/0.15 | 1.77/0.077/0.17 | 1.46/0.144/0.14 | 1.66/0.098/0.16 | 1.70/0.091/0.18 | 1.46/0.145/0.14 | 1.35/0.178/0.14 | 1.46/0.146/0.14 |
| Suicide | -0.05/0.961/-0.01 | -0.05/0.962/-0.01 | -0.02/0.985/-0.00 | -0.18/0.861/-0.02 | 0.11/0.913/0.01 | -0.32/0.748/-0.03 | -0.30/0.762/-0.03 | -0.69/0.490/-0.07 | -0.23/0.819/-0.02 | -0.11/0.912/-0.01 | -0.43/0.666/-0.04 |
| Insomnia, intitial | -0.99/0.323/-0.10 | -0.78/0.438/-0.08 | -0.55/0.586/-0.05 | -0.74/0.46/-0.07 | -0.90/0.370/-0.09 | -0.74/0.461/-0.07 | -0.83/0.410/-0.08 | -0.95/0.344/-0.10 | -1.12/0.265/-0.11 | -0.41/0.682/-0.04 | -0.91/0.363/-0.09 |
| Insomnia, middle | 0.80/0.425/0.08 | 0.36/0.723/0.03 | 0.32/0.751/0.03 | 0.26/0.799/0.03 | 0.05/0.963/0.01 | 0.14/0.891/0.01 | 0.16/0.877/0.02 | 0.34/0.733/0.04 | 0.34/0.732/0.03 | 0.27/0.784/0.03 | 0.19/0.852/0.02 |
| Insomnia, delayed | 0.78/0.436/0.08 | 0.18/0.859/0.02 | 0.34/0.734/0.03 | 0.19/0.847/0.02 | 0.07/0.944/0.01 | 0.03/0.975/0.00 | 0.32/0.746/0.03 | -0.80/0.423/-0.08 | -0.08/0.939/-0.01 | 0.18/0.856/0.02 | 0.45/0.650/0.04 |
| Work and interests | 0.12/0.904/0.01 | 0.11/0.916/0.01 | 0.10/0.920/0.01 | 0.04/0.966/0.00 | 0.05/0.962/0.01 | 0.12/0.904/0.01 | 0.16/0.870/0.02 | 0.75/0.453/0.08 | -0.04/0.972/0.00 | -0.10/0.924/-0.01 | 0.10/0.920/0.01 |
| Retardation | 1.35/0.177/0.14 | 1.26/0.209/0.12 | 1.23/0.221/0.12 | 1.18/0.239/0.11 | 1.37/0.172/0.13 | 1.21/0.227/0.12 | 1.30/0.193/0.13 | 0.67/0.504/0.07 | 1.08/0.282/0.11 | 1.13/0.261/0.11 | 1.23/0.221/0.12 |
| Agitation | 0.32/0.748/0.03 | 0.23/0.819/0.02 | 0.32/0.750/0.03 | 0.22/0.824/0.02 | -0.04/0.969/0.00 | 0.05/0.957/0.01 | 0.43/0.668/0.04 | 0.26/0.796/0.03 | -0.07/0.947/-0.01 | 0.28/0.778/0.03 | 0.53/0.596/0.05 |
| Anxiety, psychic | -1.44/0.150/-0.15 | -1.28/0.200/-0.12 | -1.26/0.209/-0.12 | -1.21/0.228/-0.12 | -1.58/0.114/-0.16 | -1.46/0.145/-0.14 | -1.13/0.259/-0.11 | -1.12/0.262/-0.12 | -1.49/0.138/-0.15 | -1.08/0.280/-0.11 | -1.16/0.248/-0.11 |
| Anxiety, somatic | -0.47/0.636/-0.05 | -0.79/0.430/-0.08 | -0.74/0.457/-0.07 | -0.84/0.401/-0.08 | -0.93/0.351/-0.09 | -0.96/0.336/-0.10 | -0.69/0.489/-0.07 | -0.94/0.350/-0.10 | -1.06/0.288/-0.11 | -0.64/0.526/-0.06 | -0.85/0.399/-0.08 |
| Somatic, gastrointestinal | 0.49/0.623/0.05 | 0.47/0.639/0.05 | 0.53/0.598/0.05 | 0.44/0.661/0.04 | 0.53/0.597/0.05 | 0.47/0.636/0.05 | 0.56/0.578/0.05 | 0.21/0.833/0.02 | 0.75/0.453/0.07 | 0.38/0.703/0.04 | 0.38/0.705/0.04 |
| Somatic, general | 0.09/0.930/0.01 | 0.15/0.881/0.01 | 0.22/0.830/0.02 | 0.04/0.967/0.00 | 0.51/0.613/0.05 | 0.15/0.878/0.02 | 0.19/0.847/0.02 | -0.57/0.569/-0.06 | 0.10/0.918/0.01 | 0.24/0.812/0.02 | 0.08/0.933/0.01 |
| Genital | -0.08/0.935/-0.01 | -0.86/0.393/-0.08 | -0.95/0.344/-0.09 | -0.89/0.376/-0.09 | -0.82/0.412/-0.08 | -0.90/0.371/-0.09 | -0.92/0.360/-0.09 | -1.05/0.296/-0.11 | -0.78/0.438/-0.08 | -0.87/0.385/-0.09 | -1.08/0.282/-0.10 |
| Hypochondriasis | -0.33/0.742/-0.03 | -0.38/0.705/-0.04 | -0.39/0.699/-0.04 | -0.48/0.628/-0.05 | -0.39/0.696/-0.04 | -0.47/0.639/-0.05 | -0.38/0.701/-0.04 | -0.62/0.533/-0.07 | -0.40/0.689/-0.04 | -0.11/0.909/-0.01 | -0.28/0.777/-0.03 |
| Loss of weight | 0.05/0.960/0.01 | 0.59/0.555/0.06 | 0.56/0.574/0.06 | 0.54/0.590/0.05 | 0.29/0.772/0.03 | 0.74/0.462/0.07 | 0.61/0.539/0.06 | 0.11/0.916/0.01 | 0.93/0.355/0.09 | 0.59/0.555/0.06 | 0.69/0.491/0.07 |
| Insight | **-2.67/0.008/-0.28** | **-2.54/0.011/-0.25** | **-2.32/0.021/-0.23** | **-2.47/0.014/-0.24** | **-2.22/0.027/-0.22** | **-2.40/0.017/-0.24** | **-2.55/0.011/-0.25** | -1.77/0.077/-0.19 | **-2.28/0.023/-0.23** | **-2.32/0.021/-0.23** | **-2.48/0.014/-0.24** |
| Correlation between HAMD-17 total score and illness duration (years) |  |  |  |  |  |  |  |  |  |  |  |
|  |  |  |  |  |  |  |  |  |  |  |  |
|  |  |  |  |  |  |  |  |  |  |  |  |
|  |  |  |  |  |  |  |  |  |  |  |  |
| Illness_duration (years) | **11.83/< 0.001/0.00** | **11.39/< 0.001/0.00** | **11.96/< 0.001/0.00** | **12.42/< 0.001/0.00** | **11.97/< 0.001/0.00** | **16.00/< 0.001/0.00** | **15.57/< 0.001/0.00** | 1.53/0.217/0.00 | **12.04/< 0.001/0.00** | **14.13/< 0.001/0.00** | **12.42/< 0.001/0.00** |
|  |  |  |  |  |  |  |  |  |  |  |  |
|  |  |  |  |  |  |  |  |  |  |  |  |
| Post-hoc (Subtype 1) | **-0.21/< 0.001** | **-0.20/< 0.001** | **-0.20/< 0.001** | **-0.20/< 0.001** | **-0.21/< 0.001** | **-0.22/< 0.001** | **-0.26/< 0.001** | **-** | **-0.21/< 0.001** | **-0.21/< 0.001** | **-0.20/< 0.001** |
| Post-hoc (Subtype 2) | -0.08/0.077 | -0.07/0.106 | -0.08/0.073 | -0.08/0.068 | -0.08/0.085 | **-0.10/0.034** | **-0.11/0.038** | **-** | -0.08/0.087 | **-0.09/0.039** | -0.08/0.061 |
| Note: Missing HAMD-17 item scores at some sites led to the use of leave-one-site-out validation, resulting in the same sample across analyses and potentially similar statistical outcomes. Data are presented as the t, χ2, F, or r/p/Cohen’s d, Cramer's V or Partial η2. Abbreviations: PKU, Peking University; SU, Soochow University; CSU, Central South University; SJTU, Shanghai Jiao Tong University; ZJU, Zhejiang University; CMU, China Medical University; JNU, Jinan University; SXMU, Shanxi Medical University; CQMU, Chongqing Medical University; XJTU, Xi’an Jiaotong University; SEU, Southeast University; SCU, Sichuan University; AHMU, Anhui Medical University; SWU, Southwest University; CCMU, Capital Medical University, HAMD-17, Hamilton Depression Rating Scale-17 item; HAMA-14, Hamilton Anxiety Rating Scale-14 item. | | | | | | | | | | | |
|  |  |  |  |  |  |  |  |  |  |  |  |
|  |  |  |  |  |  |  |  |  |  |  |  |
|  |  |  |  |  |  |  |  |  |  |  |  |
|  |  |  |  |  |  |  |  |  |  |  |  |

| **Table S14.** Group differences in demographic feature and clinical data between MDD subtypes in leave-one-site-out validation (n > 30). | | | | | | | | | | | | |
| --- | --- | --- | --- | --- | --- | --- | --- | --- | --- | --- | --- | --- |
|  |  |  |  |  |  |  |  |  |  |  |  |  |
| Leave-out site | PKU | ZJU1 | CMU | JNU | SXMU | CSU2 | SEU1 | CQMU3 | SWU | CCMU | SCU2 | SEU2 |
| Age | **-2.04/0.042/-0.15** | **-2.48/0.013/-0.18** | **-2.57/0.010/-0.19** | **-2.23/0.026/-0.16** | **-2.59/0.010/-0.19** | **-2.48/0.013/-0.18** | **-2.28/0.023/-0.16** | **-2.14/0.032/-0.15** | **-3.44/0.001/-0.29** | **-2.90/0.004/-0.21** | **-2.60/0.010/-0.18** | -1.06/0.289/-0.08 |
| Sex(Male/Female) | 0.69/0.405/0.03 | 0.22/0.641/0.02 | 0.38/0.538/0.02 | 0.30/0.587/0.02 | 0.72/0.396/0.03 | 0.42/0.516/0.02 | 1.35/0.245/0.04 | 0.51/0.474/0.02 | 3.62/0.057/0.08 | 1.50/0.220/0.04 | 0.40/0.526/0.02 | 1.61/0.205/0.04 |
| Education_years | -0.77/0.441/-0.06 | -0.16/0.874/-0.01 | -0.04/0.967/-0.00 | -0.28/0.779/-0.02 | -0.50/0.616/-0.04 | -0.24/0.809/-0.02 | -0.71/0.480/-0.05 | -0.63/0.527/-0.05 | -0.53/0.597/-0.04 | -0.05/0.961/-0.00 | -0.25/0.804/-0.02 | -0.74/0.458/-0.05 |
| Episode(First/Recurrent) | 0.38/0.536/0.03 | 0.07/0.787/0.01 | 0.01/0.943/0.03 | 0.00/0.999/0.00 | 0.05/0.822/0.01 | 0.04/0.837/-0.01 | 0.01/0.912/0.00 | 0.01/0.934/0.00 | 0.57/0.451/0.04 | 1.28/0.257/0.05 | 0.10/0.757/0.01 | 0.03/0.853/-0.01 |
| Medication(Yes/No) | **3.98/0.046/0.08** | **4.19/0.041/0.09** | **9.43/0.002/0.14** | **5.65/0.017/0.10** | **5.34/0.021/0.10** | **5.59/0.018/0.10** | **4.83/0.028/0.09** | **5.21/0.022/0.10** | 2.37/0.123/0.08 | **6.19/0.013/0.10** | **5.60/0.018/0.10** | **3.05/0.081/0.08** |
| Illness duration (years) | 0.29/0.772/0.02 | -0.10/0.807/-0.02 | -0.38/0.706/-0.03 | -0.36/0.717/-0.03 | -0.44/0.662/-0.04 | -0.59/0.554/-0.05 | -0.70/0.484/-0.06 | -0.53/0.599/-0.04 | -0.27/0.785/-0.03 | -0.13/0.897/-0.01 | -0.23/0.820/-0.02 | -0.42/0.673/-0.03 |
| HAMD-17 total score | **2.12/0.034/0.16** | **2.15/0.039/0.15** | **2.97/0.003/0.22** | **2.51/0.012/0.18** | **2.30/0.022/0.17** | **2.28/0.023/0.16** | **2.48/0.014/0.18** | **2.37/0.018/0.17** | **2.69/0.007/0.22** | **2.70/0.007/0.20** | **2.23/0.026/0.16** | 0.32/0.746/0.03 |
| HAMA-14 total score | -0.57/0.570/-0.05 | -0.99/0.322/-0.09 | -0.11/0.909/-0.01 | -0.86/0.389/-0.08 | -0.68/0.494/-0.06 | -0.77/0.440/-0.07 | -0.71/0.479/-0.06 | -0.48/0.628/-0.04 | -1.94/0.054/-0.23 | -0.64/0.525/-0.06 | -0.85/0.394/-0.07 | -1.12/0.265/-0.10 |
| Items in HAMD-17 |  |  |  |  |  |  |  |  |  |  |  |  |
| Depressed Mood | 0.32/0.753/0.04 | 0.42/0.677/0.05 | 0.94/0.347/0.12 | 0.82/0.412/0.09 | 0.76/0.448/0.08 | 0.80/0.424/0.10 | 0.68/0.498/0.07 | 0.70/0.487/0.08 | 0.82/0.412/0.09 | 0.56/0.574/0.07 | 0.68/0.500/0.08 | 0.91/0.363/0.10 |
| Guilt | 0.72/0.471/0.09 | 1.39/0.166/0.16 | 1.46/0.145/0.18 | 1.46/0.146/0.16 | 1.56/0.120/0.17 | 0.97/0.335/0.12 | 1.39/0.165/0.15 | 1.42/0.155/0.16 | 1.34/0.181/0.15 | 1.66/0.099/0.20 | 1.26/0.209/0.15 | 1.19/0.235/0.13 |
| Suicide | -0.54/0.591/-0.07 | -0.14/0.888/-0.02 | 0.64/0.523/0.08 | -0.26/0.798/-0.03 | -0.29/0.770/-0.03 | -0.58/0.561/-0.07 | -0.40/0.686/-0.04 | -0.42/0.674/-0.05 | -0.44/0.661/-0.05 | -1.13/0.258/-0.14 | -0.19/0.853/-0.02 | -0.70/0.487/-0.08 |
| Insomnia, intitial | -0.00/0.999/0.00 | -0.66/0.509/-0.08 | -1.15/0.250/-0.14 | -1.13/0.260/-0.12 | -1.17/0.244/-0.13 | **-2.04/0.042/-0.24** | -1.42/0.157/-0.16 | -1.34/0.180/-0.15 | -1.40/0.162/-0.15 | -1.52/0.130/-0.19 | -0.91/0.363/-0.11 | -1.34/0.180/-0.15 |
| Insomnia, middle | -0.71/0.479/-0.09 | -0.28/0.780/-0.03 | 0.03/0.976/0.00 | -0.23/0.815/-0.03 | -0.26/0.792/-0.03 | -0.04/0.966/-0.01 | -0.50/0.616/-0.06 | -0.44/0.659/-0.05 | -0.68/0.499/-0.07 | -0.46/0.644/-0.06 | -0.24/0.807/-0.03 | -0.59/0.556/-0.07 |
| Insomnia, delayed | 0.68/0.498/0.09 | 0.56/0.575/0.07 | -0.08/0.938/-0.01 | 0.22/0.829/0.02 | 0.34/0.738/0.04 | 0.50/0.614/0.06 | 0.06/0.956/0.01 | 0.20/0.842/0.02 | 0.34/0.731/0.04 | -0.80/0.425/-0.10 | 0.18/0.854/0.02 | 0.20/0.842/0.02 |
| Work and interests | -0.10/0.917/-0.01 | -0.22/0.825/-0.03 | -0.44/0.660/-0.05 | -0.08/0.937/-0.01 | -0.11/0.914/-0.01 | -0.24/0.813/-0.03 | -0.20/0.843/-0.02 | -0.24/0.814/-0.03 | 0.02/0.985/0.00 | 0.44/0.662/0.05 | -0.22/0.823/-0.03 | -0.04/0.967/-0.00 |
| Retardation | 0.40/0.687/0.05 | 1.24/0.217/0.14 | **2.25/0.025/0.28** | 1.27/0.205/0.14 | 1.25/0.213/0.14 | 1.23/0.219/0.15 | 1.19/0.235/0.13 | 1.23/0.220/0.14 | 1.72/0.086/0.19 | 0.55/0.585/0.07 | 1.19/0.234/0.14 | 1.34/0.180/0.15 |
| Agitation | -1.53/0.127/-0.19 | -0.82/0.412/-0.10 | 0.31/0.758/0.04 | -0.91/0.361/-0.10 | -0.78/0.438/-0.09 | -0.75/0.455/-0.09 | -0.49/0.625/-0.05 | -0.76/0.447/-0.08 | -0.55/0.584/-0.06 | -0.89/0.375/-0.11 | -0.91/0.364/-0.10 | -0.16/0.877/-0.02 |
| Anxiety, psychic | **-2.87/0.004/-0.36** | -1.47/0.143/-0.17 | -0.41/0.680/-0.05 | -1.88/0.061/-0.21 | -1.85/0.066/-0.20 | **-2.15/0.032/-0.26** | -1.86/0.064/-0.20 | -1.92/0.055/-0.21 | -1.88/0.061/-0.21 | -1.93/0.054/-0.24 | -1.67/0.096/-0.19 | -1.59/0.113/-0.17 |
| Anxiety, somatic | -1.96/0.051/-0.25 | -0.98/0.329/-0.11 | -0.15/0.880/-0.02 | -1.16/0.246/-0.13 | -1.11/0.269/-0.12 | -0.86/0.390/-0.10 | -1.27/0.206/-0.14 | -1.16/0.247/-0.13 | -1.39/0.167/-0.15 | -1.49/0.138/-0.18 | -0.96/0.338/-0.11 | -1.27/0.205/-0.14 |
| Somatic, gastrointestinal | 0.78/0.438/0.10 | 1.27/0.205/0.15 | 0.69/0.493/0.09 | 0.98/0.327/0.11 | 1.02/0.308/0.11 | 1.25/0.214/0.15 | 1.31/0.190/0.14 | 1.23/0.218/0.14 | 0.98/0.327/0.11 | 0.71/0.479/0.09 | 0.75/0.452/0.09 | 1.23/0.218/0.14 |
| Somatic, general | 0.53/0.594/0.07 | 0.45/0.657/0.05 | 0.82/0.414/0.10 | 0.47/0.642/0.05 | 0.44/0.659/0.05 | 0.35/0.729/0.04 | 0.37/0.709/0.04 | 0.42/0.675/0.05 | 0.32/0.749/0.04 | -0.46/0.644/-0.06 | 0.68/0.496/0.08 | 0.42/0.675/0.05 |
| Genital | -0.30/0.765/-0.04 | -0.85/0.398/-0.10 | -1.11/0.269/-0.14 | -0.41/0.685/-0.05 | -0.51/0.607/-0.06 | 0.50/0.619/0.06 | -0.70/0.485/-0.08 | -0.62/0.534/-0.07 | -0.82/0.411/-0.09 | -0.80/0.425/-0.10 | -0.38/0.704/-0.04 | -0.90/0.369/-0.10 |
| Hypochondriasis | -1.50/0.135/-0.19 | -0.76/0.449/-0.09 | 0.14/0.886/0.02 | -0.99/0.325/-0.11 | -0.98/0.330/-0.11 | -0.96/0.341/-0.11 | -0.94/0.346/-0.10 | -1.08/0.280/-0.12 | -0.87/0.386/-0.10 | -1.36/0.175/-0.17 | -0.84/0.399/-0.10 | -0.73/0.466/-0.08 |
| Loss of weight | 1.23/0.220/0.16 | 1.42/0.156/0.17 | 1.34/0.182/0.17 | 1.30/0.195/0.14 | 1.22/0.225/0.13 | 0.42/0.673/0.05 | 1.25/0.213/0.14 | 1.13/0.259/0.12 | 1.16/0.249/0.13 | 0.61/0.542/0.08 | 1.31/0.192/0.15 | 1.56/0.119/0.17 |
| Insight | -1.09/0.276/-0.14 | -1.95/0.052/-0.23 | -1.96/0.051/-0.24 | -1.83/0.068/-0.20 | -1.92/0.056/-0.21 | -1.82/0.070/-0.22 | -1.28/0.202/-0.14 | -1.64/0.102/-0.18 | -1.12/0.265/-0.12 | -0.93/0.354/-0.11 | -1.67/0.097/-0.19 | -1.11/0.268/-0.12 |
| Association between HAMD-17 total score and illness duration (years) |  |  |  |  |  |  |  |  |  |  |  |  |
|  |  |  |  |  |  |  |  |  |  |  |  |  |
|  |  |  |  |  |  |  |  |  |  |  |  |  |
|  |  |  |  |  |  |  |  |  |  |  |  |  |
| Illness duration (years) | **7.02/0.008/0.00** | **13.26/< 0.001/0.00** | **14.39/< 0.001/0.01** | **13.22/< 0.001/0.00** | **13.64/< 0.001/0.00** | **12.10/< 0.001/0.00** | **12.17/< 0.001/0.00** | **13.21/< 0.001/0.00** | **18.54/< 0.001/0.00** | 1.42/0.233/0.00 | **15.01/< 0.001/0.00** | **13.20/< 0.001/0.00** |
|  |  |  |  |  |  |  |  |  |  |  |  |  |
|  |  |  |  |  |  |  |  |  |  |  |  |  |
| Post-hoc (Subtype 1) | **-0.19/0.008** | **-0.22/0.001** | **-0.26/< 0.001** | **-0.22/< 0.001** | **-0.23/< 0.001** | **-0.22/0.001** | **-0.22/< 0.001** | **-0.22/< 0.001** | **-0.31/< 0.001** | **-** | **-0.22/< 0.001** | **-0.20/0.002** |
| Post-hoc (Subtype 2) | -0.07/0.194 | **-0.11/0.037** | -0.09/0.070 | -0.10/0.052 | **-0.10/0.044** | -0.10/0.060 | -0.09/0.076 | **-0.10/0.046** | **-0.15/0.013** | **-** | **-0.11/0.024** | **-0.11/0.030** |
| Data are presented as the t, χ2, F, or r/p/Cohen’s d, Cramer's V or Partial η2. Abbreviations: PKU, Peking University; ZJU, Zhejiang University; CMU, China Medical University; JNU, Jinan University; SXMU, Shanxi Medical University; CSU, Central South University; SEU, Southeast University; CQMU, Chongqing Medical University; SWU, Southwest University; CCMU, Capital Medical University; SCU, Sichuan University; HAMD-17, Hamilton Depression Rating Scale-17 item; HAMA-14, Hamilton Anxiety Rating Scale-14 item. | | | | | | | | | | | | |
|  |  |  |  |  |  |  |  |  |  |  |  |  |
|  |  |  |  |  |  |  |  |  |  |  |  |  |
|  |  |  |  |  |  |  |  |  |  |  |  |  |

| Table S15. Validation of clustering robustness across different algorithms | | | | |
| --- | --- | --- | --- | --- |
| Comparison | Algorithm A | Algorithm B | Label overlap (%) | Adjusted rand index (ARI) |
| Primary validation | K-means (Primary) | Gaussian mixture model (GMM) | 0.98 | 0.93 |
|  | K-means (Primary) | Hierarchical clustering (HC) | 0.88 | 0.58 |
|  | K-means (Primary) | Spectral clustering | 0.65 | 0.13 |
| Clustering solutions were derived from the same individual deviation maps. | | | | |


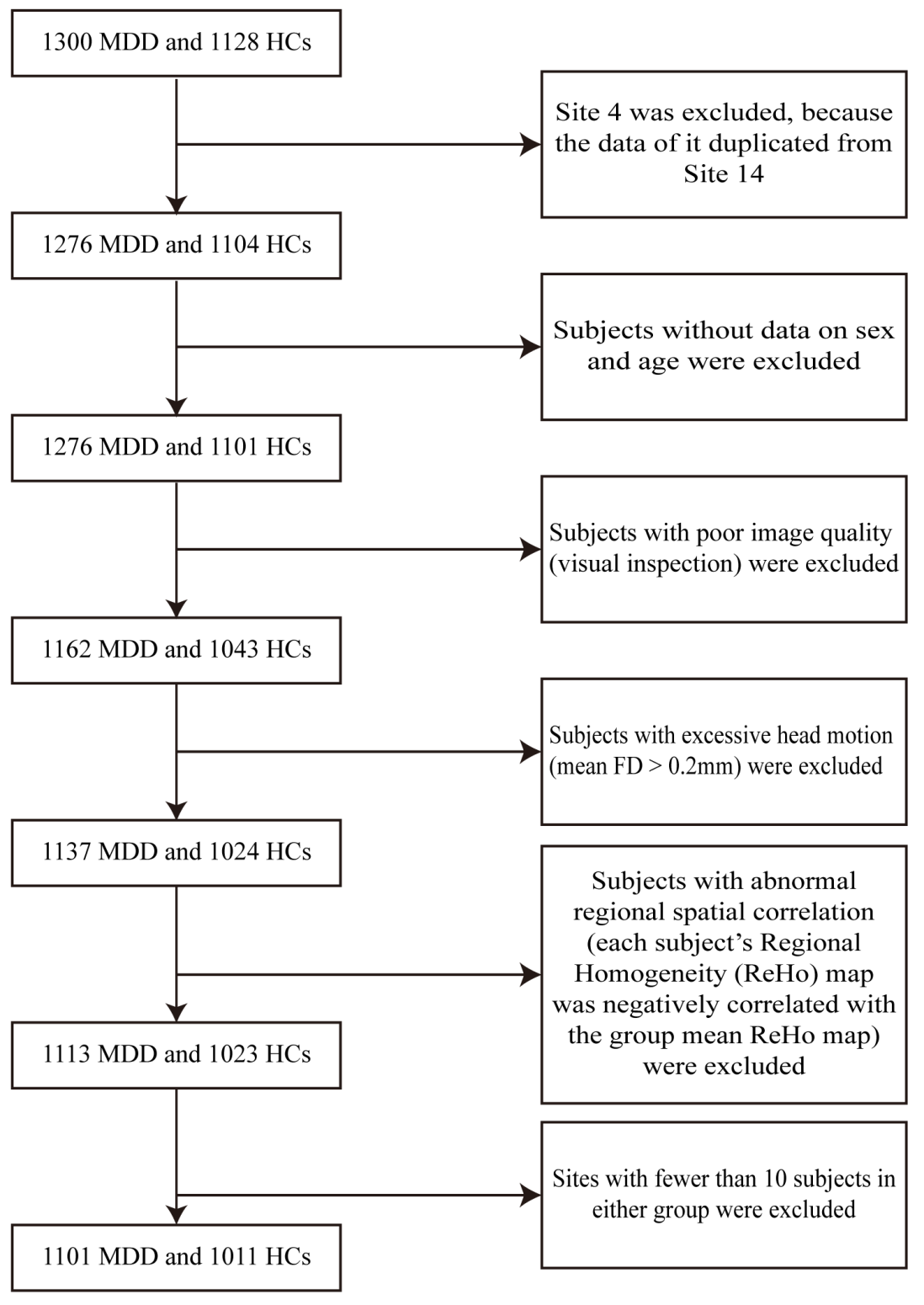


Figure S1. Workflow for screening participants. MDD, major depressive disorder; HCs, healthy controls.


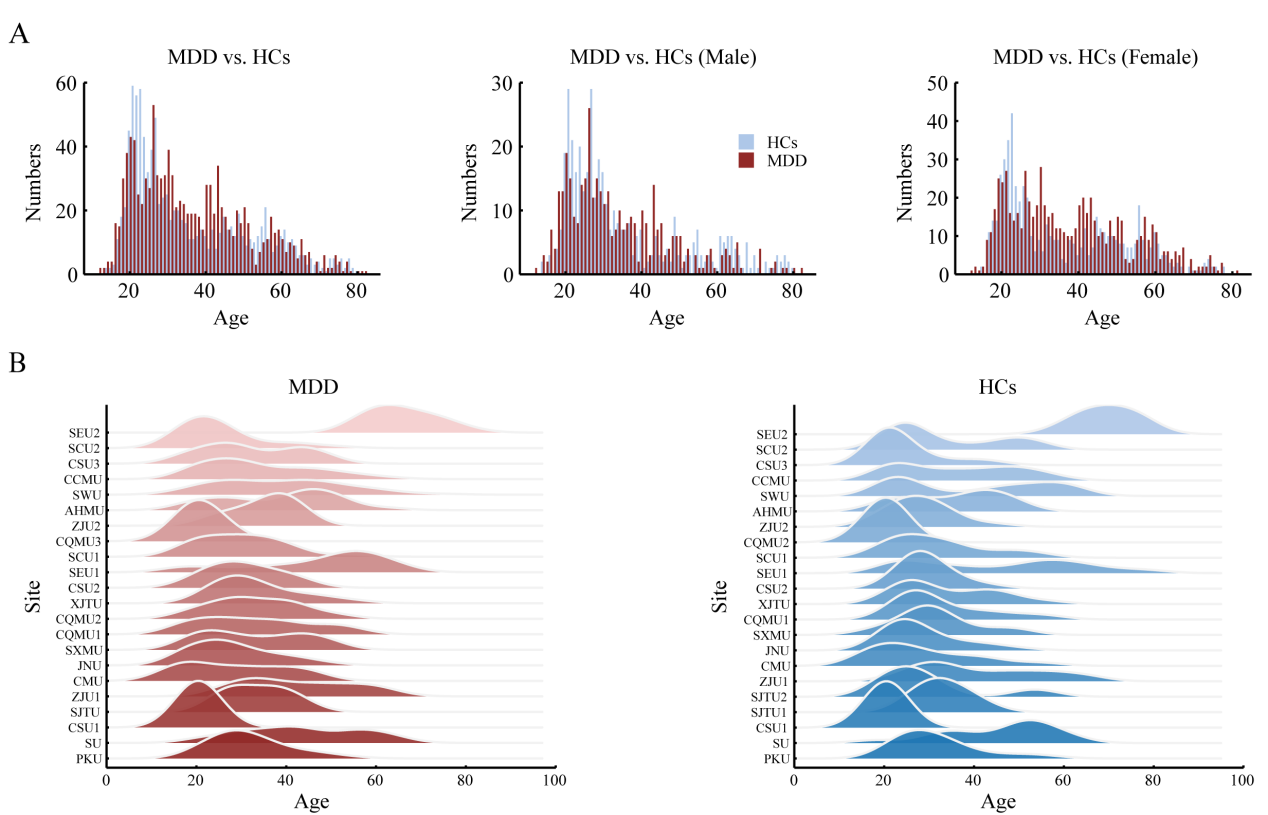


Figure S2. Age distribution of participants. (A) Numbers of all patients with MDD and HCs for each age and the sex-specific age distribution in both groups. (B) Age distribution of patients with MDD and HCs at each site. Sites: PKU, Peking University; SU, Soochow University; CSU, Central South University; SJTU, Shanghai Jiao Tong University; ZJU, Zhejiang University; CMU, China Medical University; JNU, Jinan University; SXMU; Shanxi Medical University; CQMU, Chongqing Medical University; XJTU, Xi’an Jiaotong University; SEU, Southeast University; SCU, Sichuan University; AHMU, Anhui Medical University; SWU, Southwest University; CCMU, Capital Medical University.


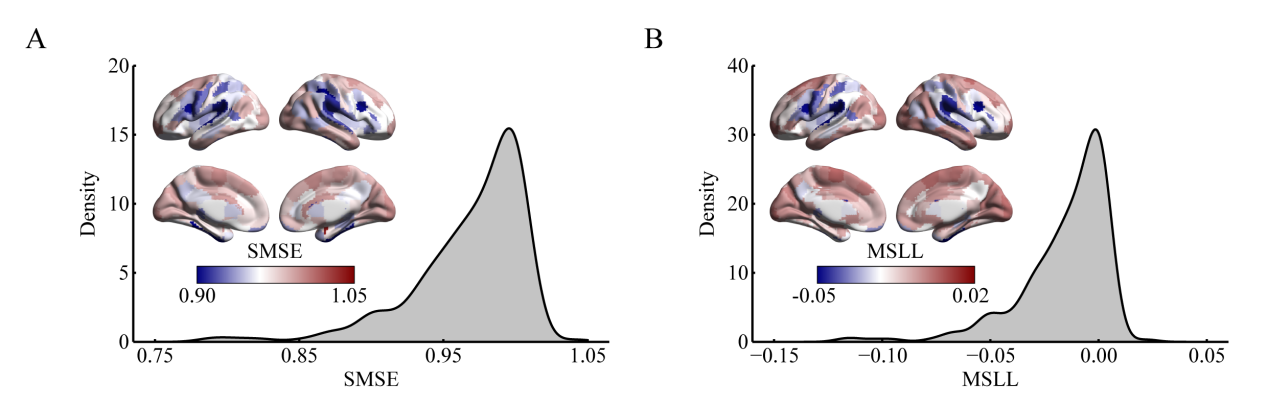


Figure S3. Evaluation of the normative model fit. (A) Distribution and brain maps of standardized mean squared error (SMSE) comparing true and predicted mean ReHo values in HCs under 10-fold cross-validation. (B) Distribution and brain maps of mean standardized log loss (MSLL) comparing true and predicted mean ReHo values in HCs under 10-fold cross-validation.


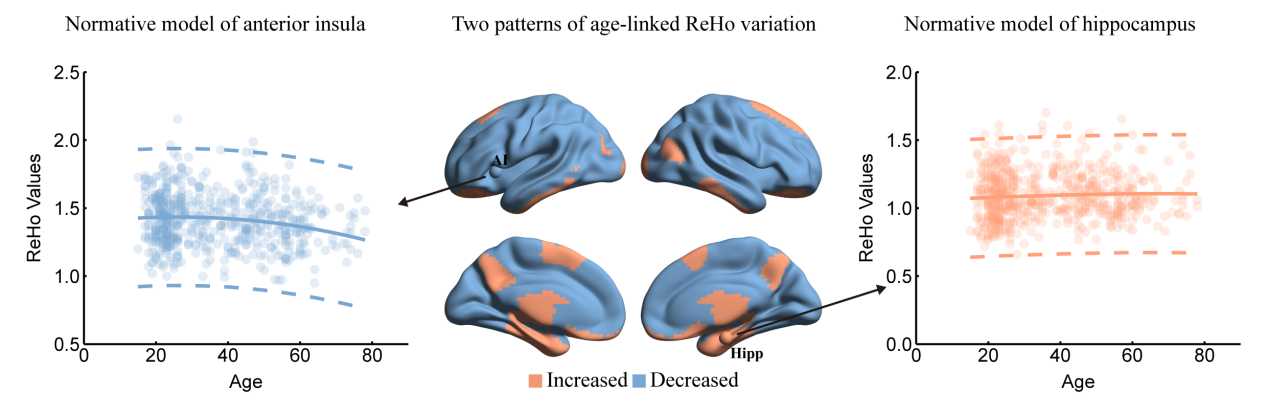


Figure S4. Normative model established in HCs. Age-related ReHo variation in HCs (female) is shown, categorized into decreasing (blue) and increasing (orange) patterns using k-means clustering. Scatter plots display true ReHo value for the anterior insula (left) and hippocampus (right) in HCs, with the solid line representing the predicted mean and dashed lines showing the normative ranges.


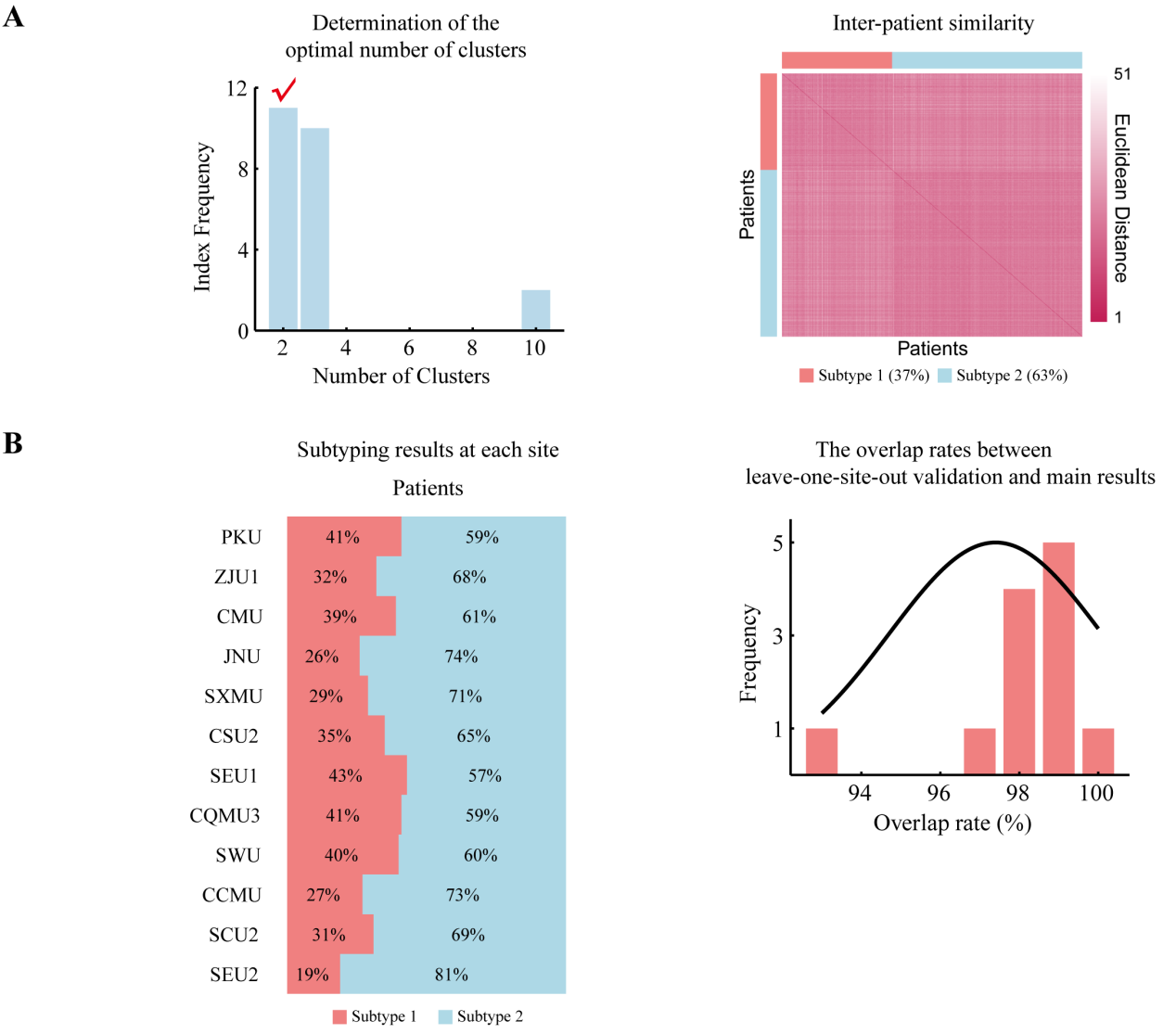


Figure S5. Identification of MDD subtypes after excluding sites with fewer than 30 patients. (A) The optimal number of MDD clusters remains 2, determined by the NbClust package, with inter-patient similarity assessed by Euclidean distance. (B) Subtyping results from k-means clustering at each site, showing the overlap rates (rounded) between site-specific clustering labels and primary results from leave-one-site-out validation. Sites: PKU, Peking University; ZJU, Zhejiang University; CMU, China Medical University; JNU, Jinan University; SXMU, Shanxi Medical University; CSU, Central South University; SEU, Southeast University; CQMU, Chongqing Medical University; SWU, Southwest University; CCMU, Capital Medical University; SCU, Sichuan University.


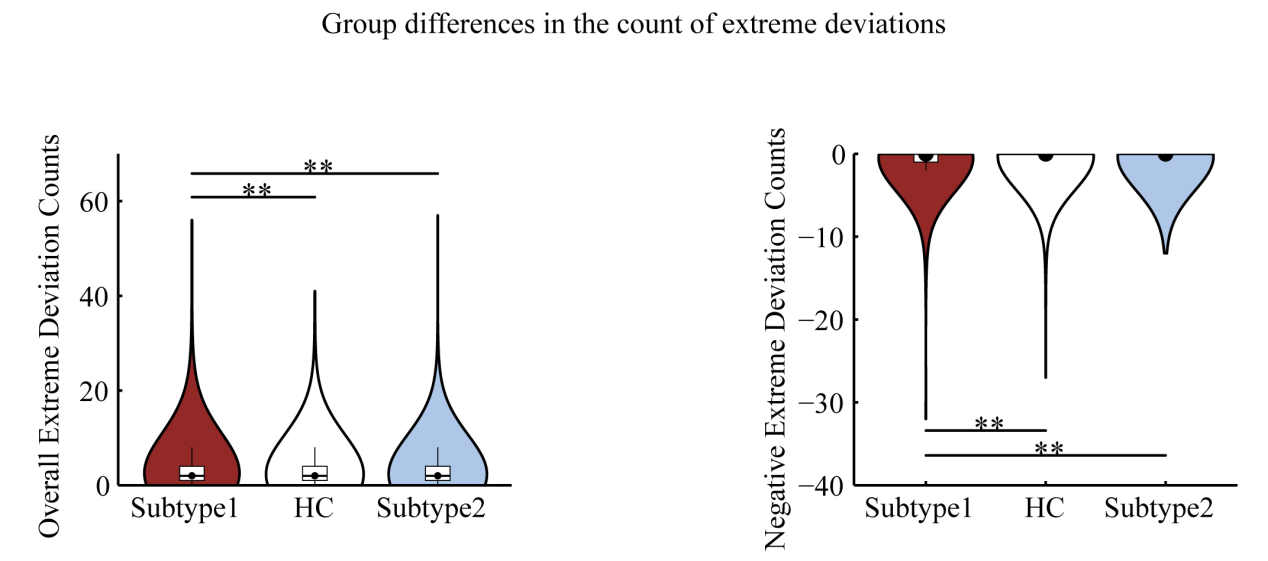
Figure S6. Group differences in extreme deviation counts among MDD subtypes and HCs. ** FDR- corrected *p* < 0.01.


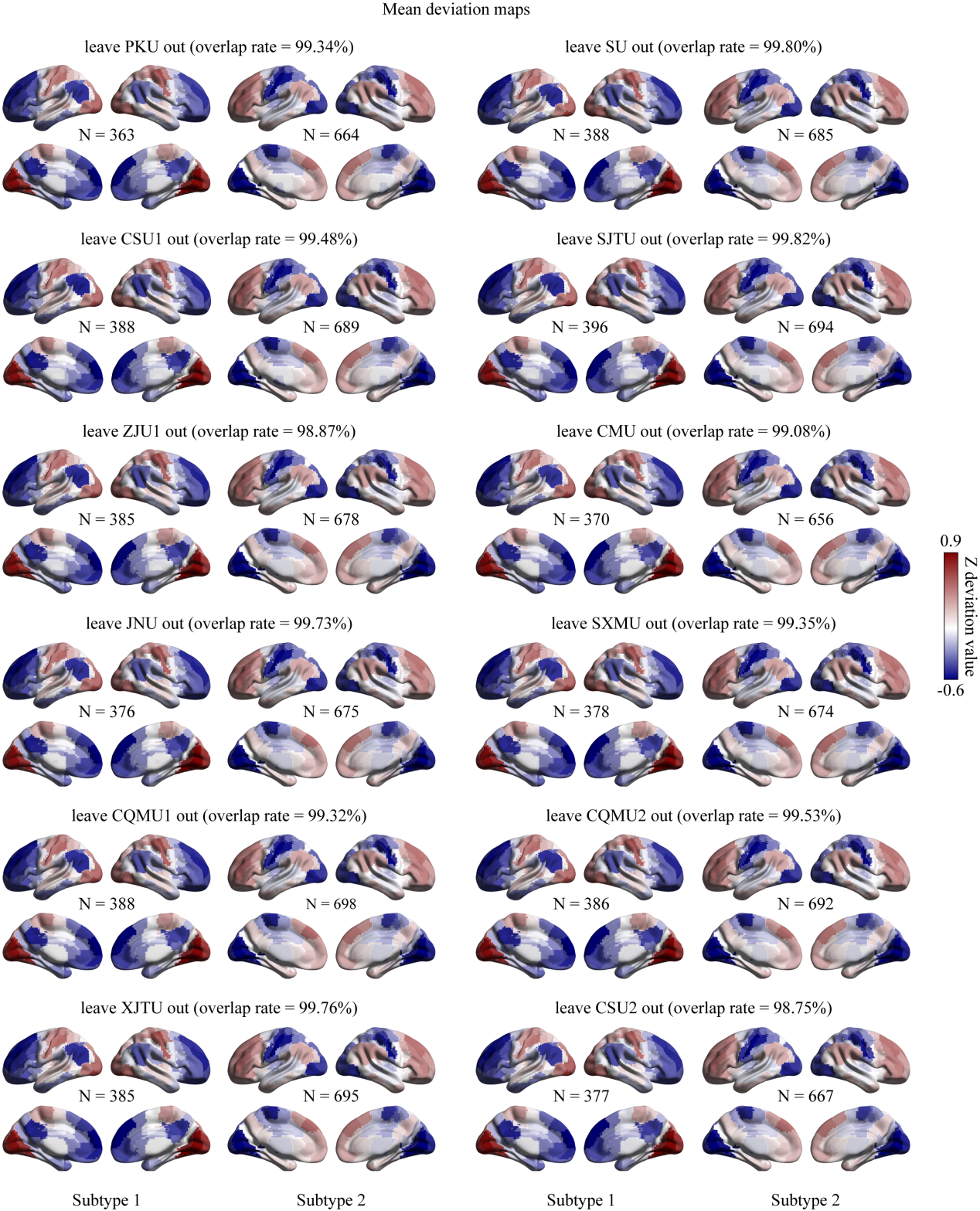


Figure S7.


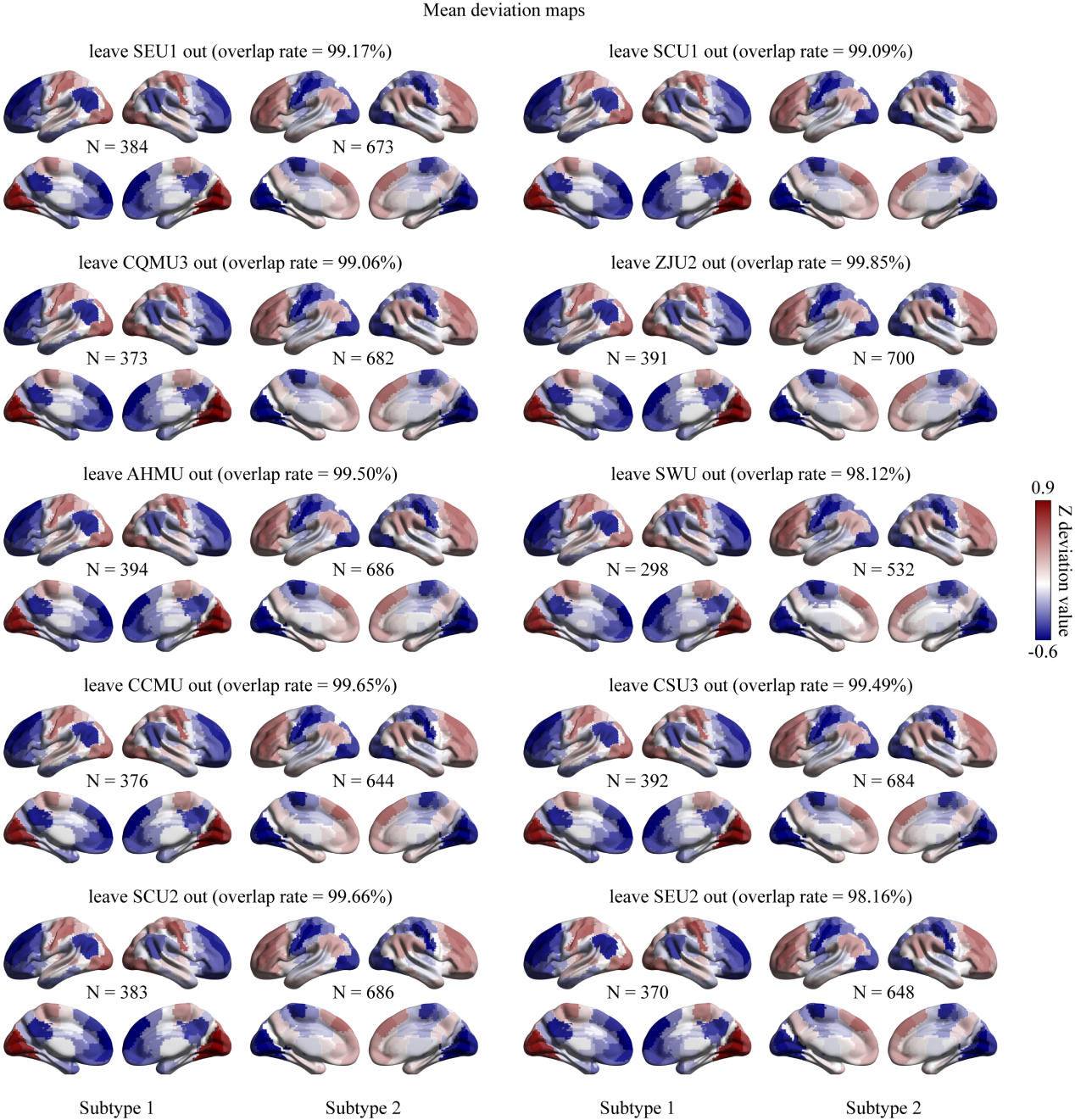


Figure S7 (continued). Mean deviation maps from leave-one-site-out validation and overlap rates between new clustering labels and the primary results. As shown in Figure 3 (B), all overlap rates are > 98%.


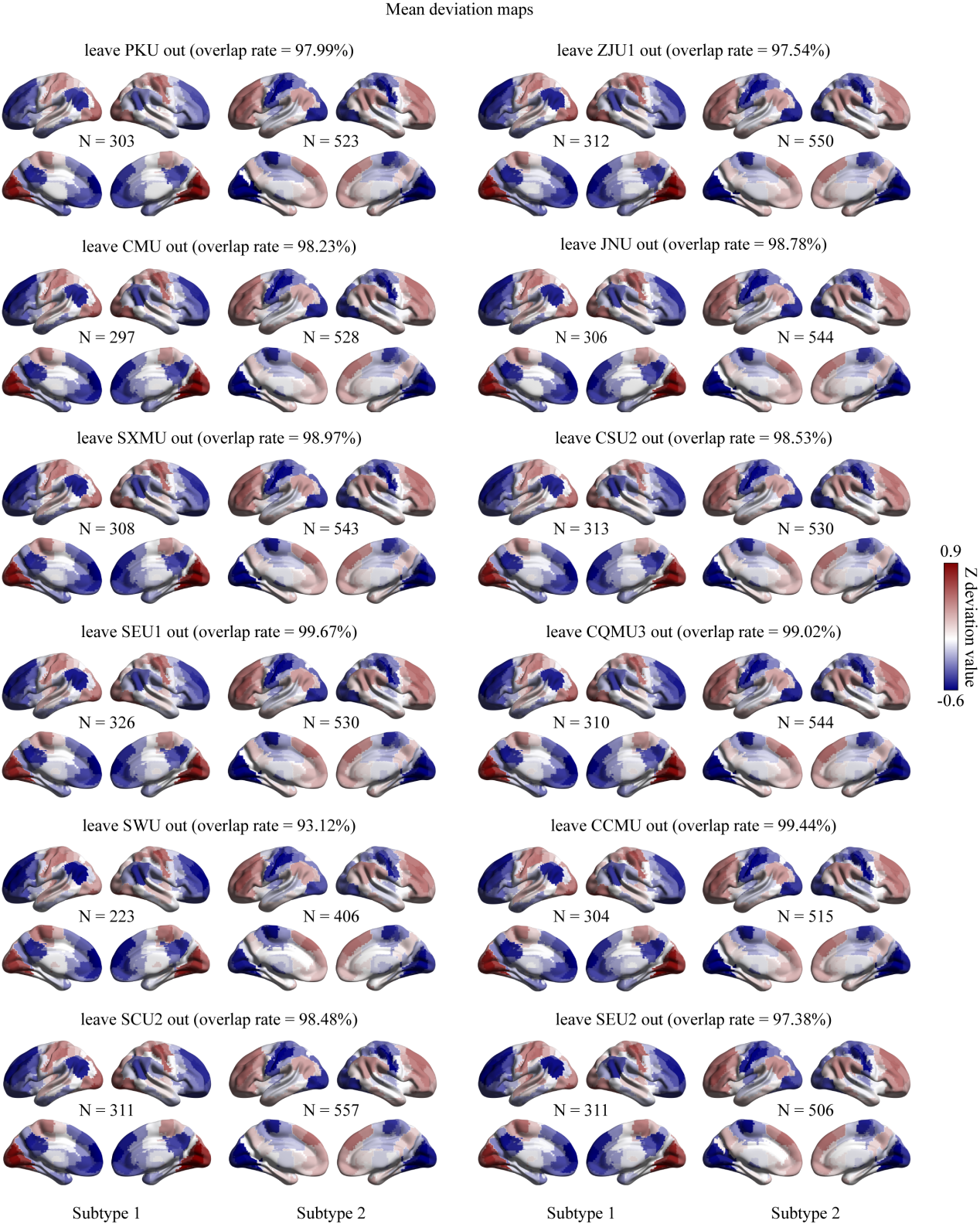


Figure S8. Mean deviation maps from leave-one-site-out validation and overlap rates between new clustering labels and the primary results (sites with more than 30 patients). As shown in Figure S4 (B), all overlap rates are > 94%.


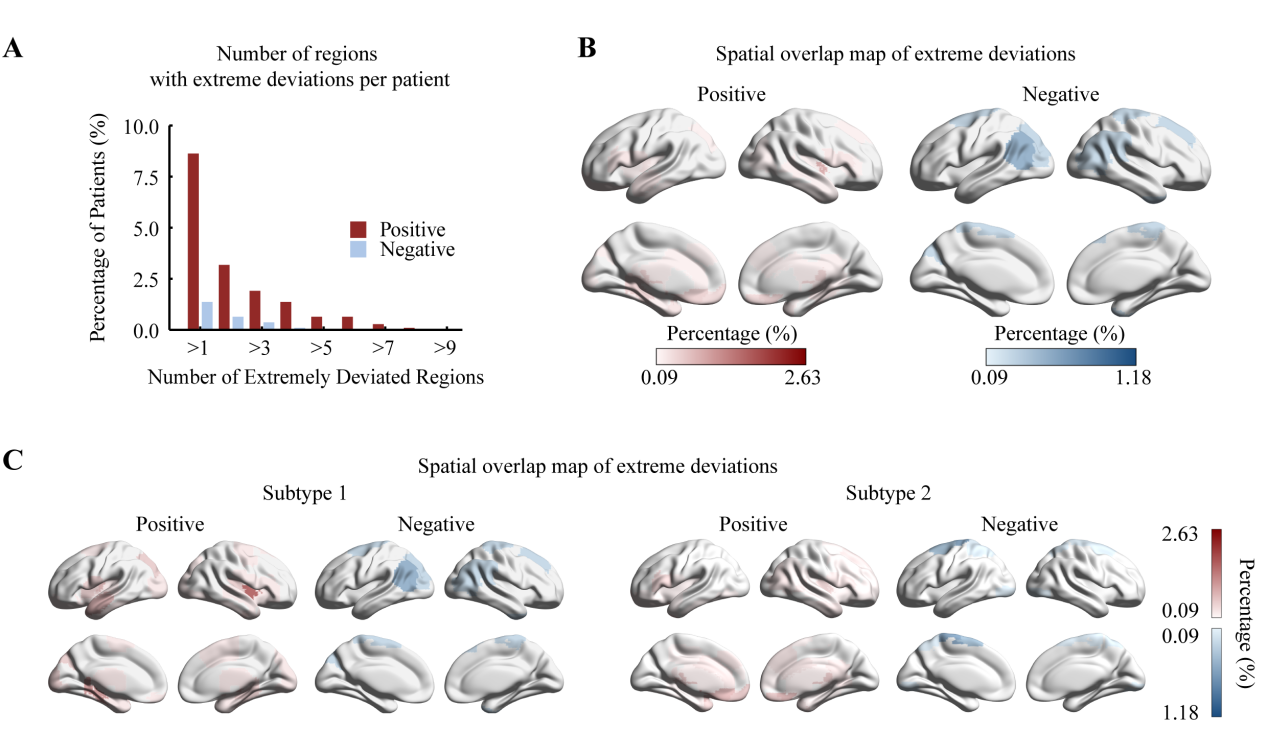


Figure S9. Characterization of extreme deviations with FDR correction. (A) Number of brain regions with extreme deviations per patient. (B) Spatial overlap map showing extreme positive (red) and negative (blue) deviations across all patients with MDD. (C) Spatial overlap map of extreme positive (red) and negative (blue) deviations for each subtype.


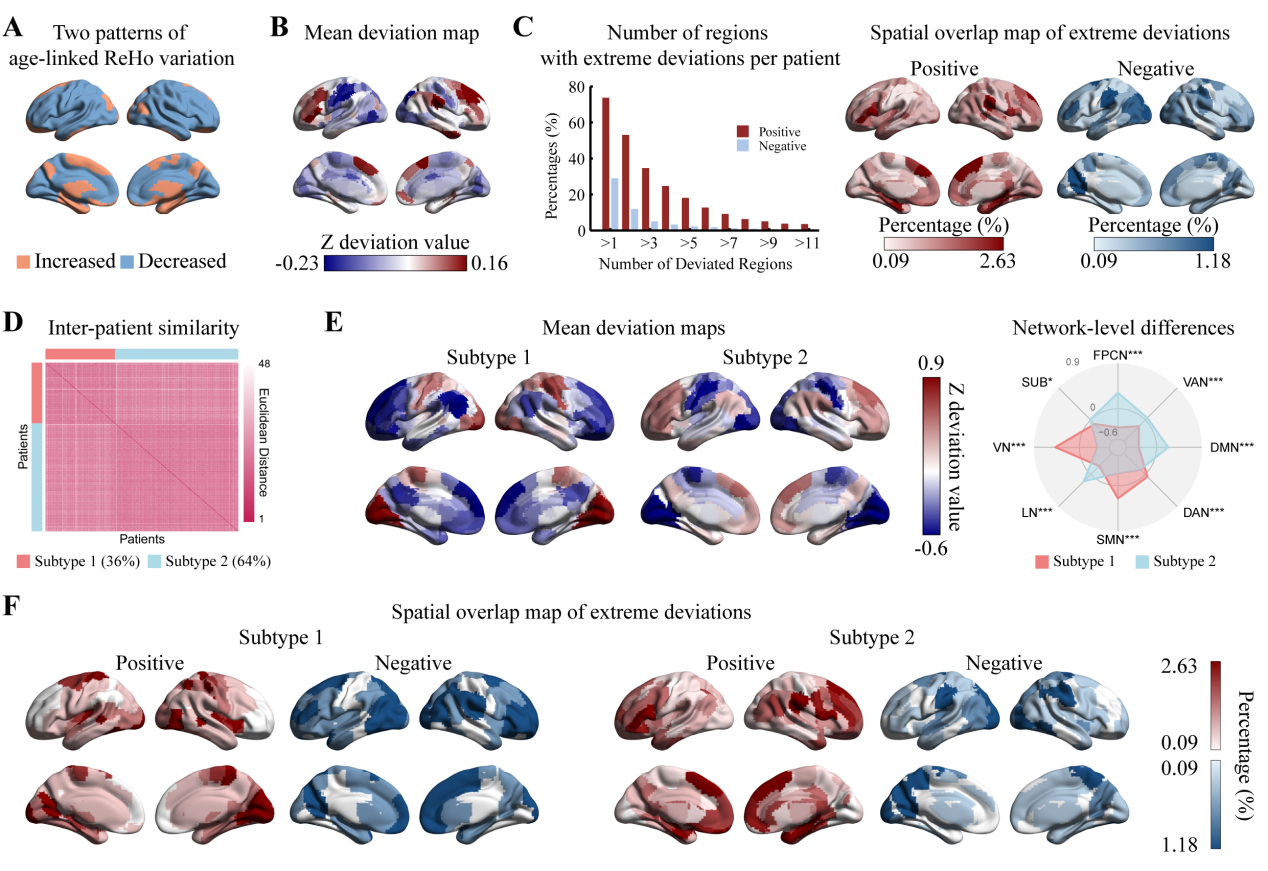


Figure S10. Validation analysis in male group. (A) Brain regions classified into two distinct patterns based on age-related ReHo variation using k-means clustering. (B) Mean deviation maps for all patients with MDD. (C) Number of brain regions with extreme deviations per patient and spatial overlap map showing extreme positive (red) and negative (blue) deviations across all patients with MDD. (D) Proportion of patients in each subtype and inter-patient similarity was assessed by Euclidean distance. (E) Mean deviation maps for each subtype and their network-level differences. (F) Spatial overlap map of extreme positive (red) and negative (blue) deviations for each subtype. *** FDR-corrected *p* < 0.001, * *p* < 0.05. Networks: FPCN, frontoparietal control network; VAN, ventral attention network; DMN, default mode network; DAN, dorsal attention network; SMN, sensorimotor network; LN, limbic network; VN, visual network; SUB, subcortical regions.


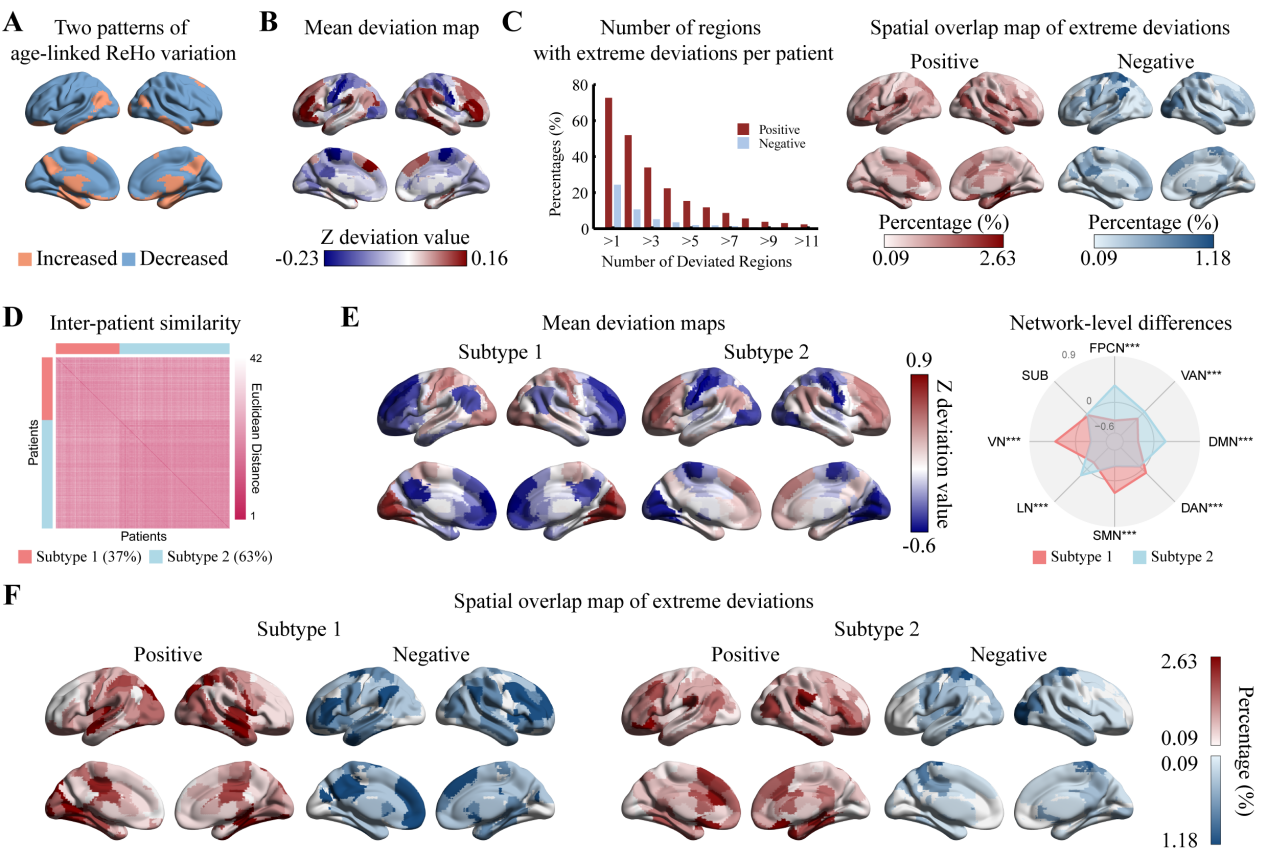


Figure S11. Validation analysis in female group. (A) Brain regions classified into two distinct patterns based on age-related ReHo variation using k-means clustering. (B) Mean deviation map for all patients with MDD. (C) Number of brain regions with extreme deviations per patient and spatial overlap map showing extreme positive (red) and negative (blue) deviations across all patients with MDD. (D) Proportion of patients in each subtype and inter-patient similarity was assessed by Euclidean distance. (E) Mean deviation maps for each subtype and their network-level differences. (F) Spatial overlap map of extreme positive (red) and negative (blue) deviations for each subtype. *** FDR-corrected *p* < 0.001. Networks: FPCN, frontoparietal control network; VAN, ventral attention network; DMN, default mode network; DAN, dorsal attention network; SMN, sensorimotor network; LN, limbic network; VN, visual network; SUB, subcortical regions.


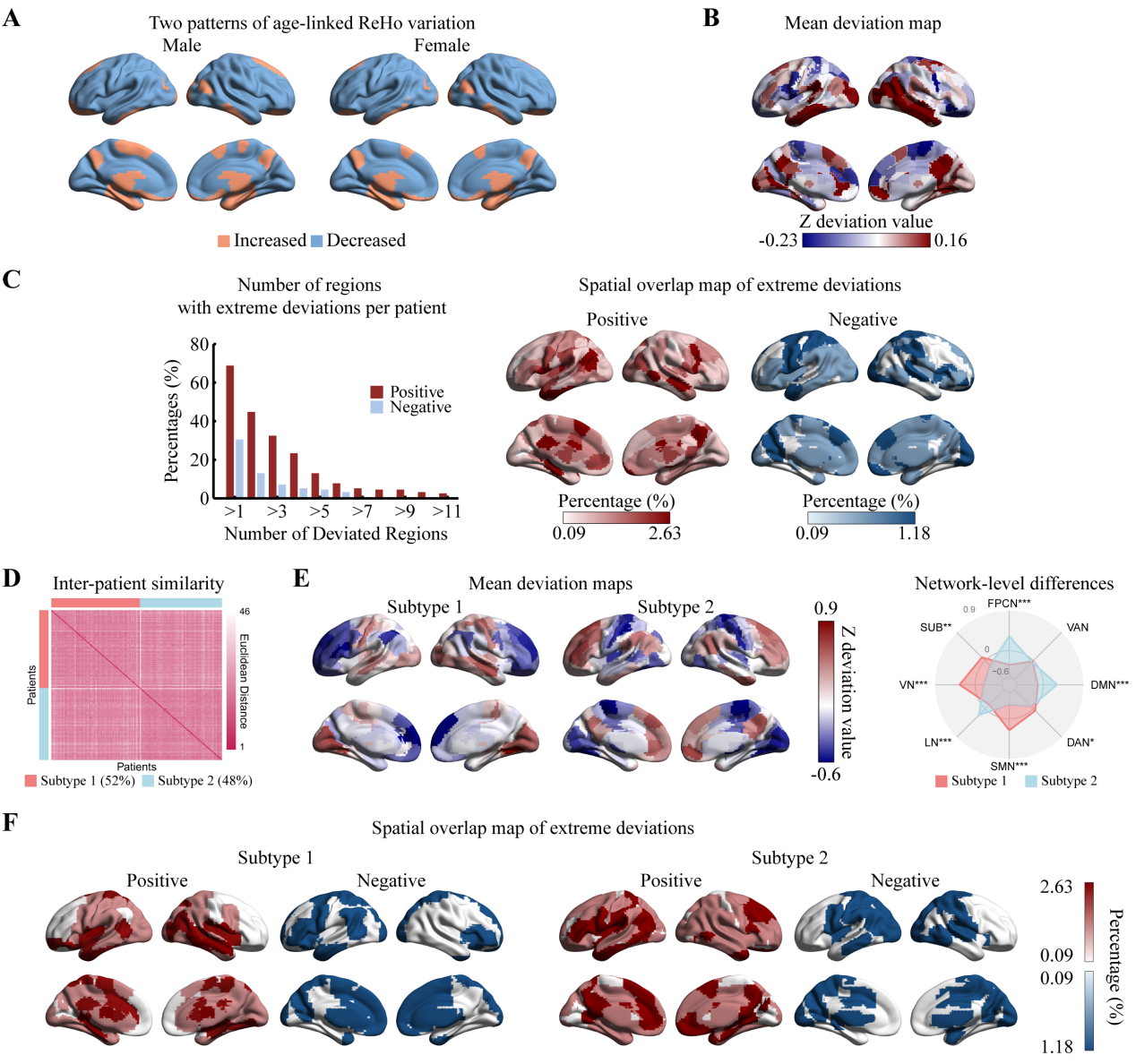


Figure S12. Validation analysis in young group. (A) Brain regions classified into two distinct patterns based on the combined trajectories of age-related ReHo variation from young and old groups using k-means clustering. (B) Mean deviation map for all patients with MDD. (C) Number of brain regions with extreme deviations per patient and spatial overlap map showing extreme positive (red) and negative (blue) deviations across all patients with MDD. (D) Proportion of patients in each subtype and inter-patient similarity was assessed by Euclidean distance. (E) Mean deviation maps for each subtype and their network-level differences. (F) Spatial overlap map of extreme positive (red) and negative (blue) deviations for each subtype. *** FDR-corrected *p* < 0.001, ** *p* < 0.01, * *p* < 0.05. Networks: FPCN, frontoparietal control network; VAN, ventral attention network; DMN, default mode network; DAN, dorsal attention network; SMN, sensorimotor network; LN, limbic network; VN, visual network; SUB, subcortical regions.


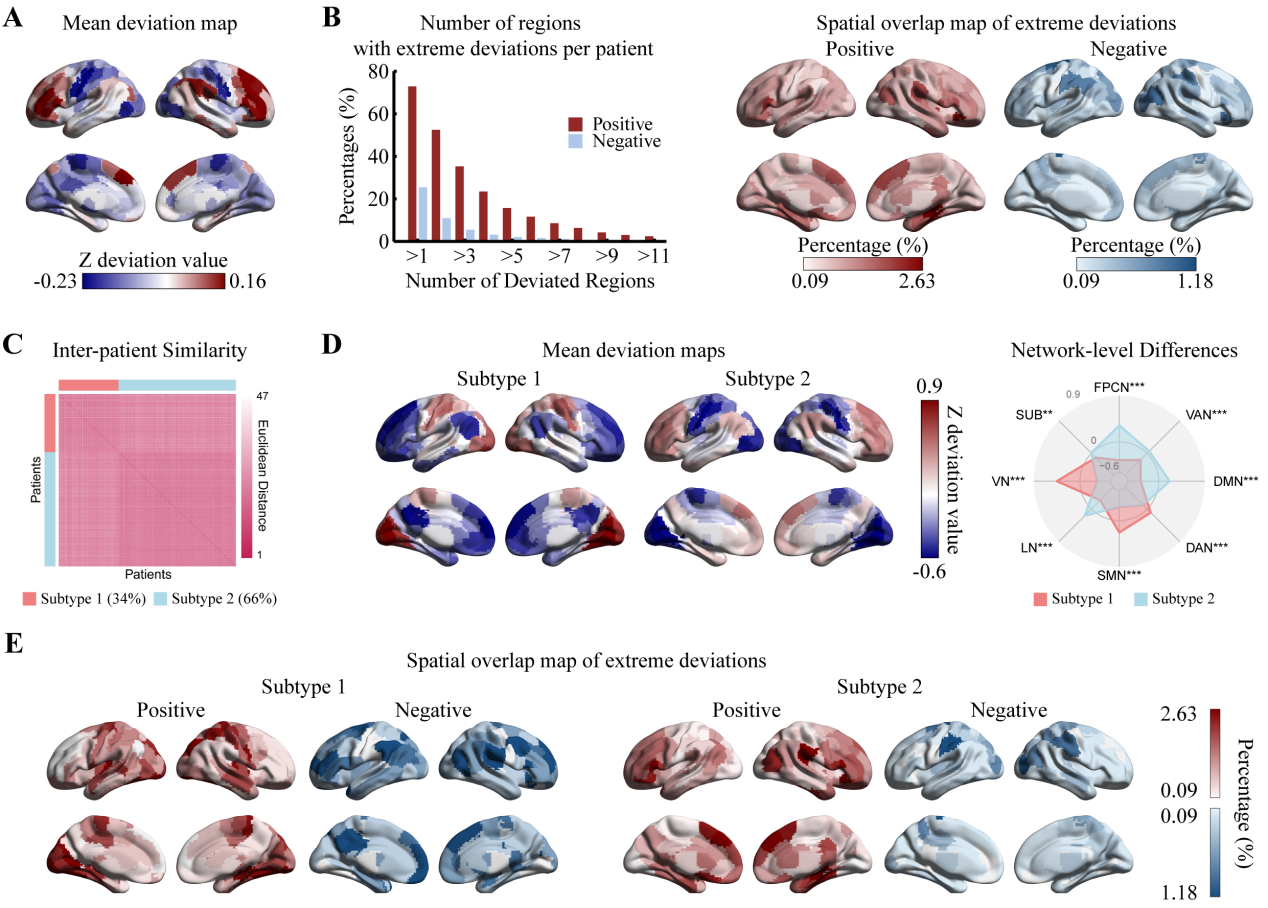


Figure S13. Validation analysis in old group. (A) Mean deviation map for all patients with MDD. (B) Number of brain regions with extreme deviations per patient and spatial overlap map showing extreme positive (red) and negative (blue) deviations across all patients with MDD. (C) Proportion of patients in each subtype and inter-patient similarity was assessed by Euclidean distance. (D) Mean deviation maps for each subtype and their network-level differences. (E) Spatial overlap map of extreme positive (red) and negative (blue) deviations for each subtype. *** FDR-corrected *p* < 0.001, ** *p* < 0.01. Networks: FPCN, frontoparietal control network; VAN, ventral attention network; DMN, default mode network; DAN, dorsal attention network; SMN, sensorimotor network; LN, limbic network; VN, visual network; SUB, subcortical regions.


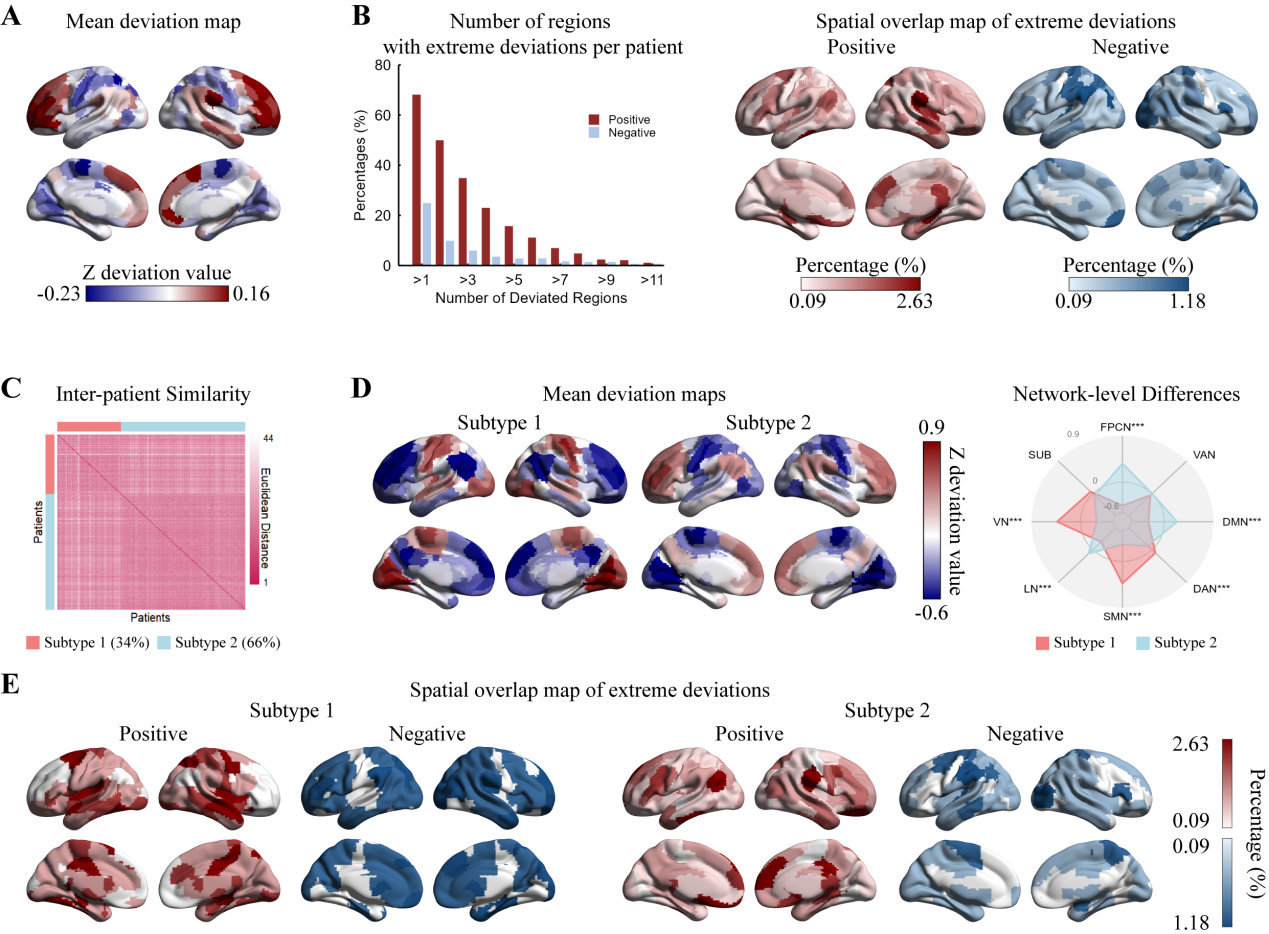


Figure S14. Validation analysis in first-episode drug-naïve group. (A) Mean deviation map for all patients with MDD. (B) Number of brain regions with extreme deviations per patient and spatial overlap map showing extreme positive (red) and negative (blue) deviations across all patients with MDD. (C) Proportion of patients in each subtype and inter-patient similarity was assessed by Euclidean distance. (D) Mean deviation maps for each subtype and their network-level differences. (E) Spatial overlap map of extreme positive (red) and negative (blue) deviations for each subtype. *** FDR-corrected *p* < 0.001. Networks: FPCN, frontoparietal control network; VAN, ventral attention network; DMN, default mode network; DAN, dorsal attention network; SMN, sensorimotor network; LN, limbic network; VN, visual network; SUB, subcortical regions.


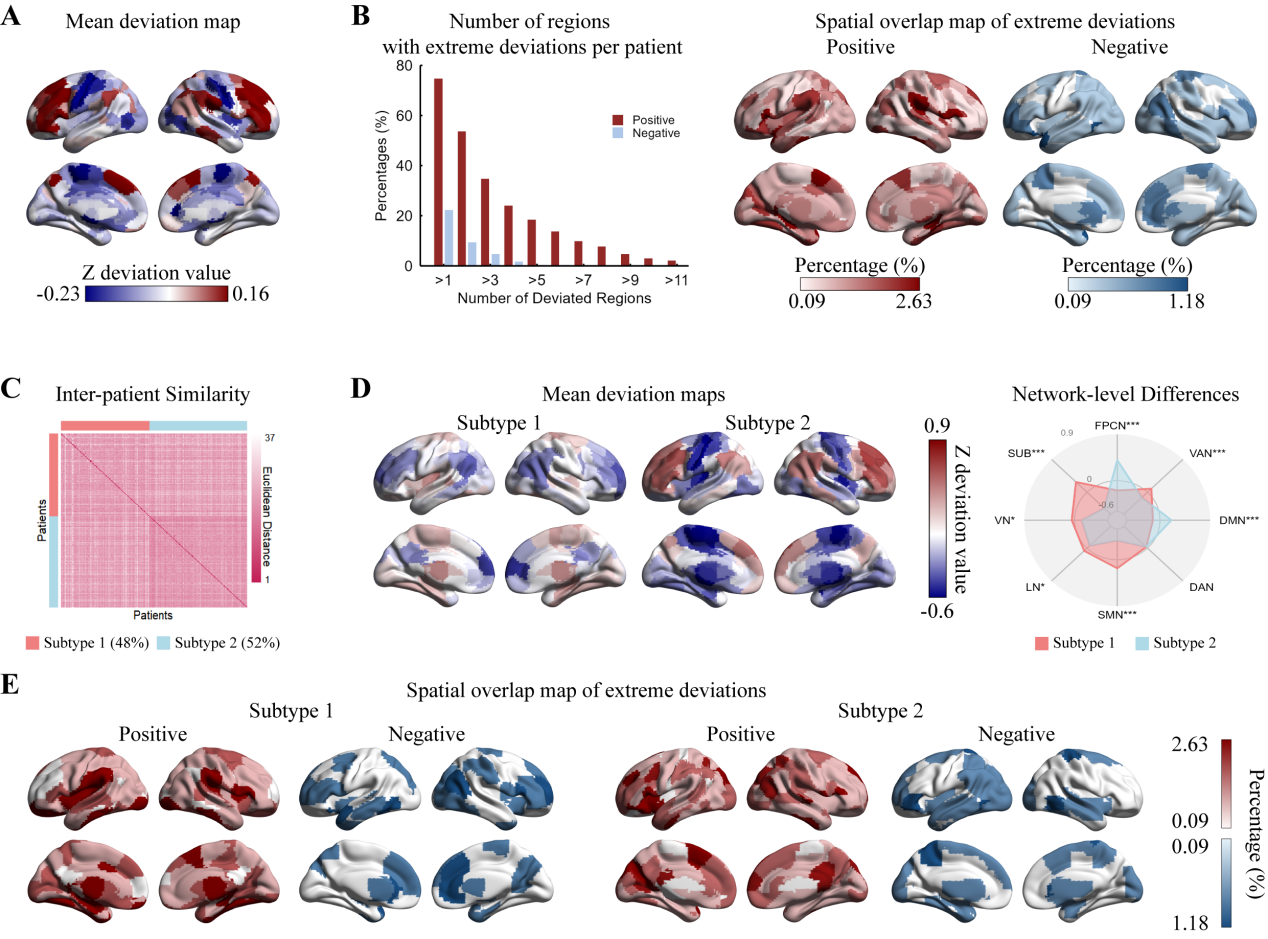


Figure S15. Validation analysis in recurrent group. (A) Mean deviation map for all patients with MDD. (B) Number of brain regions with extreme deviations per patient and spatial overlap map showing extreme positive (red) and negative (blue) deviations across all patients with MDD. (C) Proportion of patients in each subtype and inter-patient similarity was assessed by Euclidean distance. (D) Mean deviation maps for each subtype and their network-level differences. (E) Spatial overlap map of extreme positive (red) and negative (blue) deviations for each subtype. *** FDR-corrected *p* < 0.001, * FDR-corrected *p* < 0.05. Networks: FPCN, frontoparietal control network; VAN, ventral attention network; DMN, default mode network; DAN, dorsal attention network; SMN, sensorimotor network; LN, limbic network; VN, visual network; SUB, subcortical regions.


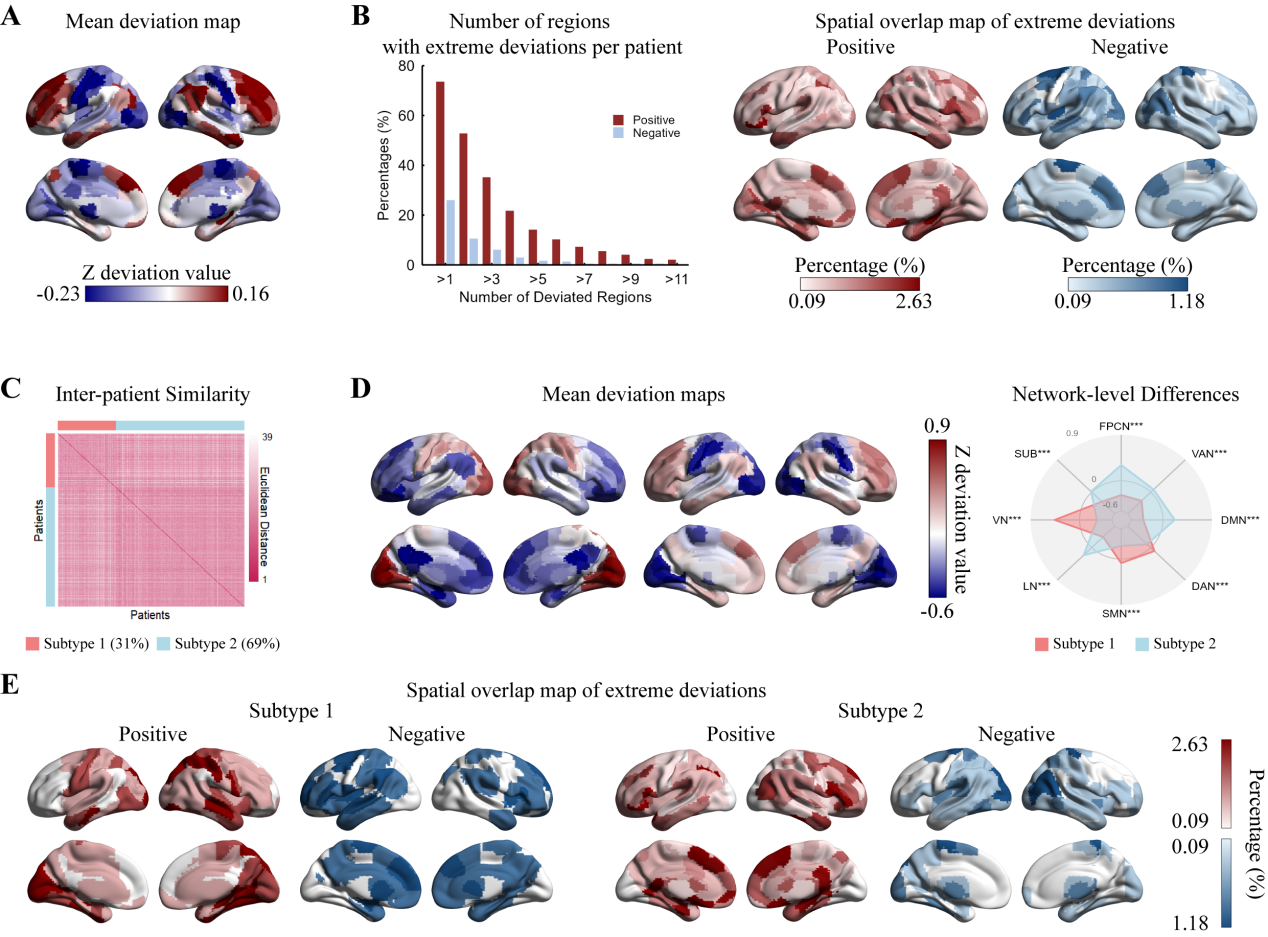


Figure S16. Validation analysis in medicated group. (A) Mean deviation map for all patients with MDD. (B) Number of brain regions with extreme deviations per patient and spatial overlap map showing extreme positive (red) and negative (blue) deviations across all patients with MDD. (C) Proportion of patients in each subtype and inter-patient similarity was assessed by Euclidean distance. (D) Mean deviation maps for each subtype and their network-level differences. (E) Spatial overlap map of extreme positive (red) and negative (blue) deviations for each subtype. *** FDR-corrected *p* < 0.001. Networks: FPCN, frontoparietal control network; VAN, ventral attention network; DMN, default mode network; DAN, dorsal attention network; SMN, sensorimotor network; LN, limbic network; VN, visual network; SUB, subcortical regions.


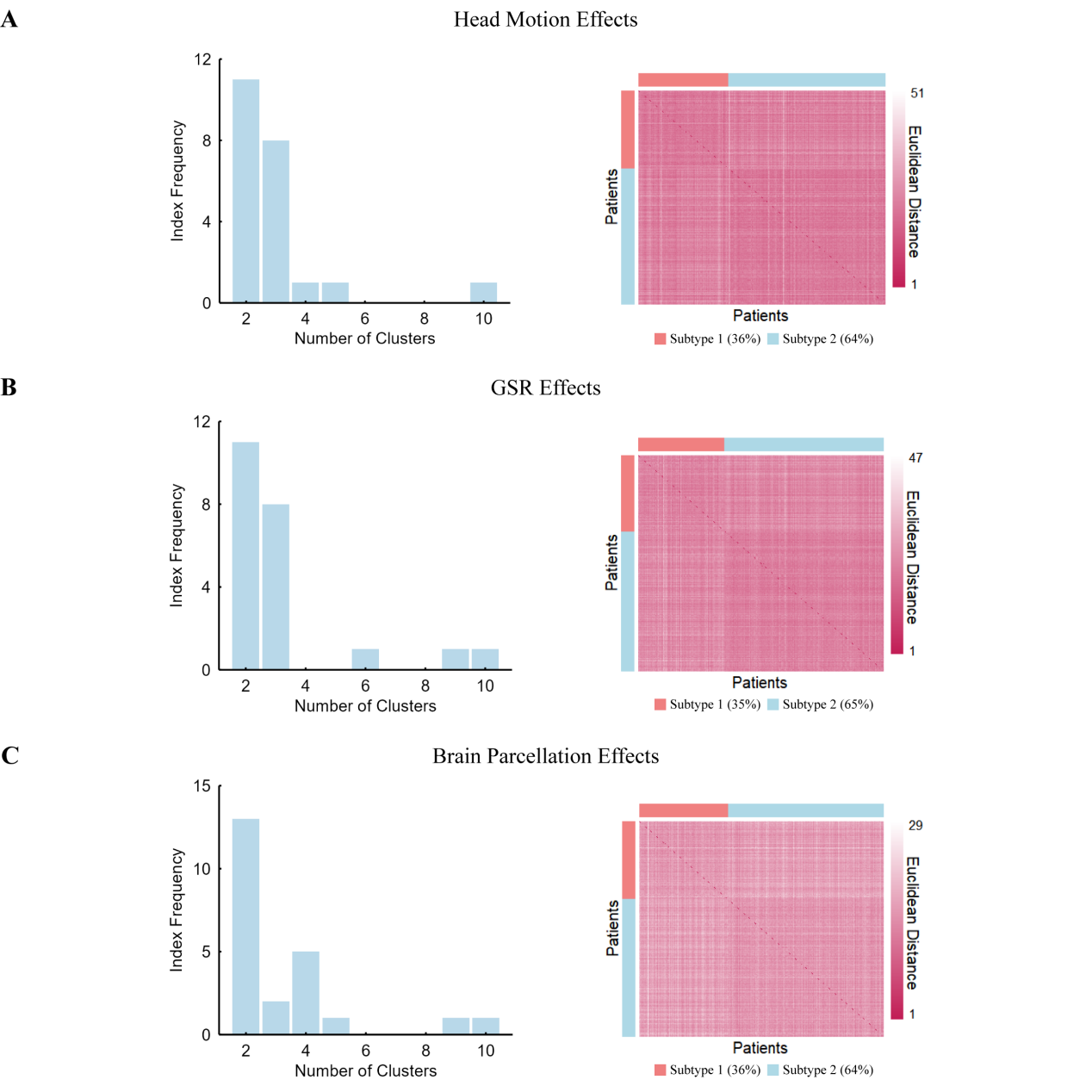


Figure S17. Validation analyses evaluating the robustness of the MDD subtype solution. The optimal number of MDD clusters was determined using the NbClust package, with inter-patient similarity assessed by Euclidean distance. (A) Head motion effects on results. (B) Global signal regression Effects on Results. (C) Brain Parcellation Effects on Results.
